# Mechanisms of IR amplification in radical cation polarons†

**DOI:** 10.1039/c9sc05717j

**Published:** 2020-01-22

**Authors:** William J. Kendrick, Michael Jirásek, Martin D. Peeks, Gregory M. Greetham, Igor V. Sazanovich, Paul M. Donaldson, Michael Towrie, Anthony W. Parker, Harry L. Anderson

**Affiliations:** Department of Chemistry, University of Oxford, Chemistry Research Laboratory Oxford OX1 3TA UK harry.anderson@chem.ox.ac.uk; Central Laser Facility, Research Complex at Harwell, Science and Technology Facilities Council Didcot OX11 0QX UK tony.parker@stfc.ac.uk

## Abstract

Break down of the Born–Oppenheimer approximation is caused by mixing of electronic and vibrational transitions in the radical cations of some conjugated polymers, resulting in unusually intense vibrational bands known as infrared active vibrations (IRAVs). Here, we investigate the mechanism of this amplification, and show that it provides insights into intramolecular charge migration. Spectroelectrochemical time-resolved infrared (TRIR) and two-dimensional infrared (2D-IR) spectroscopies were used to investigate the radical cations of two butadiyne-linked conjugated porphyrin oligomers, a linear dimer and a cyclic hexamer. The 2D-IR spectra reveal strong coupling between all the IRAVs and the electronic π–π* polaron band. Intramolecular vibrational energy redistribution (IVR) and vibrational relaxation occur within ∼0.1–7 ps. TRIR spectra show that the transient ground state bleach (GSB) and excited state absorption (ESA) signals have anisotropies of 0.31 ± 0.07 and 0.08 ± 0.04 for the linear dimer and cyclic hexamer cations, respectively. The small TRIR anisotropy for the cyclic hexamer radical cation indicates that the vibrationally excited polaron migrates round the nanoring on a time scale faster than the measurement, *i.e.* within 0.5 ps, at 298 K. Density functional theory (DFT) calculations qualitatively reproduce the emergence of the IRAVs. The first singlet (S_1_) excited states of the neutral porphyrin oligomers exhibit similar IRAVs to the radical cations, implying that the excitons have similar electronic structures to polarons. Our results show that IRAVs originate from the strong coupling of charge redistribution to nuclear motion, and from the similar energies of electronic and vibrational transitions.

## Abstract 2

Break down of the Born–Oppenheimer approximation is caused by mixing of electronic and vibrational transitions in the radical cations of some conjugated polymers, resulting in unusually intense vibrational bands known as infrared active vibrations (IRAVs).

## Graphical abstract

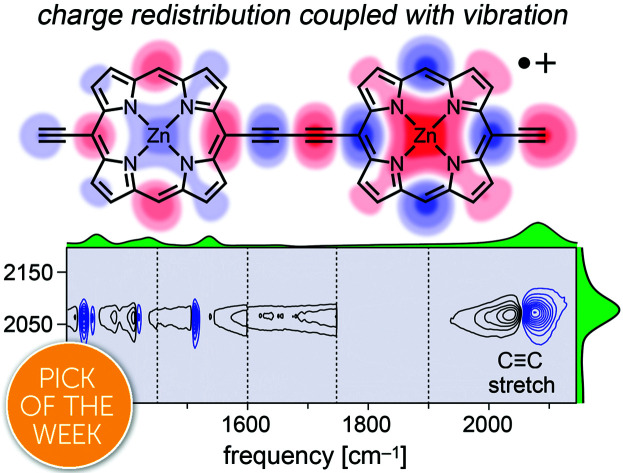

## Introduction

Electronic transitions are typically promoted by photons with energies greater than 10 000 cm^−1^ (*λ* < 1000 nm), and the associated electron density redistribution is faster than the response of the nuclei. On the other hand, low energy photons (500–3000 cm^−1^; *λ* ≈ 3000–20 000 nm) excite molecules to higher vibrational states, which are classically described by harmonic motions of nuclei. Electronic excitations generally have oscillator strengths 100–1000× greater than those of vibrational transitions and UV-vis-NIR absorption bands are far more intense than IR bands in most organic compounds, despite the fact that IR bands are significantly sharper.

**Fig. 1:**
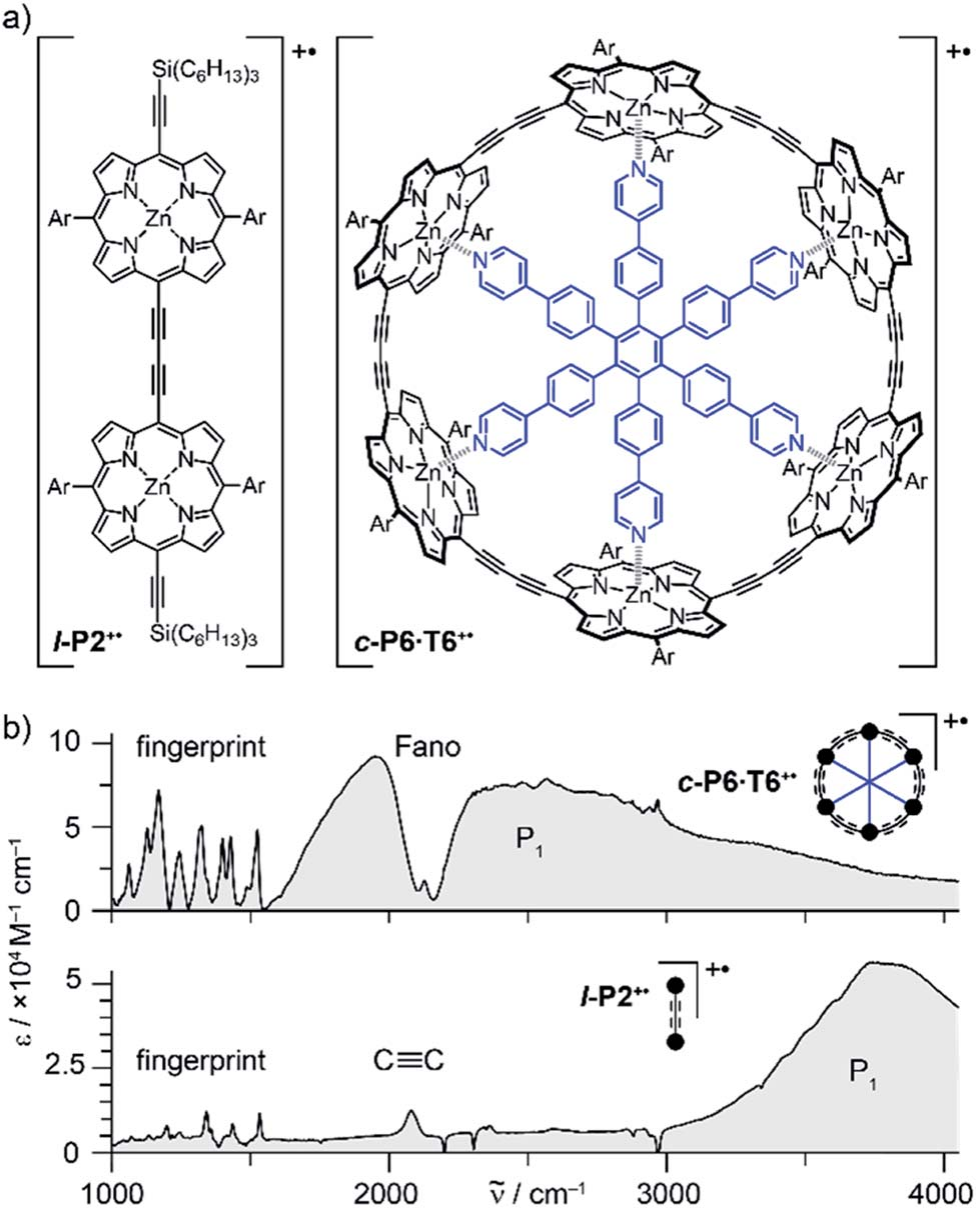
(a) Chemical structures of the porphyrin oligomer radical cations **l-P2+˙** and **c-P6·T6+˙**. Ar = 3,5-bis(trihexylsilyl)phenyl. (b) Their FTIR spectra recorded in CD_2_Cl_2_ showing the fingerprint region, the C≡C stretch band, the Fano anti-resonance (for **c-P6·T6+˙**) and the P_1_ polaron band.

Oxidation of a family of π-conjugated porphyrin molecular wires (Fig. 1a) has been shown to generate radical cations that exhibit IR vibrational transitions with intensities on the same order of magnitude as electronic transitions.^1–4^ The behavior of these species can be explained by treating the charge or ‘hole’ as a polaron, which is understood as a geometrical reorganization localized over 2–3 porphyrin units.^3^ The polaron exhibits a low energy electronic absorption band P_1_ in the region 1500–6000 cm^−1^ corresponding to a transition between the highest occupied molecular orbital (HOMO) and the singly occupied molecular orbital (SOMO).^2,5,6^ The IR bands of these cations (Fig. 1b) are much more intense than those of the neutral oligomers; they have molar attenuation coefficients *ε* ≈ 10–70 mM^−1^ cm^−1^, far beyond the intensities expected for vibrational transitions. Here we present a study of the origin of this huge IR band intensification in both linear and cyclic π-conjugated porphyrin oligomers.

**Fig. 2:**
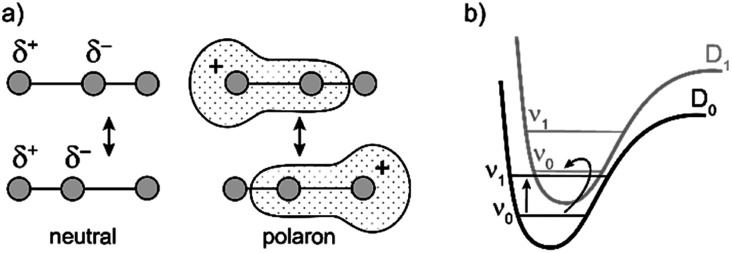
Schematic representation of two possible origins of IRAVs. (a) The polaron redistributes along the backbone in response to molecular vibrations. (b) The energies of electronic and vibrational transitions are comparable, leading to strong vibronic coupling.

Similarly intensified IR bands have been observed in the polarons of doped conjugated polymers,^7,8^ and mixed-valence coordination complexes,^9^ where they are known as ‘infrared active vibrations’ (IRAVs). This terminology arose because they were attributed to infrared-activation of Raman modes by electric fields.^4,7,10^ Their origin is attributed to two processes: coupling between charge redistribution and vibrational motion (Fig. 2a), or mixing between low-lying electronic and vibrational transitions (Fig. 2b).^8,9,11,12^ Either of these mechanisms could explain the IRAVs in doped butadiyne-linked porphyrin oligomers, especially as the energy of the formally electronic P_1_ transition is similar to those of the classically vibrational IRAV transitions (Fig. 1b).^3^

The radical cations of butadiyne-linked porphyrin oligomers are suitable systems to explore the behavior of IRAVs using advanced ultrafast spectroscopies including two-dimensional infrared spectroscopy (2D-IR) and time-resolved infrared spectroscopy (TRIR). Polarization-sensitive ultrafast spectroscopies^13^ have been used previously to explore IRAVs in doped conjugated polymers, and 2D electronic spectroscopy has been used to explore optically generated polaron pairs in conjugated polymers.^14^ The discrete molecular nature of our systems allows us to elucidate mechanistic insights into the behavior of polarons on a molecular wire.

2D-IR is a technique used to elucidate equilibrium structural dynamics, vibrational mode coupling and energy transfer in complex molecular structures.^15–18^ It offers simultaneous resolution of the pump and probe axes, as well as ultrafast time resolution (<1 ps), providing a snapshot of the coupling between vibrational modes at a given pump-probe delay time.^19^ Recording data at varying delay times between pump and probe pulses extends the utility of the technique, enabling analysis of chemical dynamics and kinetic decay processes. TRIR offers similar benefits to 2D-IR, at the expense of a resolved pump axis.

Previous work suggests the polaron (*i.e.* positive charge) in our systems is delocalized over 2–3 porphyrin units,^3^ and that the charge is fully delocalized in the dimer radical cation (Robin-Day class III).^3^ Consequently, we propose the linear dimer **l-P2+˙** as a model molecular polaron, and contrast its properties with those of the cyclic hexamer **c-P6·T6+˙** (Fig. 2a). The dimer radical cation **l-P2+˙** exhibits a broad P_1_ absorption band of electronic origin in the region >3000 cm^−1^; a band around 2050 cm^−1^ which is characteristic of the C≡C alkynyl stretch, observed as a discrete intensified vibrational-like absorption; and a series of IRAVs in the fingerprint region (1000–1600 cm^−1^).

The structurally well-defined C≡C vibrational stretch absorption in **l-P2+˙** is isolated in energy between the formally electronic P_1_ band and the IRAVs in the fingerprint region, providing an unambiguous handle with which to explore the behavior of the polaron and IRAVs. Conversely, **c-P6·T6+˙** shows a Fano-type anti-resonance at this energy, due to the overlap of the C≡C vibrational alkynyl stretch with the lower-energy P_1_ polaron electronic band (Fig. 2b).^3^ This overlap causes a cancellation of oscillator strengths due to destructive quantum interference between the electronic continuum and discrete vibrational transition.^4,6,20^

## Experimental

### Synthesis

The neutral porphyrin oligomers **l-P2** and **c-P6·T6** were synthesized as reported previously.^21,22^ Decoration of porphyrins with *meso*-3,5-bis(trihexylsilyl)-phenyl substituents improves solubility and prevents aggregation in the absence of pyridine (exclusion of which is essential to avoid unwanted reactions between pyridine and the radical cations).^3,23,24^

### Sample preparation for spectroscopic investigation

Experiments were performed in an optically transparent thin-layer electrochemical cell (OTTLE) fitted with 2 mm CaF_2_ windows;^25^ path length: *ca.* 0.2 mm; solvent: CD_2_Cl_2_ containing 0.1 M tetra-*n*-butylammonium hexafluorophosphate (Bu_4_NPF_6_) as supporting electrolyte. **l-P2+˙** and **c-P6·T6+˙** concentrations were adjusted to give a maximum optical density of 0.4–0.8 in the P_2_ bands (see Fig. S2†) by NIR spectroscopy (∼800–1000 nm), which is equivalent to optical densities of 0.2–0.5 in the fingerprint region, by adjusting the applied voltage with reference to the known oxidation potentials of the porphyrin radical cations (**l-P2+˙***E*_ox,1_ = 0.39 V, **c-P6·T6+˙***E*_ox,1_ = 0.20 V, both referenced to Fc/Fc^+^, see ESI† for further discussion on sample preparation).^3^ This corresponds to a concentration of monocation of ∼0.1 mM.

### UV-vis-NIR and IR spectroscopy

The FTIR and UV-vis-NIR spectra of **l-P2+˙** and **c-P6·T6+˙** were reported previously.^3^ FTIR spectra of **l-P2** and **c-P6·T6** were recorded using a FTIR Bruker Tensor 37 and a Shimadzu UV-1800 UV Spectrograph.

### Time-resolved and 2D-IR measurements

Time-resolved and 2D-IR spectra were collected using spectrometers at the STFC Rutherford Appleton Laboratory, as described in detail elsewhere.^19^ Ultrafast amplified lasers pump optical parametric amplifiers to generate the tunable ultrafast pump and probe pulses. Experiments were performed on both ULTRA, a titanium sapphire amplifier system with broadband, shorter pulse capability (10 kHz, >50 fs, >300 cm^−1^), and LIFEtime, a ytterbium laser system with narrower band, longer pulses (100 kHz, >180 fs, <200 cm^−1^). While the ULTRA system allows a broad IR spectrum to be probed, LIFEtime uses two IR probe beams, enabling the observation of two narrower separate spectral windows, denoted probe **a** and probe **b**. ULTRA's broader bandwidth also permits simultaneous pumping of a broader spectrum, which is important, for example, for simultaneously pumping the modes in the frequency range 1200–1600 cm^−1^. Where necessary, we note in each section which system (broadband ULTRA or the narrower-band LIFEtime) has been used. In practice, ULTRA allows the recording of larger pump/probe regions but requires longer acquisition times. Kinetics were extracted from 2D-IR LIFEtime and ULTRA TRIR data.

The pump-probe pulse time-delay is controlled by optical delay stages and the pump and probe beams are focused to <150 and <75 μm diameter, respectively, at the sample. The probe beams are collimated after the sample and delivered to MCT-array IR spectrometers, providing the probe frequency axis in the spectra.

The difference between the TRIR and 2D-IR experiments is that in 2D-IR, rather than modulating the pump pulse on–off with a chopper, the pump pulses pass through an acousto-optic pulse shaper.^18^ This device generates a pulse pair with varying delay, mimicking a rapid scanning interferometer, with phase cycling capability. Collected probe spectra at a range of interpulse delays are Fourier-transformed through this parameter to give the frequency-resolved pump spectrum axis in the 2D-IR spectra.

All samples measured in this set-up had optical densities of 0.2–0.5 in P_1_ and fingerprint regions, to allow qualitative comparison between data sets, *in lieu* of known exact concentrations of monocation species.

Polarization experiments were conducted by rotating the pump relative to the probe by a *λ*/2 plate, measuring angles 0°, 54.7° and 90° at each time delay. Randomizing time delays, and recording concurrent spectra of parallel and perpendicular pump-probe orientations at a given delay time, allowed the calculation of anisotropies while minimizing the effect of fluctuations in the concentration of the electrochemically generated radical cations.

The polarization dependent response of the detectors was tested using a W(CO)_6_ standard, and compared to anisotropy values reported in the literature (Fig. S24†).^26^

## Results

### 2D-IR spectroscopy

#### (1) Fingerprint region (1200–1600 cm^−1^)

**Fig. 3:**
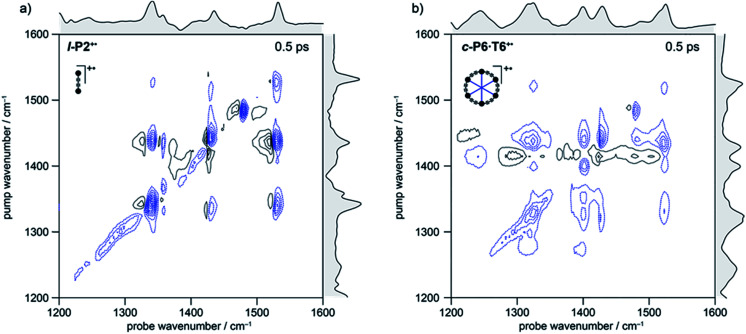
2D-IR spectra of **l-P2+˙** (a) and **c-P6·T6+˙** (b) at 500 fs delay in the fingerprint region. Black solid and blue dashed contour lines correspond to positive (ESA) and negative (GSB) signals, respectively. Contours near zero are hidden for clarity. Spectra were recorded using a broadband pump centered at ∼1325 cm^−1^ (FWHM ∼150 cm^−1^). Optical densities of all samples were ∼0.2–0.4 in the fingerprint region. Linear FTIR spectra are shown in gray along each axis.

Broadband ULTRA was used to record 2D-IR spectra of the fingerprint region (1200–1600 cm^−1^) for a range of time delays (400–1500 fs). The 2D-IR spectra of the oxidized species **l-P2+˙** and **c-P6·T6+˙** (at 500 fs, Fig. 3a and b) show significant coupling between the majority of the modes as evidenced by the appearance of off-diagonal features at early times.

In the spectrum of **l-P2+˙** the on-diagonal features appear as pairs of signals, consisting of negative ground-state bleach signals (GSB, blue) with associated positive excited-state absorptions (ESA, black) corresponding to ν_2_ ← ν_1_ transitions at lower energy. The ESAs are red-shifted from the GSB by the vibrational anharmonicity.

The spectrum of **c-P6·T6+˙** is similar to that of **l-P2+˙** showing significant coupling between all IRAV modes in the frequency range 1300–1600 cm^−1^. A major difference in intensity is observed between the GSB and ESA signals. In **c-P6·T6+˙**, the GSBs dominate the spectrum with little evidence of ESA at early times, whereas in **l-P2+˙** the ESA signals are more pronounced, albeit still weaker than corresponding GSB. At later times the GSB/ESA pair are of similar intensities (Fig. S7†). The GSB of **c-P6·T6+˙** are expected to be more intense due to the quadratic dependence of signal intensities on the molar absorption coefficient.^18^ The on-diagonal peak at ∼1480 cm^−1^ appearing in both monocation spectra (Fig. 3) results from residual neutral species, which show a strong on-diagonal peak at this frequency (Fig. S6†).

#### (2) Triple-bond stretch region (1300–2150 cm^−1^)

**Fig. 4:**
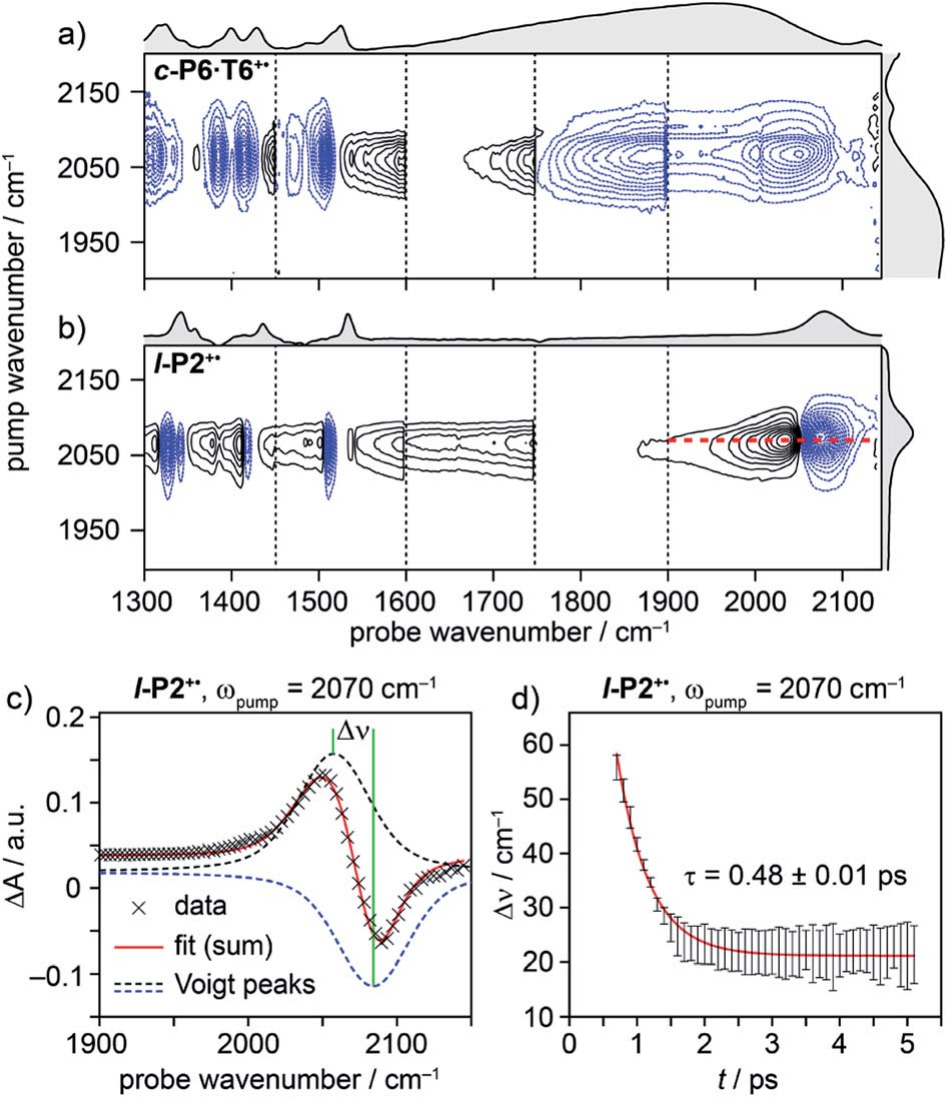
2D-IR spectra of **c-P6·T6+˙** (a) and **l-P2+˙** (b) at 400 fs delay, with pump (FWHM ∼80 cm^−1^) centered at 2080 cm^−1^. Black solid and blue dashed contour lines correspond to positive (ESA) and negative (GSB) signals, respectively. Vertical dashed lines correspond to different experimental spectra, where probe **a** was changed to cover region 1300 cm^−1^ to 1900 cm^−1^ and probe **b** was centered in the 1900–2150 cm^−1^ region for each experiment (see Fig. S14–S23† for time resolution of each probe window). Probe windows in 1300–1900 cm^−1^ were stitched together by normalizing all probe **a** spectra to probe. **b** (c) fit of on-diagonal slice for **l-P2+˙** (red line in (b)) using the sum of two Voigt. (d) The peak–peak distance for each time delay, calculated from the fit in (c).

The IRAV band centered at 2080 cm^−1^ in **l-P2+˙** is strong (∼20 mM^−1^ cm^−1^) and easily assigned to the C≡C alkynyl stretch (Fig. 1b). The narrowness, isolation and well-characterized nature of this band make it an ideal handle with which to explore the structure–property relationships of the IRAVs. Consequently, 2D-IR experiments were undertaken using the narrower pump bandwidth (full-width half-maximum, FWHM ∼ 80 cm^−1^) of the LIFEtime instrument centered on the C≡C stretch (*ω*_pump_ = 2080 cm^−1^, Fig. 4).

A full population time delay series was recorded for probe regions 1300 to 2150 cm^−1^, taking advantage of the short acquisition times of LIFEtime.^19^ In these experiments, a narrowband pump was centered at 2050–2100 cm^−1^, in direct resonance with the C≡C stretch of **l-P2+˙** and with the Fano-type anti-resonance in the P_1_ band of **c-P6·T6+˙**. The molecular responses were probed by selecting two spectral windows: probe **a** and probe **b**. Probe **a** was set to one of four spectral regions centered at ∼1370 cm^−1^, 1520 cm^−1^, 1650 cm^−1^ or 1800 cm^−1^. Probe **b** was set to the on-diagonal region centered at 2025 cm^−1^, giving a shared spectral window between each experiment and allowing normalization. In the resulting 2D-IR spectra (Fig. 4a and b), off-diagonal signals appear immediately (within 200 fs) for all the IRAVs in the FTIR spectra. Each IRAV probed below 1600 cm^−1^ appears as a GSB with associated ESA as a result of vibrational mode anharmonicity. The GSB signals (blue) are much stronger at earlier times compared to the anharmonic ESA signals (black), commensurate with the 2D-IR spectra shown in Fig. 3.

One major difference between the two species is the absence of a positive ESA signal in the on-diagonal region of the **c-P6·T6+˙** spectra at early times. We observe a broad positive ESA band that extends across the whole spectrum, which appears to be primarily electronic (like the P_1_ band). The absence of significant ESA signals for **c-P6·T6+˙** indicates that the IRAV amplification effect is suppressed in the excited polaron. In **l-P2+˙**, the GSB and ESA are well resolved as a result of the discrete C≡C stretch vibrational mode, although there is still a broad positive ESA feature extending across the whole fingerprint region. The presence of this feature, even in the absence of direct P_1_ pumping, suggests some degree of vibronic coupling in **l-P2+˙**, as confirmed by the observation of the coupling of P_1_ to all of the IRAVs in TRIR, as discussed below.

The evolution of the 2D-IR signals over time showed little change in the peak positions and shapes, with the exception of the **l-P2+˙** on-diagonal ESA/GSB pair at *ω*_pump_ = 2070 cm^−1^ and *ω*_probe_ = 2045/2078 cm^−1^ which appears to shift to higher frequencies within about 1 ps. To explore this time-dependence of the peak position, a sum of two Voigt profiles was fitted to the slice at *ω*_pump_ = 2070 cm^−1^ for the **l-P2+˙** on-diagonal C≡C stretch (Fig. 4c), because Voigt functions generally provide more accurate fits to IR data than purely Lorentzian or Gaussian profiles.^27^ The GSB Voigt was fixed to a FWHM of 41.3 cm^−1^ centered at 2084 cm^−1^ (values chosen from initial free fits at later times). The ESA Voigt was allowed to vary in both position and width. The Voigt profile peak positions for the ESA, ν_2_ ← ν_1,_ shift to higher frequency over time, resulting in a decrease in the apparent anharmonicity Δ*ν* from 60 cm^−1^ to 20 cm^−1^ (Fig. 4d). This blue shift of ∼40 cm^−1^ follows a mono-exponential decay with a lifetime of 0.48 ± 0.01 ps. The FWHM of the ESA stays fairly consistent over time around 50–55 cm^−1^. It is not clear whether this evolution reflects a true decrease in anharmonicity over time, or the decay of overlapping ESA bands.

### Kinetic analysis

The time-resolved nature of the 2D-IR data presented in Fig. 4 allows the decay of the GSB and ESA to be fitted to mono, bi or tri-exponential functions, convoluted with the instrument response function (IRF), providing information on the lifetimes of vibrational/electronic excited-states.^28^ These functions are hereafter referred to as mono-, bi- or tri-exponential, for simplicity. The evolution of the spectral features is complicated by large initial GSBs, multiple recovery processes and crowded spectra, especially at early times (time-resolved surface plots are presented in Fig. S14–S23†). The decay profiles are categorized into four types: (1) on-diagonal GSB, (2) off-diagonal GSB, (3) ESA peaks (anharmonic bands of the GSBs) and (4) the broad ESA stretching across the whole spectrum (most visible around 1550–1750 cm^−1^). In order to describe the salient features of the spectra along with their time dependence as simply as possible, we decided to fit the observed peaks to as few shared time constants as possible. The fitting process is described in detail in the ESI (see eqn (S1), Fig. S9, S10, Tables S3 and S4†).

We determined that the kinetic data for each species (**l-P2+˙** or **c-P6·T6+˙**) can be modeled in terms of three decay times, *τ*_1_, *τ*_2_ and *τ*_3_. Here we summarize the features of these decay processes and their associated states:

**(a)** ***τ***_**1**_ **: intramolecular energy redistribution.** This is a very fast process (**l-P2+˙**: *τ*_1_ = 0.15 ± 0.02 ps; **c-P6·T6+˙**: *τ*_1_ = 0.29 ± 0.01 ps) and it is mainly observed through the decay of the GSB (on-diagonal for **l-P2+˙**; both on- and off-diagonal for **c-P6·T6+˙**). It sits within the instrument response, but the good fit to the decay profile at early times for every on-diagonal GSB and the agreement with previously reported values of the instrument response for this spectrometer^19^ (*σ* ∼ 200 fs) enable us to confidently detect this fast decay component. *τ*_1_ appears as a rise-time for the ESA signals that decay with time-constants *τ*_2_ and *τ*_3_ (Fig. S11–S13†). This process is consistent with intramolecular vibrational energy redistribution (IVR), as described by Rubtsov and coworkers in their work on acetylproline-NH_2_ and relaxation-assisted 2D-IR spectroscopy (RA-2D-IR).^29–31^

**(b)** ***τ***_**2**_ **: relaxation from an “electronic” excited state** (**l-P2+˙**: *τ*_2_ = 0.92 ± 0.01 ps; **c-P6·T6+˙**: *τ*_2_ = 0.74 ± 0.07 ps). This process is mainly observed through the decay of the extremely broad featureless ESA band extending from 1300–2000 cm^−1^; in **l-P2+˙**, this decay is also detected as a component in the GSB. The resemblance of this ESA to the P_1_ absorption band suggest that this decay originates from a nominally electronic excited state that lacks the IRAVs of the P_1_ ground state.

**(c)** ***τ***_**3**_ **: relaxation from a “vibrational” excited state** (**l-P2+˙**: *τ*_3_ = 5.7 ± 0.1 ps; **c-P6·T6+˙**: *τ*_3_ = 4.1 ± 0.3 ps). This component appears in most of the signals and it is the dominant relaxation process for the sharper ESA signals. It resembles a normal vibrational relaxation process and it originates from a state populated by IVR that shows ESA/GSB pairs of similar intensity.

### TRIR spectroscopy and anisotropy

**Fig. 5:**
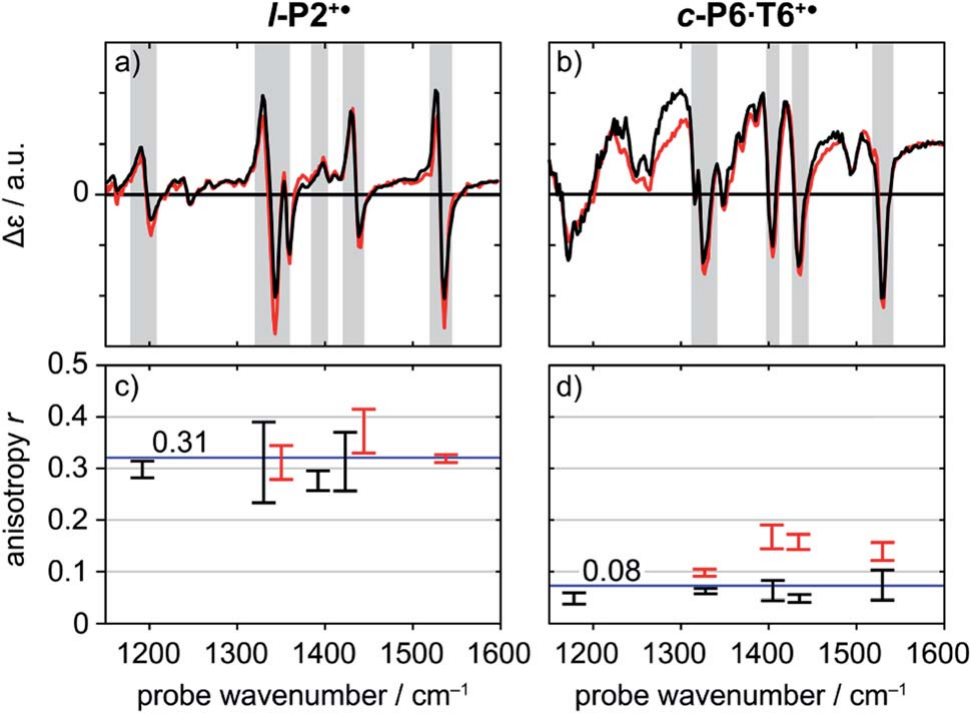
Normalized TRIR spectra of **l-P2+˙** (a) and **c-P6·T6+˙** (b) with pump centered on C≡C stretch mode (2100 cm^−1^) (black) and on P_1_ polaron band (3250 cm^−1^ for **l-P2+˙**, 2500 cm^−1^ for **c-P6·T6+˙**) (red) with probe delay 0.8 ps. Anisotropy values of the selected TRIR regions (greyed area in the spectra) of **l-P2+˙** (c) and **c-P6·T6+˙** (d) are plotted for both pump positions, with error bars indicating standard error.

A series of TRIR spectra of **l-P2+˙** and **c-P6·T6+˙** were recorded by pumping the regions around 2100 cm^−1^, 2500 cm^−1^ or 3250 cm^−1^ and probing 2100 cm^−1^ or the fingerprint region. In the fingerprint region, the TRIR spectra of both **l-P2+˙** and **c-P6·T6+˙** are similar for times >1 ps, irrespective of whether the pump excites the C≡C stretch (in **l-P2+˙**), the Fano-type anti-resonance (in **c-P6·T6+˙**) or P_1_ polaron band (both) (Fig. 5a and b). Higher frequency pumps in general lead to more intense initial bleach features, disappearing rapidly within 1 ps. The presence of such features is less pronounced when the pump is applied at 2100 cm^−1^.

Polarization experiments were performed to study the relative orientations of the IRAV modes. Experiments of this type are often used to establish the relative orientation between pumped and probed processes.^32,33^ The vibrational anisotropy *r* was calculated using eqn (1).^13,33^ In isotropic solution, the anisotropy is expected to be in the range −0.2 < *r* < 0.4, considering extremes with the transition dipole moment of induced absorption perpendicular or parallel to the excitation, respectively.1
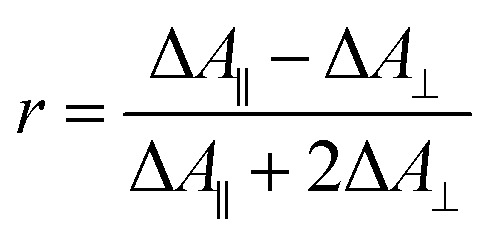


TRIR spectra using the magic angle (54.7°) orientation Δ*A*_mag_, were acquired and served as a check of the experimental setup. Measurements with this orientation obeyed the expected relation (eqn (2)) to data obtained from parallel Δ*A*_∥_ and perpendicular Δ*A*_⊥_ setup (Fig. S26†).2
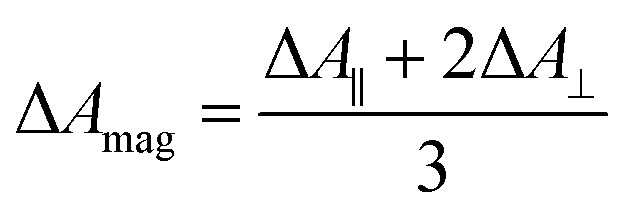


Since **l-P2+˙** and **c-P6·T6+˙** are large molecules with molecular weight of 4.0 kDa and 11 kDa, respectively, no anisotropy decay due to rotational reorientation is expected on the experimental time scale.

**Fig. 6:**
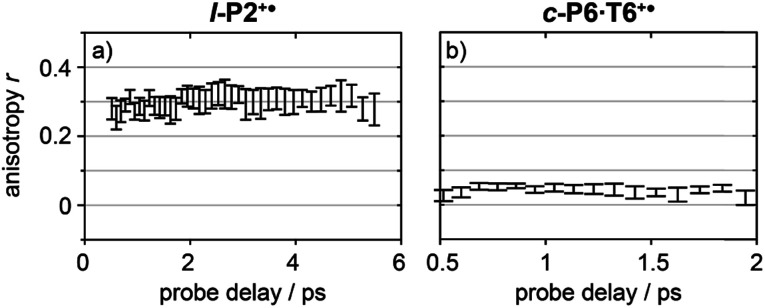
Average anisotropy of all analyzed signals (greyed areas in Fig. 5a and b) of **l-P2+˙** (a) and **c-P6·T6+˙** (b) pumped both at 2100 cm^−1^ and 3250/2500 cm^−1^ as a function of probe time delay.

Anisotropy of all TRIR signals in the fingerprint region against the time for both **l-P2+˙** and **c-P6·T6+˙** are plotted in Fig. 6a and b, respectively, showing no significant change over the time. Due to instrument response effects at early times, the anisotropy is analyzed at probe delays >0.5 ps.

Investigation of all probe regions with a signal-to-noise above a selected threshold (Fig. S27–S32†) showed that the anisotropy values for each ESA/GSB pair have similar values in the range *r* = 0.28–0.38 for **l-P2+˙** and *r* = 0.03–0.12 for **c-P6·T6+˙** (Fig. 5c and d).

The fact that the anisotropies are independent of pump frequency allows the values to be averaged across the whole spectrum for each system, resulting in anisotropies of *r* = 0.31 ± 0.07 for **l-P2+˙** and *r* = 0.08 ± 0.04 for **c-P6·T6+˙**, both these average values are constant over time, as seen in Fig. 6.

### Excited states of neutral oligomers

**Fig. 7:**
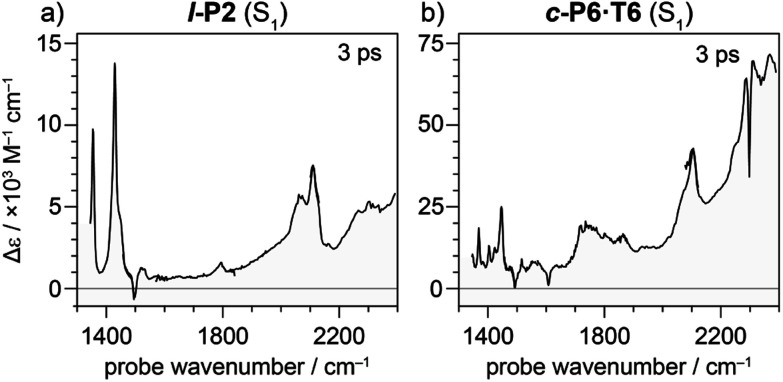
TRIR spectrum at 3 ps delay of neutral molecules (a) **l-P2** and (b) **c-P6·T6** excited to S_1_ excited state with visible light (714 nm and 772 nm, respectively). Strong ESA and weak or absent GSB is observed suggesting the presence of IRAVs in the S_1_ excited states of neutral molecules.

Electronic excitation of neutral porphyrin oligomers leads to population of the S_1_ excited state. Probing the IR region of S_1_ excited states reveals absorption spectra similar to those of the radical cations (Fig. 7), implying that the photoexcited state has a similar electronic structure to the polaron. A similar congruence between excited states and polaronic states has been observed in the excited states of polymer blends, colloidal quantum dots and perovskites.^34,35^ There appears to be a broad electronic absorption band at 2000–2400 cm^−1^, together with an intensified C≡C stretch signal at 2050–2100 cm^−1^, and other IRAVs in the finger print region at 1300–1600 cm^−1^. The GSBs are very weak, since the ground state IR absorption is weak in the neutral molecules. In contrast, the ESAs are relatively strong, consistent with the presence of IRAVs in the S_1_ excited state of the neutral porphyrin oligomer. Similar intense IR transitions have been observed in the neutral excited states of donor–acceptor systems.^36^

### DFT calculations

**Fig. 8:**
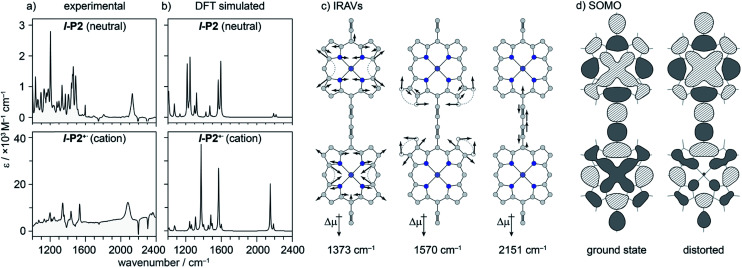
(a) Experimental and (b) DFT simulated IR spectra of **l-P2** and **l-P2+˙**. DFT (LC-ωPBE/6-31G*) predicts an increase in relative intensity of some vibrational absorptions by a factor of ∼50. (c) Three most intensified vibrational modes of **l-P2+˙** with displacement vectors and dipole moment change. (d) Calculated HOMO(/SOMO) of vibrationally relaxed and distorted 1570 cm^−1^ mode (∼10 of the classical turning point) of **l-P2+˙**, showing SOMO density redistribution away from contracted ring.

We sought to assign the observed IRAVs by carrying out DFT calculations using the range-separated LC-ωPBE functional^37–39^ and the 6-31G* basis set (which method was empirically found to correlate well with experimental observations in porphyrin oligomers monocations)^3^ using the Gaussian09 software.^40^ Models of **l-P2+˙** and **c-P6·T6+˙** (without solubilizing groups in **l-P2+˙** and without solubilizing groups and template in **c-P6·T6+˙**) were built and geometries were minimized with charge 1+ and doublet multiplicity. Frequency calculations on the neutral and cationic models correctly predicted that several IR absorptions are intensified by a factor of 50–100 in the cations, including the C≡C bond stretch and several bands in the fingerprint region (Fig. 8a and b).

The dipole derivatives of the modes with the most intensified absorptions show 100% alignment with the C≡C axis along the length of the **l-P2+˙** molecule (Fig. 8c, for more details see ESI Section 5†). The DFT calculated spectra do not exactly match the experimental spectra suggesting a dissimilarity between the modes in neutral and oxidized species as seen in doped conjugated polymers.^41^

## Discussion

The 2D-IR spectra reveal strong coupling in the radical cations **l-P2+˙** and **c-P6·T6+˙**. All of the IRAVs in the fingerprint region are coupled to the C≡C stretch vibration and to the P_1_ polaron band, as evidenced by the appearance of 2D-IR off-diagonal signals at early times (Fig. 4 and S14–S23†). The TRIR show that pumping the C≡C stretch or the higher energy polaron P_1_ bands yields identical TRIR spectra. The spectra do not exhibit significant differences in kinetic response or in anisotropy, suggesting that the species generated by excitation of a classically vibrational mode (C≡C stretch) or a classically electronic transition (the polaron band) are identical, which confirms that the polaron absorption and vibrations must be strongly coupled. This result confirms that the Born–Oppenheimer approximation is an unsuitable framework from which to consider the nature of substantially delocalized polarons.

The kinetic evolutions of the 2D-IR spectra fit a model with just three decay times: *τ*_1_ for IVR, *τ*_2_ for relaxation from an “electronic” excited state and *τ*_3_ for relaxation from a “vibrational” excited state. This “electronic” excited state is unusual: it exhibits a featureless ESA band, extending from 1300–2000 cm^−1^, with no apparent IRAVs in **c-P6·T6+˙**. One explanation for this lack of IRAVs might be that they are suppressed by the same Fano-type anti-resonance that occurs with overlap of electronic and vibrational transitions in the ground state (Fig. 1).^3^

The TRIR anisotropy measurements show similar anisotropies for all of the signals in the fingerprint regions of **l-P2+˙** and **c-P6·T6+˙** (Fig. 5 and 6). The anisotropy value of 0.31 found for **l-P2+˙** is not far from the theoretical limit of 0.4 expected for a simple linear transition dipole, while the value of 0.08 found for **c-P6·T6+˙** is near the limit of 0.1 expected for 2D delocalization.^22^**l-P2+˙** is a linear molecule and its high TRIR anisotropy demonstrates that the intensified vibrations can be assigned to transitions parallel with the length of the molecule, *i.e.* parallel to the P_1_ transition and C≡C stretch vibrations.

At first sight, the low anisotropy measured for **c-P6·T6+˙**, which implies that the vibrationally-excited polaron is delocalized around the whole nanoring, is surprising because previous results indicated that the polaron is only delocalized over 2–3 porphyrin units. Together, these results imply that, on IR-excitation, the polaron migrates around the circumference of the nanoring faster than the TRIR measurement, *i.e.* within 0.5 ps, at 298 K. In contrast, polaron migration is slow on the timescale of linear electronic and vibrational measurements (∼fs) at 298 K, and on the EPR timescale (∼100 ns) at 80 K.^3^

The time-independence of the anisotropies supports the assignment of IRAVs to being within the porphyrin framework, as this assignment fixes the relative orientations of the pumped and probed transitions. This contrasts with systems that have coupled bonds capable of rotating in relation to each other, which in turn causes a change in anisotropy over time.^42^ The apparent observation of IVR effects in **l-P2+˙** is also consistent with the assignment of the IRAV modes to transitions along the C≡C axis of the molecule, as IVR is known to occur efficiently between modes having strong spatial mode overlap (*i.e.* between modes that share vibrating atoms).^31^

The evidence presented above implies that all the IRAV modes in **l-P2+˙** are aligned along the long axis of the butadiyne-containing skeleton, *i.e.* along the porphyrin–porphyrin axis and the C≡C stretch direction. This, together with the assignment of fingerprint vibrations by DFT, is consistent with the polaron redistribution mechanism as proposed by Zamadar *et al.* (Fig. 2a) and the explanation for previously observed infrared ‘marker bands’ in porphyrin monomers where intensified infrared transitions are assigned to contracting and expanding breathing modes with transition dipole moments passing through opposed *meso* positions.^12,43^ The intensification in those monomers arises as the contraction of one side of the porphyrin results in charge redistribution to minimize anti-bonding interactions on the contracted side. DFT calculations of **l-P2+˙** support the assignment of the intensified vibrations to similar contracting and expanding modes, and the calculated dipole derivatives of the most intensified vibrations are oriented along the long axis of **l-P2+˙** (Fig. 8).

The coupling between vibrational modes and charge redistribution can be appreciated by calculating the distribution of the SOMO at distorted geometries, at which the molecule is mechanically compressed/expanded during a vibration to the extent of 10 times the energy of the first vibrational transition (Fig. 8d). The mechanism of vibronic coupling can thus be explained in the case of **l-P2+˙**: as one porphyrin ring contracts, the anti-bonding orbital lobes are brought closer together. In response to this, the mobile polaron redistributes to the expanded ring, causing a large change in the dipole moment. The electron density in these systems is effectively moving from one porphyrin ring to another, in a way analogous to an electronic transition, providing an explanation for the exceptionally high intensities observed for these vibrations. This mechanism relies on the polaron being mobile along the oligomer backbone and redistributing in response to vibrations. It follows that the polaron must be mobile in **c-P6·T6+˙**, as confirmed by the observed low anisotropy.

The delocalization length of polarons in conjugated polymers tends to be reduced by strong electron–phonon coupling.^11,44,45^ The assignment of the IRAVs (by definition strongly coupled phonon modes) to those of breathing modes across two porphyrin rings is consistent with the previously reported delocalization length of 2–3 porphyrin units in the radical cations of butadiyne linked porphyrin oligomers.^3^

DFT calculations predict a huge intensification of several vibrational bands, but they fail to reproduce the observed pattern of activated vibrations in the fingerprint region, even for the simpler system of **l-P2+˙**. DFT predicts only two IRAVs in the window 1100–1800 cm^−1^ compared to at least five modes observed in the experimental IR spectrum.

## Conclusions

Time-resolved IR spectroscopies have been used to probe the origins of IRAVs in the radical cations of porphyrin oligomers. 2D-IR studies of two compounds, **l-P2+˙** and **c-P6·T6+˙**, show that vibrational coupling occurs at early times between all of the IRAVs and with the P_1_ polaron bands. The similarity between spectra arising from pumping in the classically vibrational C≡C stretch and those arising from pumping the P_1_ polaron bands demonstrates the presence of strong vibronic coupling between the P_1_ band and IRAV modes. This result, in combination with results from DFT calculations and polarization-dependent TRIR experiments, suggest the IRAV modes have a substantial vector component in the direction of the C≡C backbone of **l-P2+˙**. This assignment is consistent with the idea that IRAVs originate from polaron redistribution in response to, and on the time scale of, the vibration, which amplifies the transition dipole moment of the vibrational mode.

Coupling between vibrational modes and charge transport is frequently an important factor determining the rate of electron transfer through molecular wires in single-molecule electronic devices.^46–48^ If we assume that the vibrational modes in **l-P2+˙** are similar to those in **c-P6·T6+˙**, then the anisotropy of ∼0.08 recorded for **c-P6·T6+˙** suggests that, in the vibronic excited state, the polaron is mobile along the length of the molecular backbone, consistent with previous discussions of polarons in polymer chains.^11^

## Author contributions

WJK, MJ and MDP collected and analyzed the 2D-IR and TRIR data. MDP synthesized the **c-P6·T6**. GMG, IVS, PMD and MT helped to design and implement the 2D-IR and TRIR experiments. MDP, AWP and HLA initiated the project. MJ and MDP carried out the computational work. All authors contributed towards interpretation of the data. WJK and MJ wrote the manuscript, with contributions from all authors.

## Conflicts of interest

There are no conflicts to declare.

## Supplementary Material

SC-011-C9SC05717J-s001

SC-011-C9SC05717J-s002
