# Supplementary material for: Mechanisms of IR amplification in radical cation polarons
Source: Chem Sci. 2020 Jan 22;11(8):2112–20. doi: 10.1039/c9sc05717j (PMC8150116; doi:10.1039/c9sc05717j)
Supplement: SC-011-C9SC05717J-s001 [file SC-011-C9SC05717J-s001.pdf]

Electronic Supplementary Material (ESI) for Chemical Science.
This journal is © The Royal Society of Chemistry 2020

Electronic Supplementary Information for

Mechanism of IR Amplification in Radical Cation Polarons

William J. Kendrick,[a]† Michael Jirásek,[a]† Martin D. Peeks,[a] Gregory M. Greetham,[b] Igor V. Sazanovich,[b] Paul M. Donaldson,[b] Michael Towrie,[b] Anthony W. Parker[b]* and Harry L. Anderson[a]*

[a]Department of Chemistry, University of Oxford, Chemistry Research Laboratory, Oxford OX1 3TA, UK
[b]Central Laser Facility, Research Complex at Harwell, Science and Technology Facilities Council, Didcot OX11 0QX, UK

† These authors contributed equally

E-mail: harry.anderson@chem.ox.ac.uk and tony.parker@stfc.ac.uk

Contents

1 Experimental S2
1.1 Synthesis of porphyrin molecular wires S2
1.2 FTIR and UV-vis-NIR Instrumentation S2
1.3 Measurements of neutral compounds S2
1.4 Preparation and ultrafast measurements of monocations S2
1.5 UV-vis-NIR, FTIR and Raman spectra of neutral and monocation species S4
2 2D-IR S7
2.1 Measurement of Neutral (***l*-P2** and ***c*-P6·T6**) using ULTRA S7
2.2 Measurement of ***l*-P2$^{+•}$** and ***c*-P6·T6$^{+•}$** at later times (1.5 ps) using ULTRA S8
2.3 Fitting of *l*-P2$^{+•}$ LIFEtime 2D-IR on-diagonal slices (ω_{pump} = 2070 cm^{-1}) S8
2.4 Fitting of LIFEtime 2D-IR kinetic traces S9
2.4.1 ***l*-P2$^{+•}$** S11
2.4.2 ***c*-P6·T6$^{+•}$** S13
2.4.3 Grow-in fitting S14
2.5 Slices of time-resolved LIFEtime 2D-IR data S17
2.5.1 ***l*-P2$^{+•}$** S18
2.5.2 ***c*-P6·T6$^{+•}$** S20
3 TRIR S23
3.1 Polarization-sensitive TRIR S23
3.2 TRIR anisotropy analysis S24
3.3 Kinetic analyses of TRIR spectra S28
3.3.1 ***l*-P2$^{+•}$** TRIR kinetic analysis S28
3.3.2 ***c*-P6·T6$^{+•}$** TRIR kinetic analysis S35
4 Neutral molecules excited state absorption S41
5 DFT Calculations S42
6 References S47

1 Experimental

1.1 Synthesis of porphyrin molecular wires

The zinc porphyrin oligomers ***l*-P2** and ***c*-P6·T6** were synthesized according to previously published procedures.[1–3] All manipulations of air- or water-sensitive compounds were performed using standard Schlenk technique. Commercially available reagents and solvents were used without further purification.

1.2 FTIR and UV-vis-NIR Instrumentation

FTIR and UV-vis-NIR of all compounds were recorded using a FTIR Bruker Tensor 37 and Shimadzu UV-1800 UV Spectrograph respectively. The optical path was purged with dry N_2.

1.3 Measurements of neutral compounds

FTIR and 2D-IR (using ULTRA, **Fig. S6**) of neutral compounds ***l*-P2** and ***c*-P6·T6** were performed in Harrick cells fitted with 2 mm CaF_2 windows; path length: 0.15 mm; solvent: CD_2Cl_2. Typical optical densities were 0.2–0.5 in the fingerprint IR region, which corresponds to a concentration of approximately 5–10 mM.

1.4 Preparation and ultrafast measurements of monocations

***l*-P2**$^{+\bullet}$ and ***c*-P6·T6**$^{+\bullet}$ solutions were generated spectroelectrochemically in an optically transparent thin layer electrochemistry (OTTLE) cell comprising platinum gauze working and counter electrodes and a Ag/AgCl quasireference electrode.[4] This OTTLE cell was purchased from Prof. Frantisek Hartl, University of Reading, UK. The cell was masked using electrical tape and aluminium foil such that the only clear optical path went through the centre of the working electrode. The electrolyte solution was 0.1 M tetra-*n*-butylammonium hexafluorophosphate (Bu_4NPF_6 or TBAP) in CD_2Cl_2. The cell was filled with neutral solutions of ***l*-P2** and ***c*-P6·T6** with concentrations of ca. 2 mg/mL in CD_2Cl_2, or ~0.5 and 0.2 mM respectively. Monocations were prepared by holding a constant potential in the OTTLE cell, using a chronoamperometry experiment. The potential was set with reference to the known potentials of the monocations as recorded using square wave voltammetry with Fc/Fc^+ as an internal reference (**Fig. S1**, c.a. 0.39 V for ***l*-P2** and ***c*-P6·T6** 0.2 V).[5] In the OTTLE cell, these voltages were in the range 400–800 mV, the difference as a result of the known Ag/AgCl vs Fc/Fc+ value of + 0.49 V in CH_2Cl_2-Bu_4NPF_6 (0.1 M).[6]

The square wave volammograms show a 1+/2+ wave close to the 0/1+ wave. To avoid contamination of the desired monocation signal with potentially IR-active 2+ states (as in case of the ***c*-P6·T6**), potentials were adjusted ad hoc for each sample within the regions on the profile shown in **Fig. S1** (dotted lines) to generate mixtures of the neutral (which is relatively silent in the IR) and optically active monocation states. The compositions of the oxidized solutions were analyzed using UV-vis-NIR spectroscopy, and ratios of the porphyrin Q bands of the neutral and P_2 bands in the monocations (see **Fig. S2**) were used to estimate ratio of neutral:monocation.[7]

FTIR spectra of ***l*-P2**$^{+\bullet}$ and ***c*-P6·T6**$^{+\bullet}$ were collected by first recording a background measurement of the OTTLE cell at 0 V potential (i.e. neutral in 0.1 M TBAP/CD_2Cl_2). The monocations were then generated as described above without moving or adjusting the cell and the FTIR measured.

In general, we found the samples to be stable for up to ~45 min–1 hour (while keeping constant applied potential), but often the concentrations of monocation drifted even within this time frame. For LIFEtime measurements, a real-time IR readout allowed for adjustment of the applied potential during the measurement to keep the intensity of the IR absorptions (and thus concentrations) constant. For ULTRA TRIR measurements, a UV-vis-NIR absorption spectrum of the OTTLE cell was recorded before and after measurements, to account for potential drifts in the monocation concentrations (see **Fig. S5** for a representative example). Where the concentration of monocation changed significantly (± ~30%), or where there was any evidence of 2+ states, the cell was refilled with fresh solution and the experiment repeated. The effects of small drifts in concentration on kinetic profiles were minimized by randomly ordering the experimental time delays where the instrument allowed. For ULTRA 2D-IR measurements, the samples were found to drift back to neutral due to the significantly longer acquisition times. However, due to the 40× smaller signals in the neutral species and because only one time delay was recorded, the spectra averaged over the duration of the measurement were deemed to be representative of the monocation species (neutral control measurements are shown in **Fig. S6**). Due to these sample stability issues, we cannot quantitatively determine monocation concentrations for the ultrafast spectroscopy experiments.

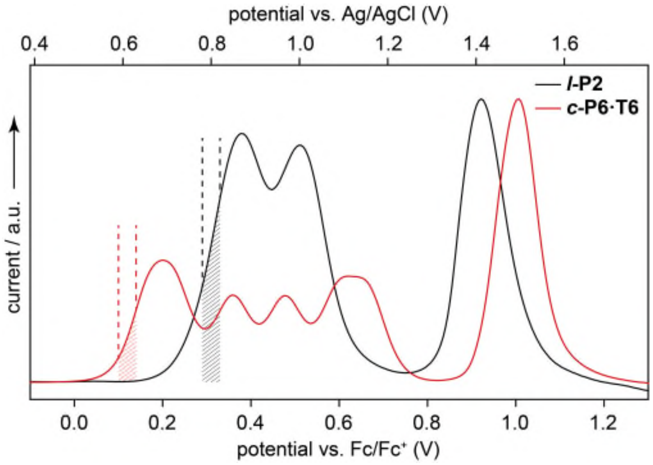

Fig. S1. Experimental square wave voltammogram (CH_2Cl_2/0.1 M Bu_4NPF_6) of porphyrin oligomers, using a glassy carbon working electrode; platinum wire counter electrode and Ag/AgCl non-aqeuous quasireference electrode. Fc/Fc+ was used as an internal reference. 5 mV step potential, 10–50 mV modulation amplitude and 5 Hz frequency was used. Conversion to potential vs Ag/AgCl (+0.49 V)[6] shown for comparison. Shaded areas represent potentials used during ultrafast spectroscopy experiments.

1.5 UV-vis-NIR, FTIR and Raman spectra of neutral and monocation species

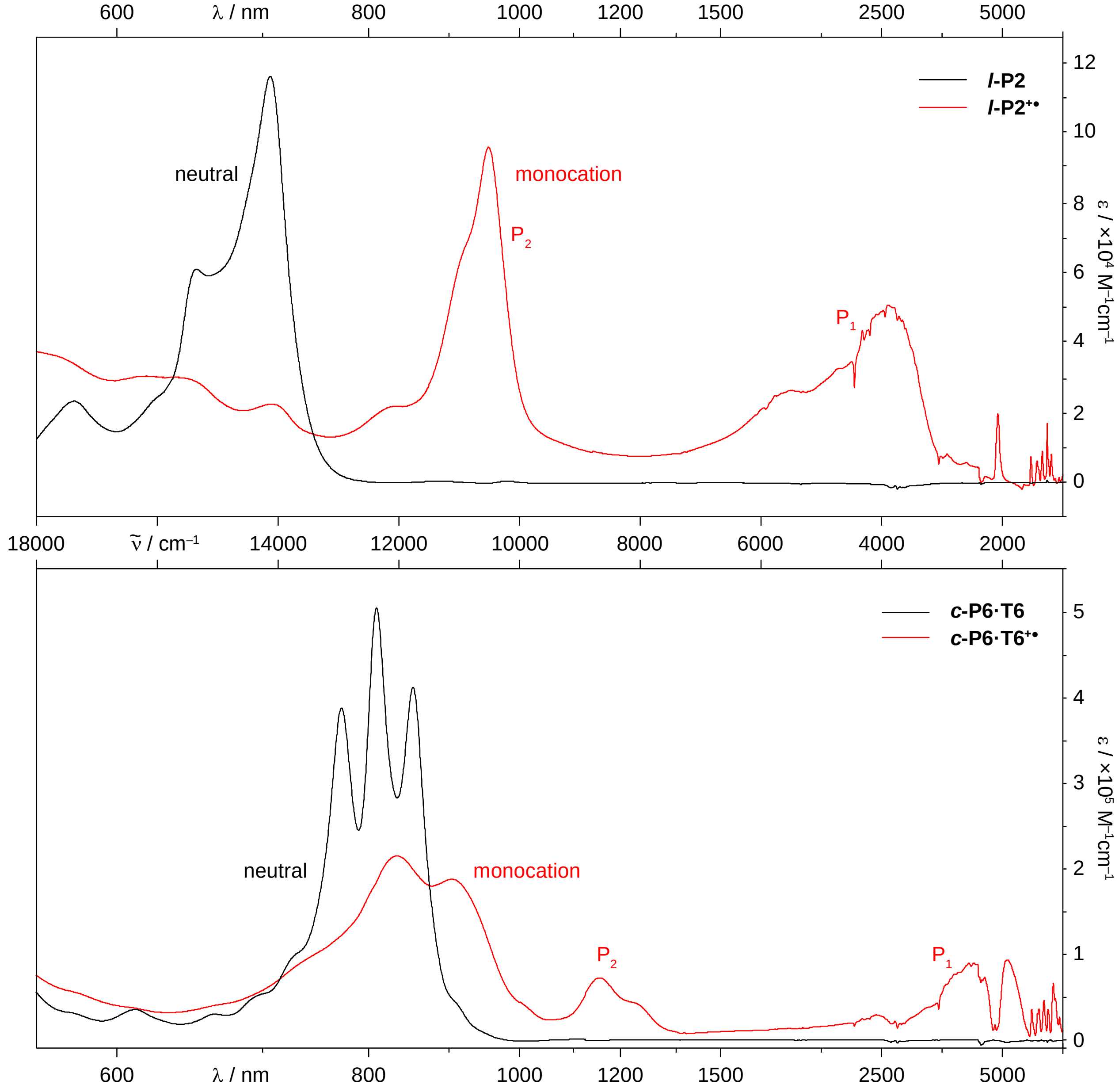

Fig. S2. UV-vis-NIR absorption spectra of a) ***l*-P2**/***l*-P2**$^{+\bullet}$ and b) ***c*-P6·T6**/***c*-P6·T6**$^{+\bullet}$ in CD_2Cl_2 with 0.1 M TBAP as an electrolyte. The distinctive porphyrin Q bands (neutral, λ = 700 nm and 800 nm for ***l*-P2**$^{+\bullet}$ and ***c*-P6·T6**$^{+\bullet}$ respectively) and P_2 bands (monocation) were used to estimate composition during sample preparation.

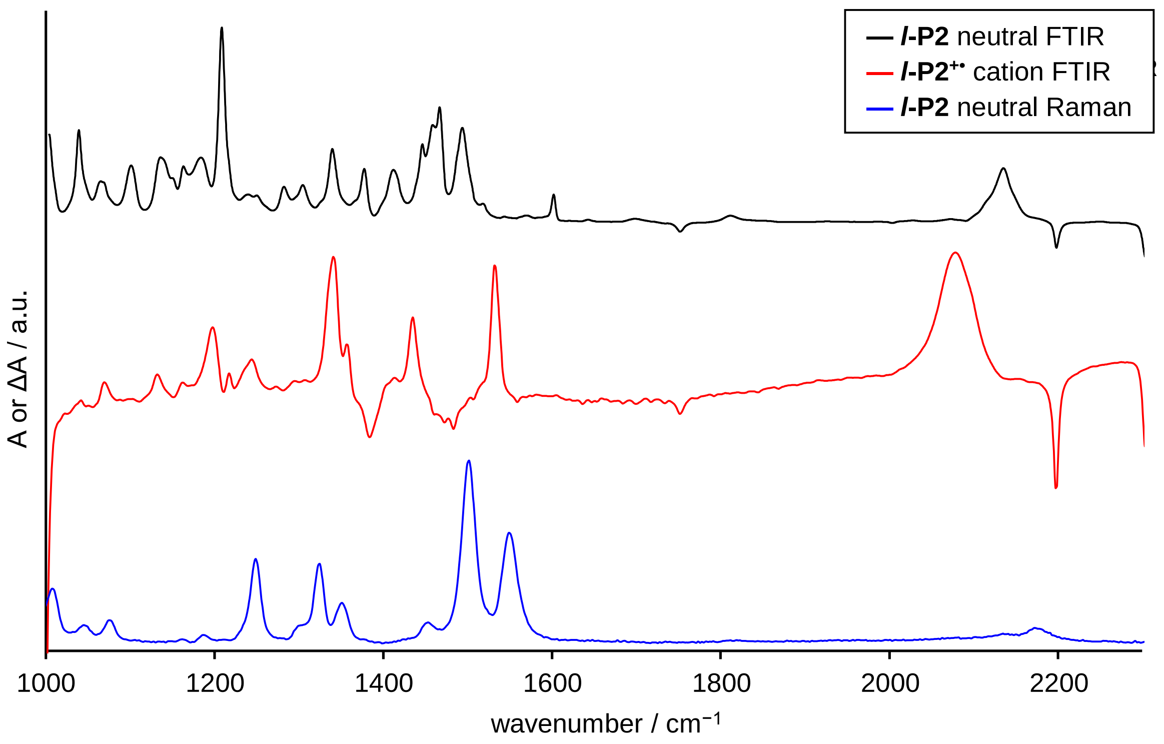

Fig. S3. FTIR of neutral ***l*-P2** (black), cation ***l*-P2**+• (red) and Raman spectra of neutral ***l*-P2** in CD_2Cl_2. Negative signals are solvent subtraction artefacts from CD_2Cl_2.

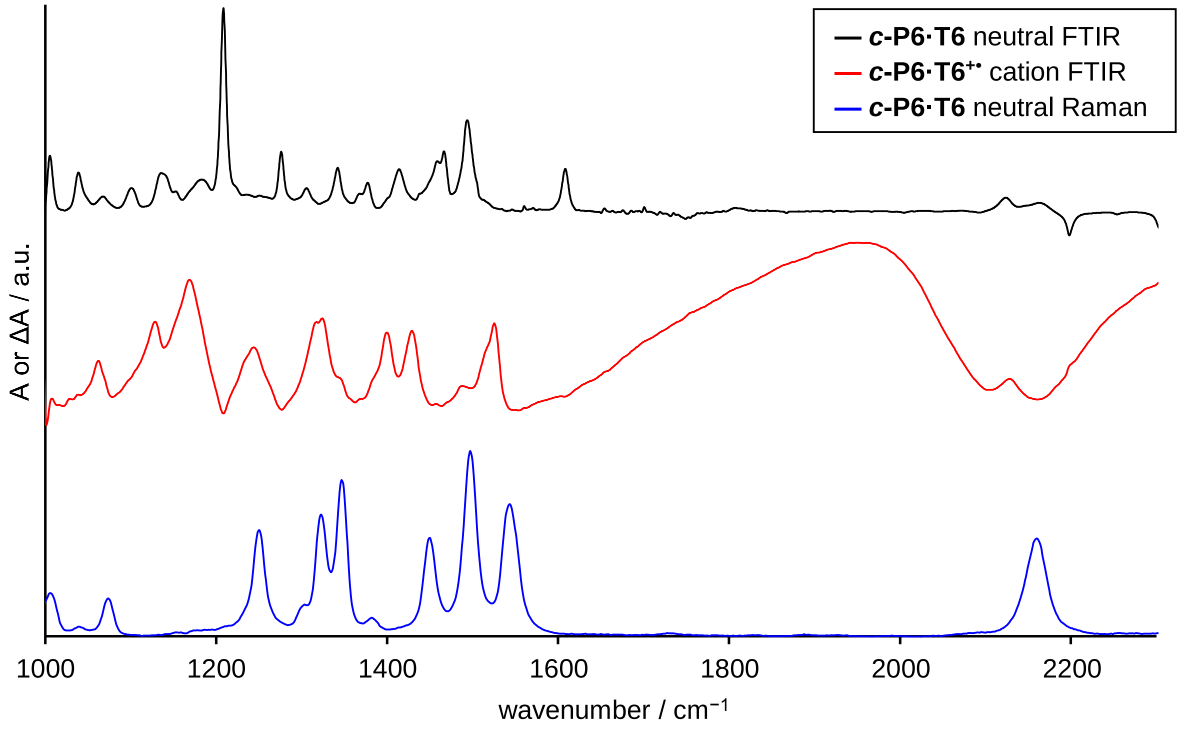

Fig. S4. FTIR of neutral ***c*-P6·T6** (black), cation ***c*-P6·T6**+• (red) and Raman spectra of neutral ***c*-P6·T6** CD_2Cl_2. Negative signals are solvent subtraction artefacts from CD_2Cl_2.

UV-vis-NIR absorption spectra of the OTTLE cell mixtures were recorded at three stages for each TRIR kinetic experiment. First, the neutral species was measured to ensure cleanliness and purity of the prepared sample. The sample was then oxidized, and the UV-vis-NIR recorded to ensure a suitable mixture of neutral and monocationic oligomers. The TRIR measurement was then performed and the UV-vis-NIR recorded again to ensure the oxidation state had not drifted significantly. **Fig. S5** shows representative example of the three consecutive spectra which were considered to have a suitable mixture of neutral/monocationic states, and which were acceptably stable (in term of potential and composition drift).

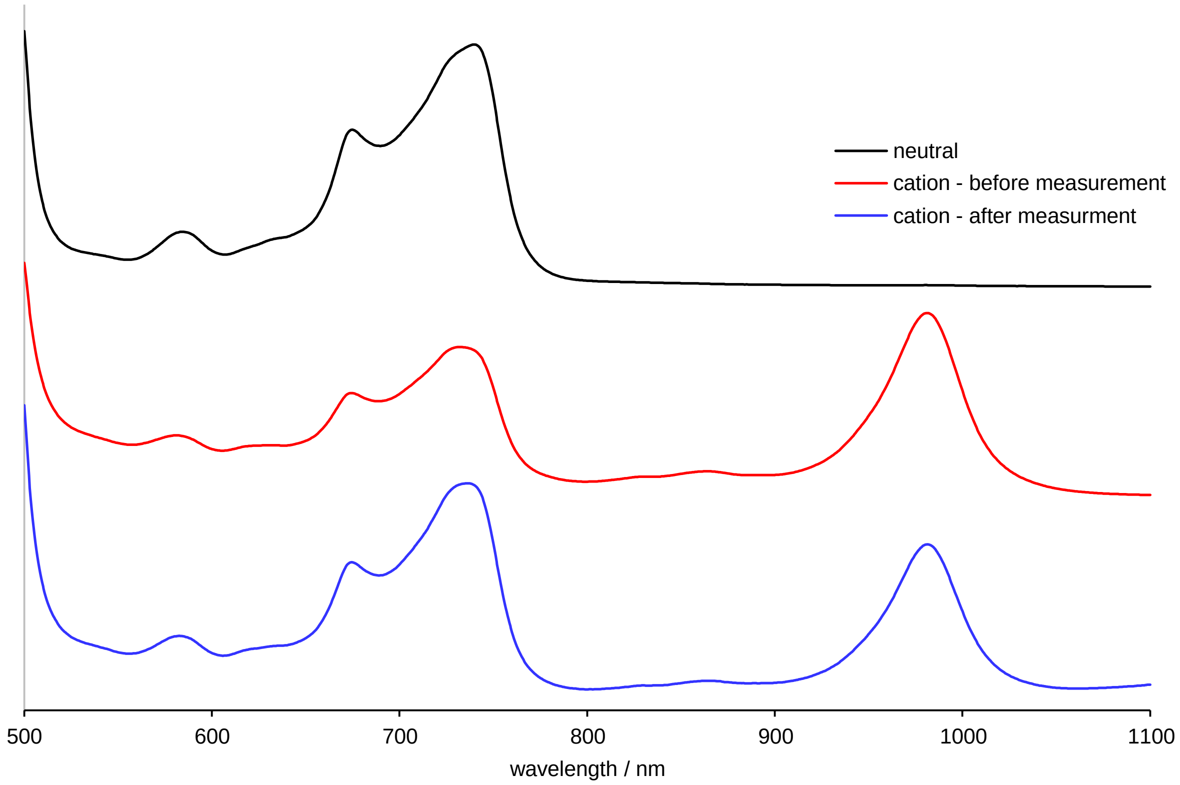

Fig. S5. Representative series of UV-vis-NIR absorption spectra (from TRIR experiment pumping 2050 cm^{-1} and probing fingerprint region, **Fig. S39**): ***l*-P2** before oxidation (black), after oxidation reaching ~1:1 mixture of ***l*-P2**/***l*-P2**$^{+\cdot}$ (red) and after ~30 min experiment (blue).

2 2D-IR

2.1 Measurement of Neutral (*l*-P2 and *c*-P6·T6) using ULTRA

The 2D-IR spectra of neutral compounds ***l*-P2** and ***c*-P6·T6** in the fingerprint region (1200–1600 cm^{-1}, **Fig. S6 a** and **b** respectively) were recorded in order to confirm, by the absence of similar peaks in the monocation spectra **Fig. 3** (main text), that all signals in the latter arose from monocation. The 2D-IR of the neutral were undertaken on samples > 10× more concentrated than for the spectroelectrochemical measurements.

The neutral spectra are simple in nature, showing a strong on-diagonal peak at ~1500 cm^{-1}. The on-diagonal feature manifests as a pair of signals, comprising a negative ground state bleach signal (GSB) with an associated positive excited-state absorption (ESA) corresponding to $v_2 \leftarrow v_1$ transitions at lower energy. The ESAs are red-shifted from the GSB due to vibrational anharmonicity.

The neutral compounds show some evidence of off-diagonal coupling, although these signals are an order of magnitude smaller than the main on-diagonal peak at 1500 cm^{-1}. Interestingly, very few of the bands identified in the FTIR show on-diagonal signals, perhaps as a result of small anharmonicities and consequent cancellation of positive ESA and negative GSB signals, or due to the quadratic dependence of transient signals on extinction coefficients (i.e. any 2D-IR signals were below the measurement capability of our instrument).[8] In any case, we do not expect significant contamination of monocation spectra (shown in **Fig. 3** main text) with neutral, with the exception of the strong on-diagonal signal at ~1500 cm^{-1}, due to the > 10× smaller concentration of ***l*-P2** and ***c*-P6·T6** in the SEC measurements.

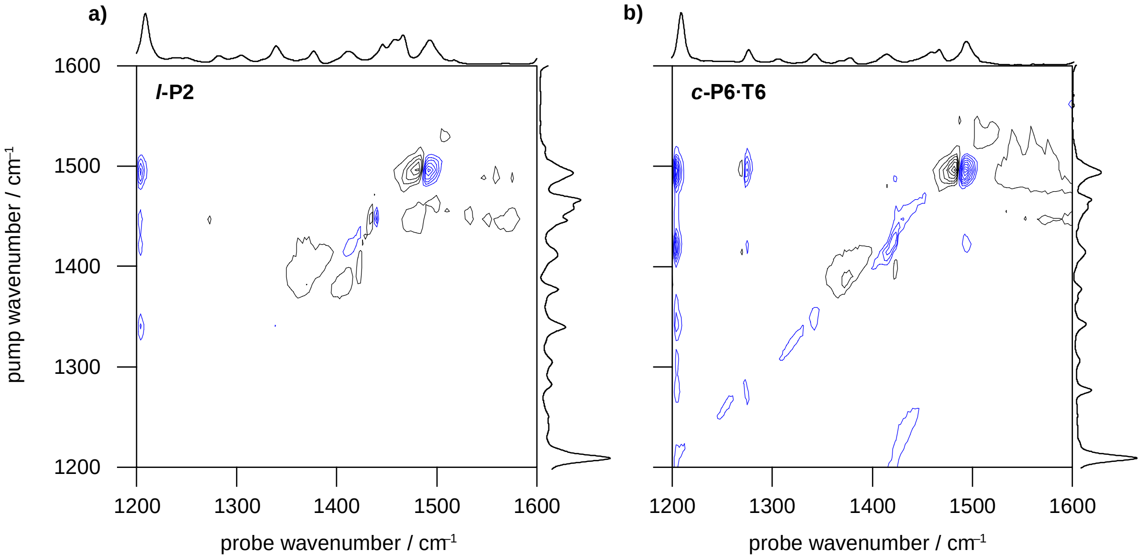

Fig. S6. 2D-IR spectra of neutral ***l*-P2** (a) and ***c*-P6·T6** (b) at 400 fs delay. Black solid and blue dashed contour lines correspond to positive (ESA) and negative (GSB) signals, respectively. Contours near zero are hidden for clarity. Spectra were recorded using a broadband pump centered at ~1325 cm^{-1} (FWHM ~150 cm^{-1}). Optical densities of all samples were ~0.2–0.4 in the fingerprint region.

2.2 Measurement of *l*-P2$^{+•}$ and *c*-P6·T6$^{+•}$ at later times (1.5 ps) using ULTRA

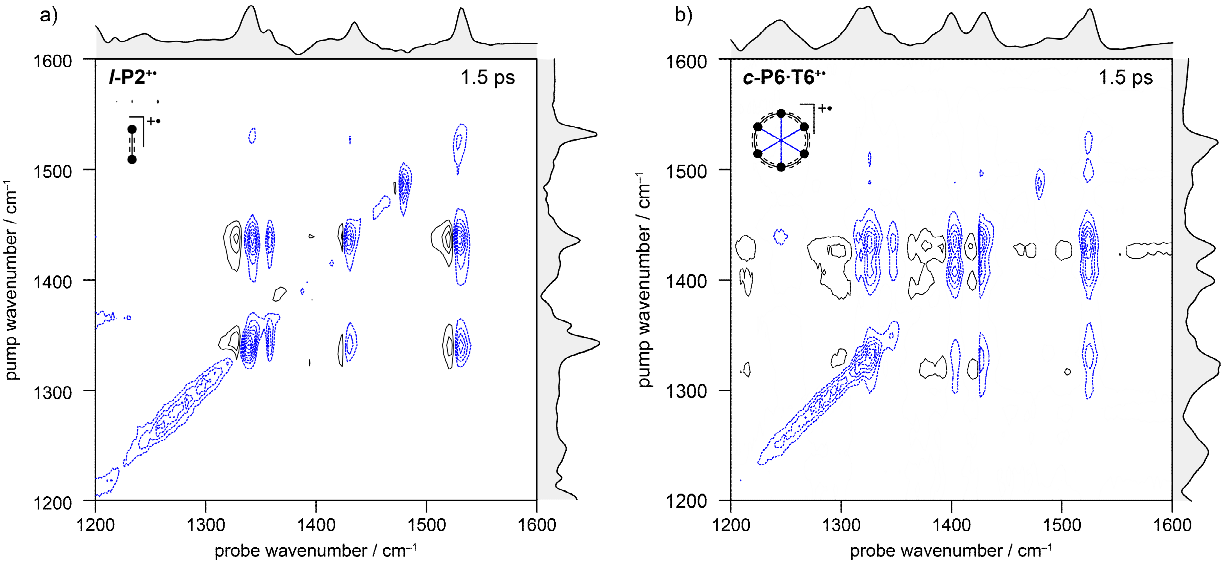

Fig. S7. 2D-IR spectra of monocation ***l*-P2$^{+•}$** (a) and ***c*-P6·T6$^{+•}$** (b) at 1500 fs delay. Black solid and blue dashed contour lines correspond to positive (ESA) and negative (GSB) signals, respectively. Contours near zero are hidden for clarity. Spectra were recorded using a broadband pump centered at ~1325 cm^{-1} (FWHM ~150 cm^{-1}). Optical densities of all samples were ~0.2–0.4 in the fingerprint region.

2.3 Fitting of *l*-P2$^{+•}$ LIFEtime 2D-IR on-diagonal slices (ω_{pump} = 2070 cm^{-1})

A slice of the LIFEtime 2D-IR at ω_{pump} = 2070 cm^{-1} for each time delay from 0.2 ps to 4.6 ps was extracted and fit to the sum of two Voigt profiles using the built-in non-linear curve fitting in OriginPro (user-defined function consisting of two Voigt profiles). The GSB Voigt was constrained to a center position of 2084 cm^{-1} and a FWHM of 41.3 cm^{-1}. Both values were extracted from unconstrained fits at later times. The second voigt function (representative of the ESA) was allowed to vary. A representative slice, the fitted function, and the peak-peak separation, Δv, are plotted in main text **Fig. 4**. The FWHM of the ESA (the free Voigt) is shown in **Fig. S8**.

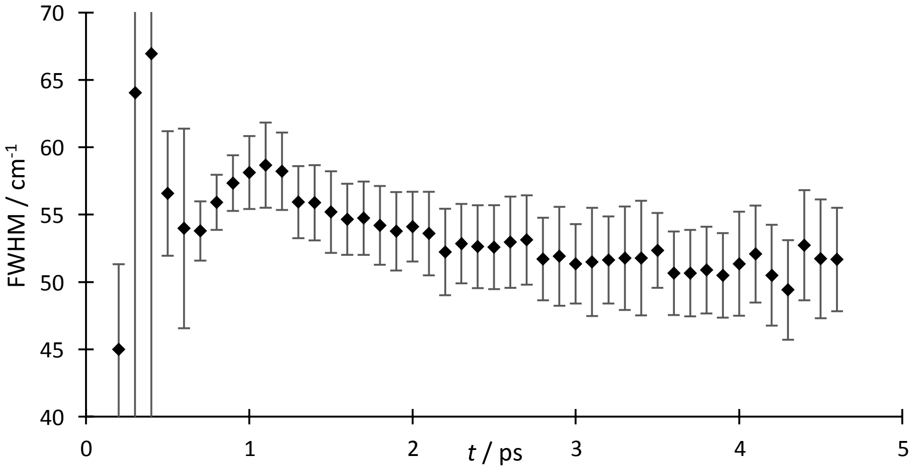

Fig. S8. Fitted FWHM for the ESA Voigt profile from the fitted on-diagonal pair for ***l*-P2$^{+•}$**. Error bars given as ± SE.

2.4 Fitting of LIFEtime 2D-IR kinetic traces

The evolution of the radical cation systems over time are complicated. Intense GSB features, crowded spectra, ESA grow ins and broad ESA 'baseline'-like features all complicate analysis of the underlying kinetics. As such, we thought it instructive to fit the features to the simplest kinetic model possible (that is, comprising of the fewest decay constants) in order to present the salient features of the spectra and their evolution with time.

Much of the signal evolution happens at very early times for both species (t < 300 fs). As such, we attempted to deconvolute the instrument response from early transient spectral events using Equation (S1), derived by Mouton et al,[9] which describes the convolution of the instrument response with the time-dependent concentration profiles of spectrokinetic data.

$$C_i^*(t,\sigma) = \sum_{j=1}^{N} A_j \exp\left(\frac{k_j^2\sigma^2}{2} - k_j t\right)\left(1 - erf\left(\frac{k_j\sigma^2 - t}{\sqrt{2}\sigma}\right)\right) \qquad \text{(S1)}$$

where C_i^* is the convoluted kinetic profile, t is the delay time after photoexcitation, σ is the full width half maximum (FWHM) of the Gaussian instrument response function, A_j are the pre-exponential factors and k_j are the decay rates (which can be expressed as lifetimes: $\tau_j = 1/k_j$). σ was fit globally across all the kinetic profiles for GSB recovery.

Time-resolved 2D-IR spectra were visualized in realtime using a home-made Python program that allowed for extraction of decay profiles at given points in the 2D spectra. Decay profiles were chosen at the center points of the peaks at early times (0.4 ps) and at slightly later times (0.7 ps) for picking out ESAs hidden at early time. Visual inspection showed most decay profiles were largely independent of small adjustments in peak positions and as such rigorous 2D peak fitting was not required. Some peaks were omitted due to non-trivial decay profiles (e.g. 'dips' or noise due to overlapping features at early times). The start time of all GSB profiles were truncated to a starting time of − 0.2 ps to avoid negative-time interference effects but retain a 'rise' to allow for fitting of the IRF and consequent extraction of early time data. The start time of ESA absorptions were truncated to ~ 0.6 ps and 1 ps for ***l*-P2$^{+\bullet}$** and ***c*-P6·T6$^{+\bullet}$** respectively to avoid signal convolution with grow-in, and in the case of ***c*-P6·T6$^{+\bullet}$** allow the ESAs to become resolved from the dominating bleaches at early times.

The selected peaks were categorized into 'types', which seem to show slightly different character. These were: 'On-diagonal GSB', 'Off-diagonal GSB', 'ESA peaks' (i.e. sharper ESA peaks, typical for 2D-IR) and 'Broad ESA absorption' (i.e. a broad raised baseline observed across the spectrum).

Fits were undertaken using the symfit[10] Python package to allow global fitting of shared variables using eq. S1.

For ***l*-P2$^{+\bullet}$** mono-exponential fits (N = 1) were poor for all peak types except the broad ESA transient, so the model was made explicitly bi-exponential for the other 3 groups (N = 2 in eq. S1). Fitting these to the model with unconstrained and unshared time constants revealed two useful pieces of information: firstly, that the on-diagonal signals fit poorly to a bi-exponential profile, and secondly that the rest of the decay profiles seemed to share time constants of a similar value. Consequently, a model was built to fit the ***l*-P2$^{+\bullet}$** data to the minimum number of decay constants, and can be summarized as follows:

Peak type	Model	Time constant (globally shared)
On-diagonal GSB	Tri-exponential	k_1, k_2, k_3
Off-diagonal GSB	Bi-exponential	k_2, k_3
ESA peaks	Bi-exponential	k_2, k_3
ESA broad transient	Mono-exponential	k_2

Table S1. Model for the fitting of ***l*-P2$^{+•}$** kinetic data.

For ***c*-P6·T6$^{+•}$** mono-exponential fits ($N = 1$) were poor for all peak types including the broad ESA transient, so the model was made explicitly bi-exponential for all 4 signal types ($N = 2$ in eq. S1). Fitting these to the model with unconstrained and unshared time constants showed the existence of a single shared time constant across all peaks and two others, one shared between the GSBs and one shared for the ESAs. Consequently, a model was built to fit the ***c*-P6·T6$^{+•}$** data to the minimum amount of decay constants, and can be summarized as follows:

Peak type	Model	Time constant (globally shared)
On-diagonal GSB	Bi-exponential	k_1, k_3
Off-diagonal GSB	Bi-exponential	k_1, k_3
ESA peaks	Bi-exponential	k_2, k_3
ESA broad transient	Bi-exponential	k_2, k_3

Table S2. Model for the fitting of ***c*-P6·T6$^{+•}$** kinetic data.

The instrument response was shared across all data sets and was fitted as $\sigma = 183 \pm 7$ and $\sigma = 191 \pm 7$ fs for ***l*-P2$^{+•}$** and ***c*-P6·T6$^{+•}$**, respectively. This is slightly faster than the reported instrument response in the same spectrometer (250-300 fs) but the similarity between data sets suggests a reasonable estimation for our purposes.

Errors are given as one standard deviation, calculated using the built-in symfit function.

2.4.1 *l*-P2$^{+•}$

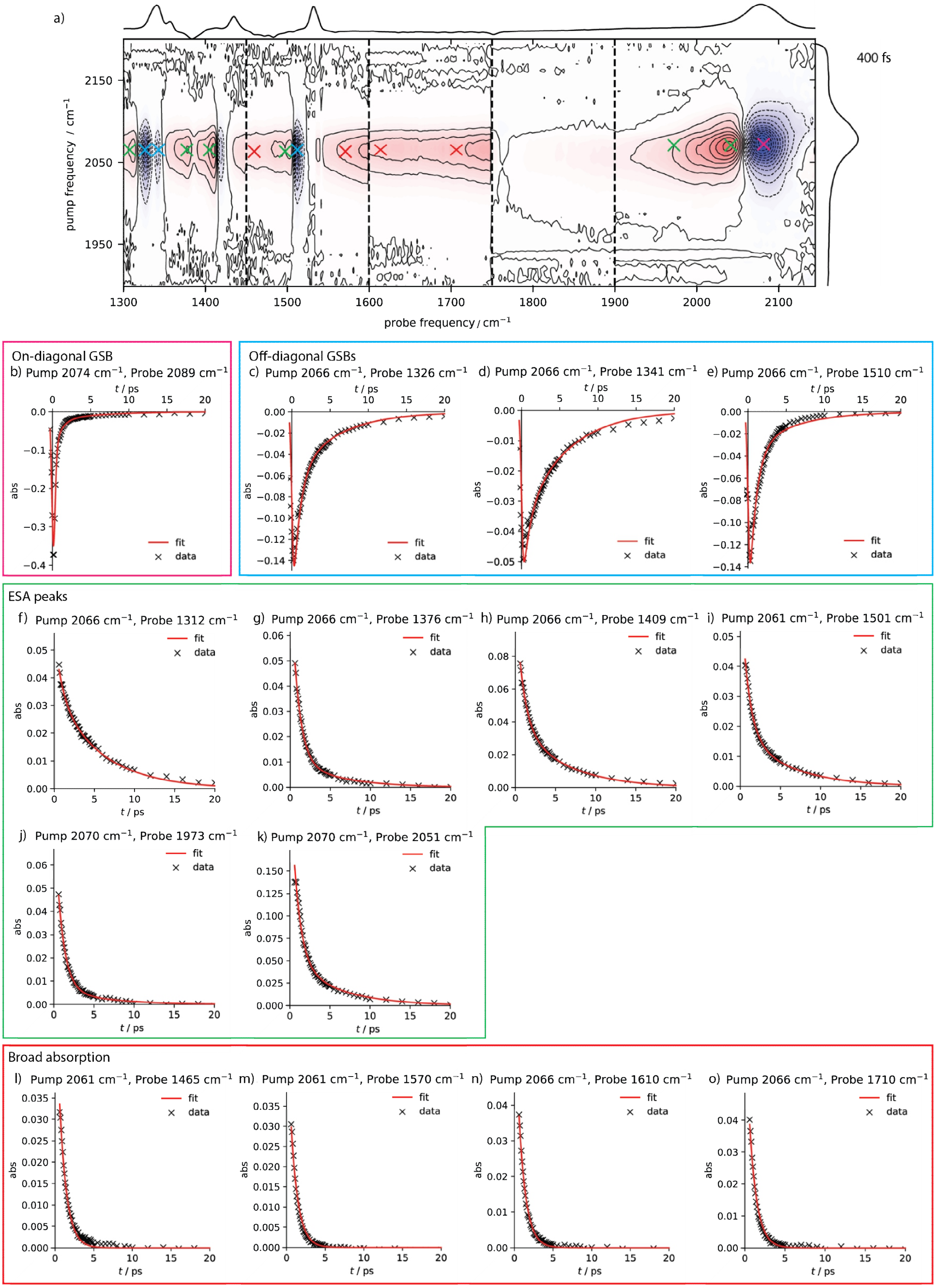

Fig. S9. a) 2D-IR spectra of ***l*-P2$^{+•}$** at 0.4 ps showing the selected peaks for peak fitting with colored crosses. Pump centered at 2050 cm^{-1} (FWHM ~80 cm^{-1}). Individually recorded windows are stitched

together for clarity by normalizing to the 2050 cm^{-1} region (see main text). Red/blue correspond to positive(ESA)/negative(GSB) signals. b-o) Fitted plots for the selected peaks. GSB at ~ 1520 cm^{-1} omitted due to non-trivial decay shape caused by overlap with broad ESA transient.

Peak type	ω / cm^{-1} pump	ω / cm^{-1} probe	τ_1 / ps	% intensity	τ_2 / ps	% intensity	τ_3 / ps	% intensity
On-diagonal GSB	2074	2089	0.152 ± 0.015	89.8 ± 9.8	0.917 ± 0.010	8.08 ± 2.01	5.70 ± 0.13	2.13 ± 0.43
Off-diagonal GSB	2066	1326	-	-	0.917 ± 0.010	68.2 ± 9.2	5.70 ± 0.13	31.8 ± 4.0
	2066	1341	-	-	0.917 ± 0.010	39.5 ± 7.4	5.70 ± 0.13	60.5 ± 6.1
	2066	1510	-	-	0.917 ± 0.010	80.6 ± 8.6	5.70 ± 0.13	19.4 ± 2.8
ESA peaks	2066	1312	-	-	0.917 ± 0.010	36.9 ± 3.2	5.70 ± 0.13	63.1 ± 2.7
	2066	1376	-	-	0.917 ± 0.010	86.9 ± 1.8	5.70 ± 0.13	13.1 ± 0.5
	2066	1409	-	-	0.917 ± 0.010	64.0 ± 15.2	5.70 ± 0.13	36.0 ± 4.8
	2066	1501	-	-	0.917 ± 0.010	71.4 ± 2.1	5.70 ± 0.13	28.6 ± 0.9
	2070	1973	-	-	0.917 ± 0.010	91.4 ± 1.9	5.70 ± 0.13	8.63 ± 0.41
	2070	2051	-	-	0.917 ± 0.010	79.8 ± 3.4	5.70 ± 0.13	20.2 ± 1.0
ESA broad transient	2061	1465	-	-	0.917 ± 0.010	100	-	-
	2061	1570	-	-	0.917 ± 0.010	100	-	-
	2066	1610	-	-	0.917 ± 0.010	100	-	-
	2066	1710	-	-	0.917 ± 0.010	100	-	-

Table S3. Fitted parameters for ***l*-P2**$^{+\bullet}$ from the decay profiles presented in **Fig. S9**. % are presented in lieu of normalized intensities and were calculated by the ratio of pre-exponential factors. Errors given as ± 1 S.D.

2.4.2 *c*-P6·T6$^{+•}$

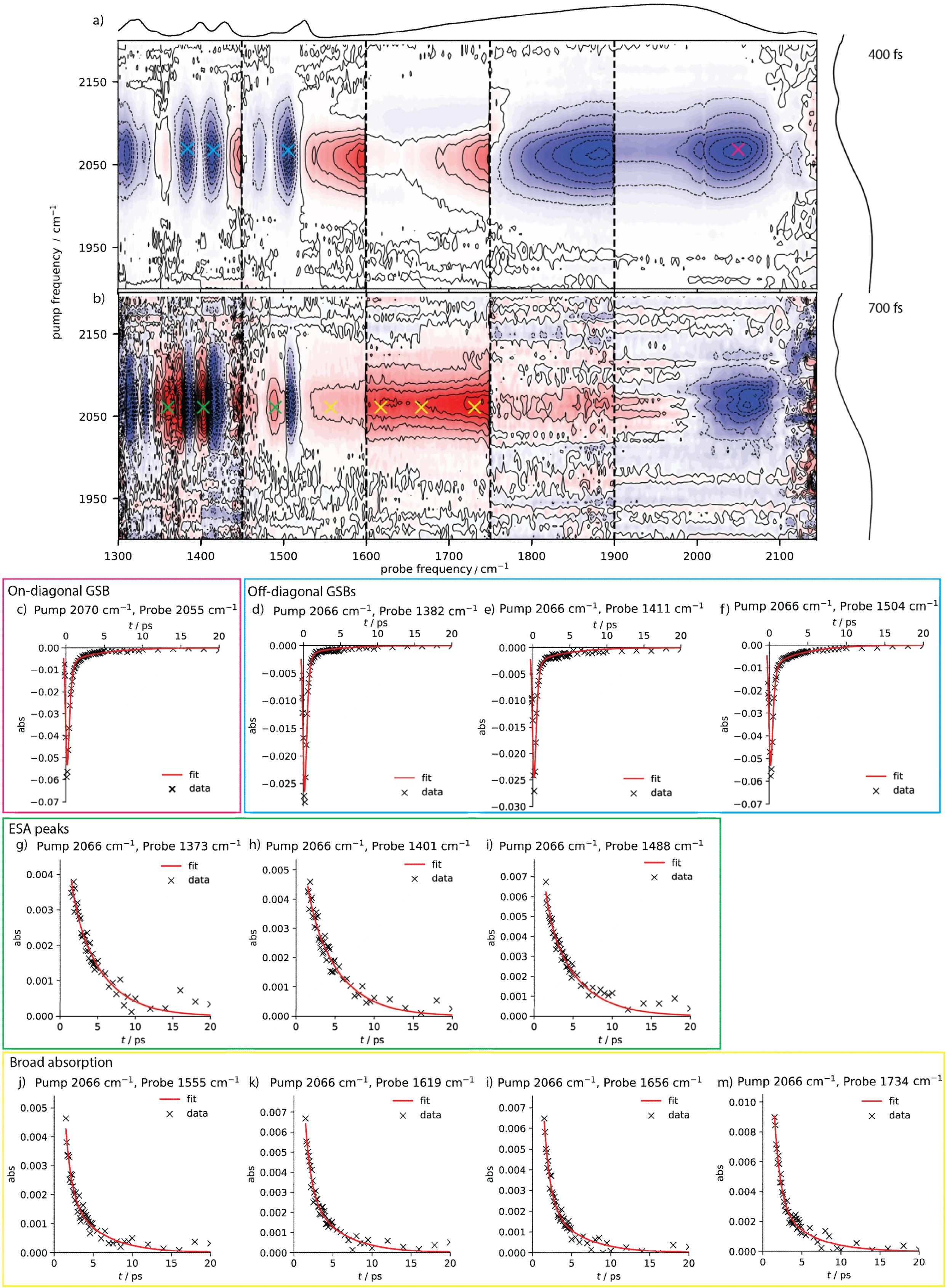

Fig. S10. 2D-IR spectra of ***c*-P6·T6$^{+•}$** at a) 0.4 ps and b) 0.7 ps. Colormaps are *not* shared between the two time delays in order to highlight ESA features that are normally lost due to the intense bleach.

Pump centered at 2050 (FWHM ~80 cm^{-1}). Individually recorded windows are stitched together for clarity by normalizing to the 2050 cm^{-1} region (see main text). Red/blue correspond to positive(ESA)/negative(GSB) signals. c-m) show fitted decay traces for the peaks denoted in a) and b) by colored crosses. The GSB at 1320 cm^{-1} and 1430 cm^{-1} are omitted due to non-trivial decay profiles caused by their vicinity to the edge of the spectral window.

Peak type	ω / cm^{-1} pump	ω / cm^{-1} probe	τ_1 / ps	% intensity	τ_2 / ps	% intensity	τ_3 / ps	% intensity
On-diagonal GSB	2070	2055	0.287 ± 0.012	94.2 ± 6.5	0.287 ± 0.012	94.2 ± 6.5	4.11 ± 0.26	5.83 ± 0.80
Off-diagonal GSB	2066	1382	0.287 ± 0.012	97.4 ± 5.9	0.287 ± 0.012	97.4 ± 5.9	4.11 ± 0.26	2.60 ± 0.59
	2066	1411	0.287 ± 0.012	94.6 ± 7.4	0.287 ± 0.012	94.6 ± 7.4	4.11 ± 0.26	5.37 ± 0.94
	2066	1504	0.287 ± 0.012	91.7 ±7.3	0.287 ± 0.012	91.7 ±7.3	4.11 ± 0.26	8.26 ± 1.03
ESA peaks	2066	1373	-	-	0.744 ± 0.072	43.6 ± 27.4	4.11 ± 0.26	56.4 ± 16.7
	2066	1401	-	-	0.744 ± 0.072	38.7 ± 28.5	4.11 ± 0.26	61.3 ± 19.3
	2066	1488	-	-	0.744 ± 0.072	54.3 ± 21.0	4.11 ± 0.26	45.7 ± 10.2
ESA broad transient	2066	1556	-	-	0.744 ± 0.072	85.3 ± 21.8	4.11 ± 0.26	14.7 ± 3.0
	2066	1619	-	-	0.744 ± 0.072	85.7 ± 21.4	4.11 ± 0.26	14.3 ± 2.8
	2066	1656	-	-	0.744 ± 0.072	88.9 ± 22.2	4.11 ± 0.26	11.1 ± 2.3
	2066	1735	-	-	0.744 ± 0.072	90.1 ± 22.7	4.11 ± 0.26	9.93 ± 2.10

Table S4: Fitted parameters for ***c*-P6·T6$^{+\bullet}$** from the decay profiles presented in **Fig. S10**. % are presented in lieu of normalized intensities and were calculated by the ratio of pre-exponential factors. Errors given as ± 1 S.D.

2.4.3 Grow-in fitting

To prove that the grow-in at early times is consistent to with an energy transfer/IVR process with the same time constants, we attempted to fit the ESA grow-ins to a kinetic model. The mono-exponential dataset fit well to the equation

$$abs = \frac{k_1}{k_2 - k_1}(e^{-k_1 t} - e^{-k_2 t})a_0 \qquad \text{(S2)}$$

where a_0 is the arbitrary absorbanceand is the only variable in the fit. k_n are the inverse of the values presented in Tables S3,4. Fits are shown in **Fig. S11**.

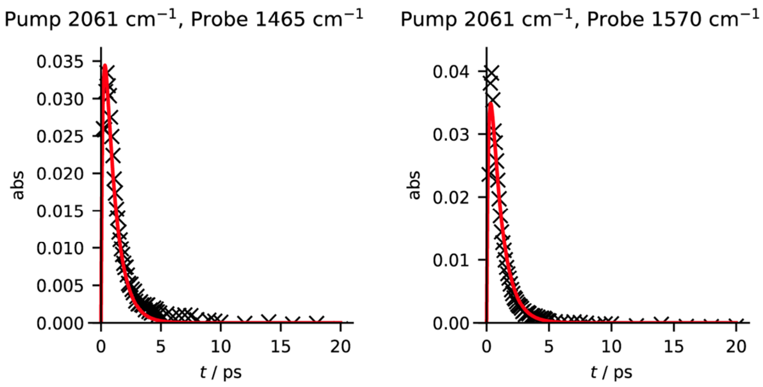

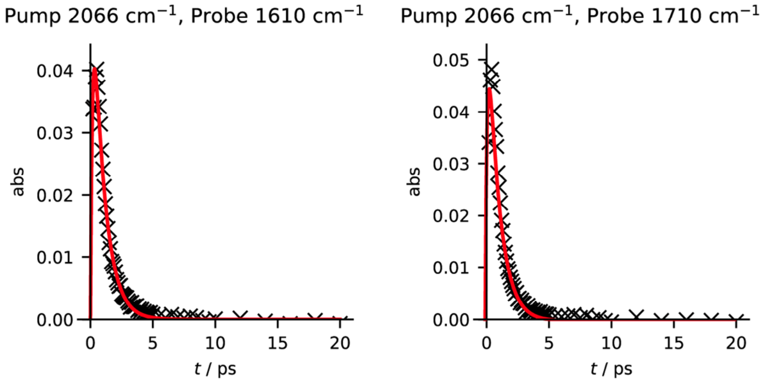

Fig. S11: Fits of the mono-exponential ***l*-P2$^{+•}$** ESAs to eqn S2.

The bi-exponential cases are more complicated. It is clear that not all of the initial IVR population is transferred to the remaining two decaying states, and so an equation of the form of eqn S2 with k_2 substituted with $k_2 + k_3$ is a poor model due to the shared a_0 (and does not fit the data). Another potential model would be a special case where the three decay processes transfer energy to eachother linearly, in an $A \overset{k_1}{\rightarrow} B \overset{k_2}{\rightarrow} C \overset{k_3}{\rightarrow} D$ model, where B and C are the indistinguishable probed processes and D is the ground state. The observed absorbance is given by [B] + [C], which assuming [B] = [C] = 0 at t = 0 gives the following equation:

$$abs = \left(\frac{k_1}{k_2 - k_1}\left(e^{-k_1 t} - e^{-k_2 t}\right) + \frac{k_1 * k_2}{k_1 - k_2} e^{-t(k_1 + k_2)} * \left(\frac{e^{k_1 t}}{k_2 - k_3} + \frac{e^{k_2 t}}{k_3 - k_1}\right)\right) a_0 \qquad \text{(S3)}$$

This equation also failed to fit the data. Instead, it seems most likely that the IVR process transfers differing amounts of population to the two subsequent states, and thus must satisfy the equation:

$$abs = \frac{k_1}{k_2 - k_1}\left(e^{-k_1 t} - e^{-k_2 t}\right) a_1 + \frac{k_1}{k_3 - k_1}\left(e^{-k_1 t} - e^{-k_3 t}\right) a_2 \qquad \text{(S4)}$$

The data fit well to this, as shown in **Figs. S12, S13**. Taking the percentages of a_1 and a_2 presented in **Tables S5, S6** and comparing them to the values in **Tables S3, S4** shows a strong correlation, agreeing with our previously presented model.

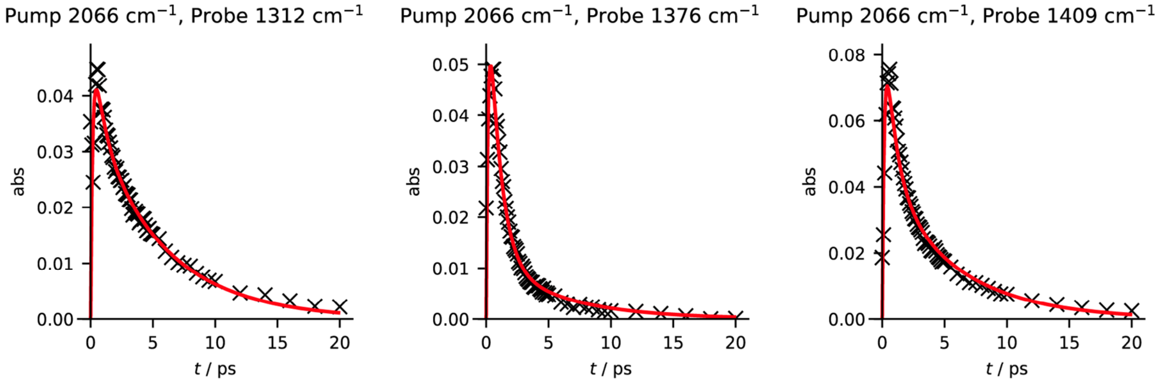

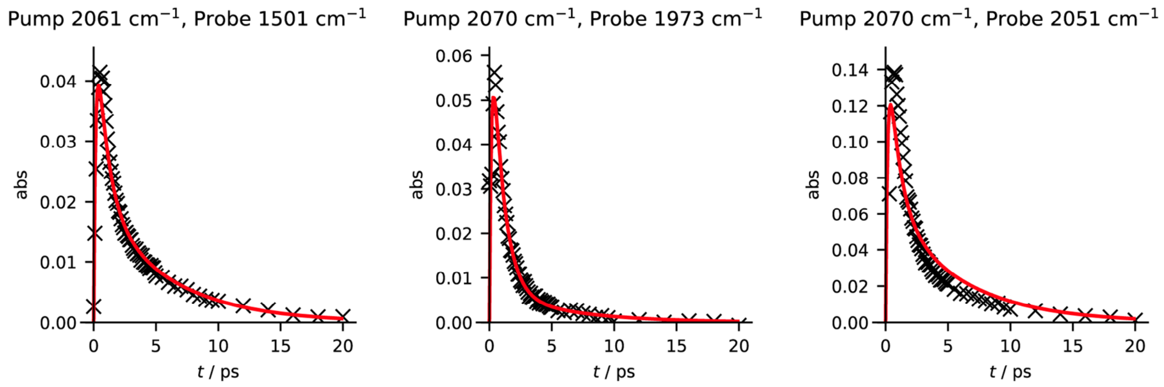

Fig. S12: Fits of the bi-exponential ***l*-P2$^{+\bullet}$** ESAs to eqn S4.

ω / cm^{-1}		% of k_3	% of k_2
**pump**	**probe**	**component**	**component**
2066	1312	71.3 ± 3.1	28.7 ± 7.6
2066	1376	17.3 ± 1.4	82.7 ± 3.44
2066	1409	47.8 ± 1.6	52.2 ± 3.8
2066	1501	39.6 ± 1.4	60.4 ± 3.3
2070	1973	10.5 ± 2.0	89.5 ± 4.8
2070	2051	32.0 ± 2.0	68.0 ± 5.2

Table S5: Percentage composition of the bi-exponential ***l*-P2$^{+\bullet}$** ESA decay traces fit in **Fig. S12**.

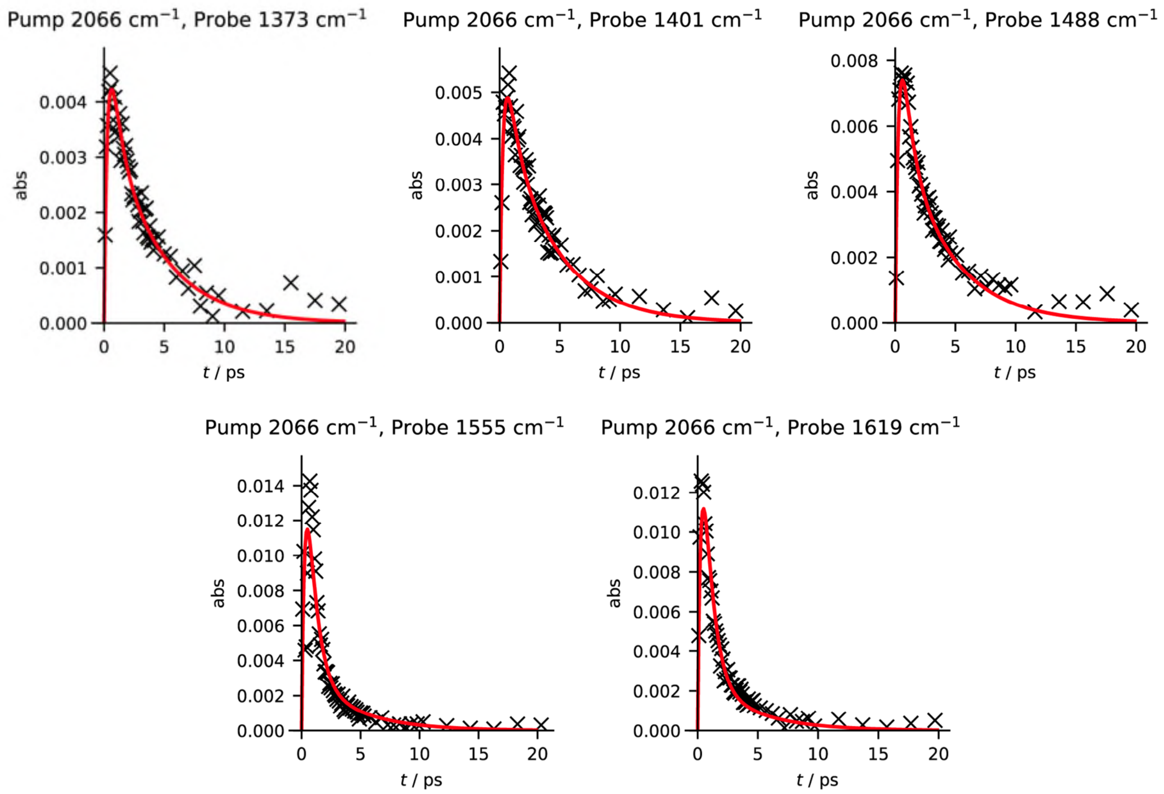

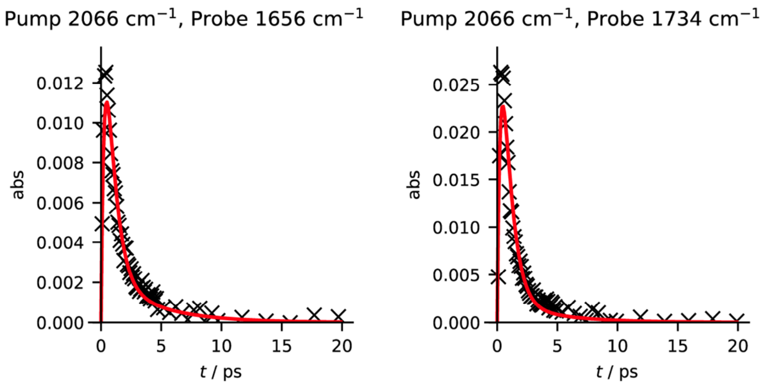

Fig. S13: Fits of the bi-exponential ***c*-P6·T6$^{+•}$** ESAs to eqn S4.

ω / cm^{-1}		% of k_3	% of k_2
**pump**	**probe**	**component**	**component**
2066	1373	61.7 ± 1.4	38.3 ± 3.6
2066	1401	68.9 ± 1.7	31.1 ± 4.2
2066	1488	54.1 ± 1.3	45.9 ± 3.3
2066	1555	17.3 ± 2.7	82.8 ±6.9
2066	1619	14.6 ± 1.2	85.4 ± 3.0
2066	1656	12.1 ± 1.1	87.9 ± 2.8
2066	1734	5.76 ± 1.36	94.2 ± 3.5

Table S6: Percentage composition of the bi-exponential ***c*-P6·T6$^{+•}$** ESA decay traces fit in **Fig. S13**.

2.5 Slices of time-resolved LIFEtime 2D-IR data

The unstitched data are presented below, corresponding to the four off-diagonal probe regions recorded by changing **probe a** (see main text) and the **probe b** (on-diagonal) data recorded with the 1250–1450 cm^{-1} region. A slice through the pump wavenumber of 2061 cm^{-1} is plotted vs time for each probe window to show the temporal evolution of the data. Magnified spectra showing the time region from −2–5 ps are included to show early time signal evolution.

2.5.1 *l*-P2$^{+\bullet}$

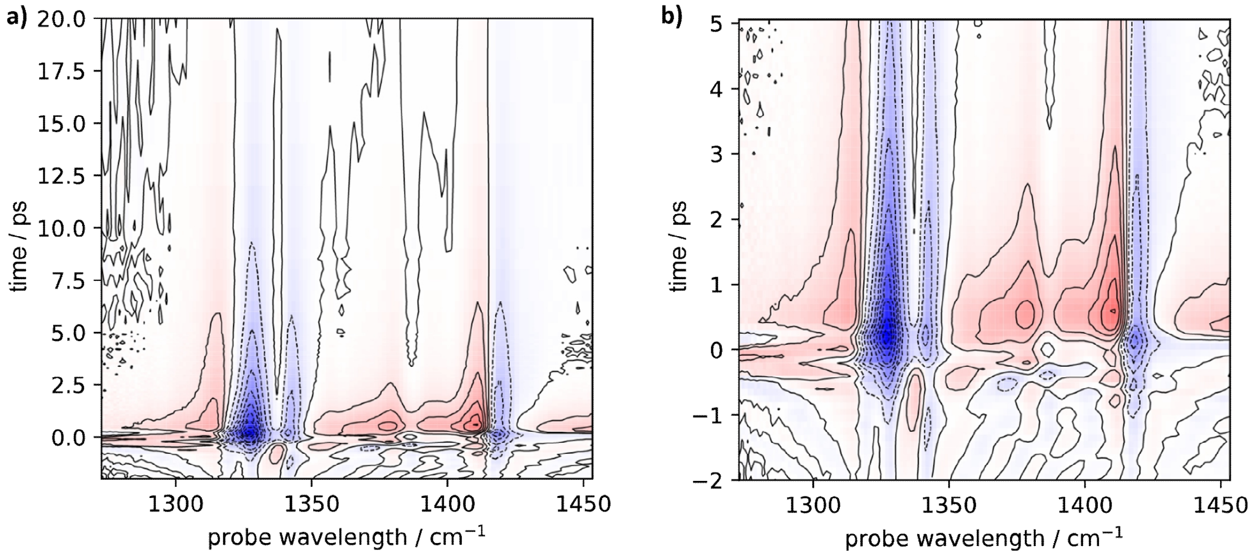

Fig. S14. A slice (ω_{pump} = 2061 cm^{-1}) of the 2D-IR LIFEtime data recorded for ***l*-P2$^{+\bullet}$** with **probe a** set to the region ~1250–1450 cm^{-1} showing (**a**) entire pump-probe delay series and (**b**) early time pump-probe delays. Red/blue correspond to positive(ESA)/negative(GSB) signals.

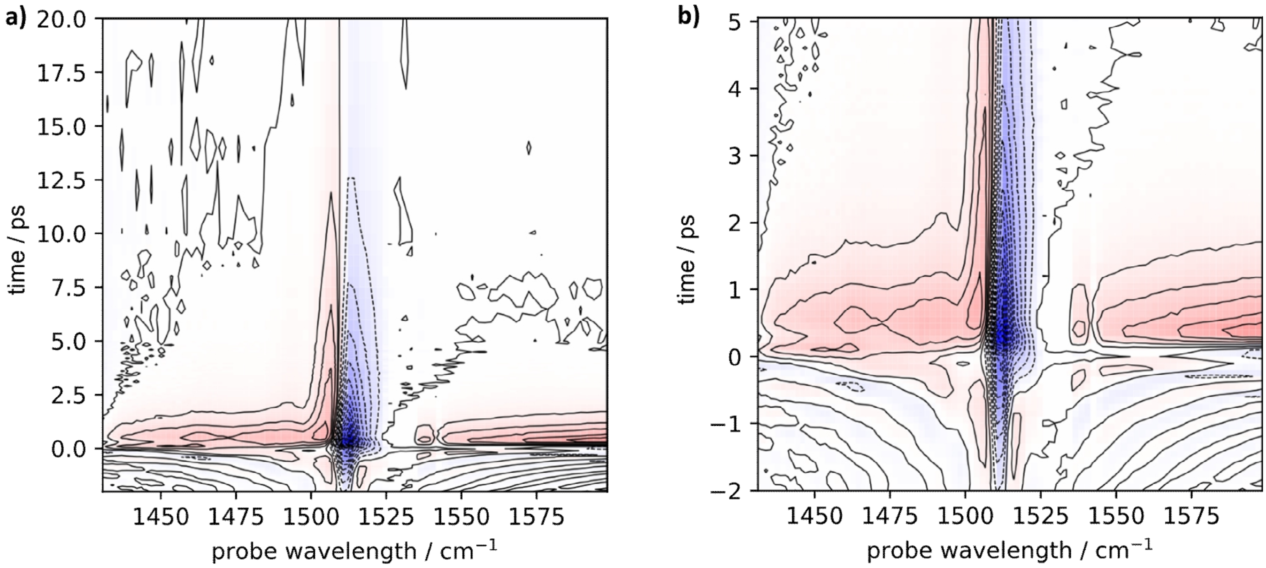

Fig. S15. A slice (ω_{pump} = 2061 cm^{-1}) of the 2D-IR LIFEtime data recorded for ***l*-P2$^{+\bullet}$** with **probe a** set to the region ~1400–1600 cm^{-1} showing (**a**) entire pump-probe delay series and (**b**) early time pump-probe delays. Red/blue correspond to positive(ESA)/negative(GSB) signals.

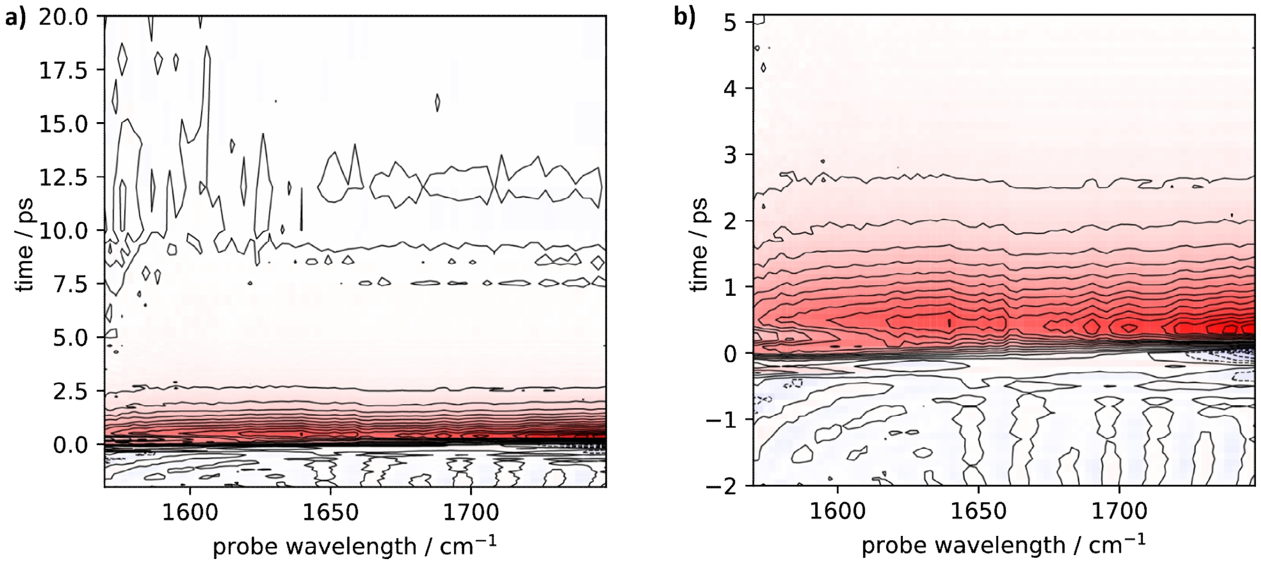

Fig. S16. A slice (ω_{pump} = 2061 cm^{-1}) of the 2D-IR LIFEtime data recorded for ***l*-P2$^{+•}$** with **probe a** set to the region ~1550–1750 cm^{-1} showing (**a**) entire pump-probe delay series and (**b**) early time pump-probe delays. Red/blue correspond to positive(ESA)/negative(GSB) signals.

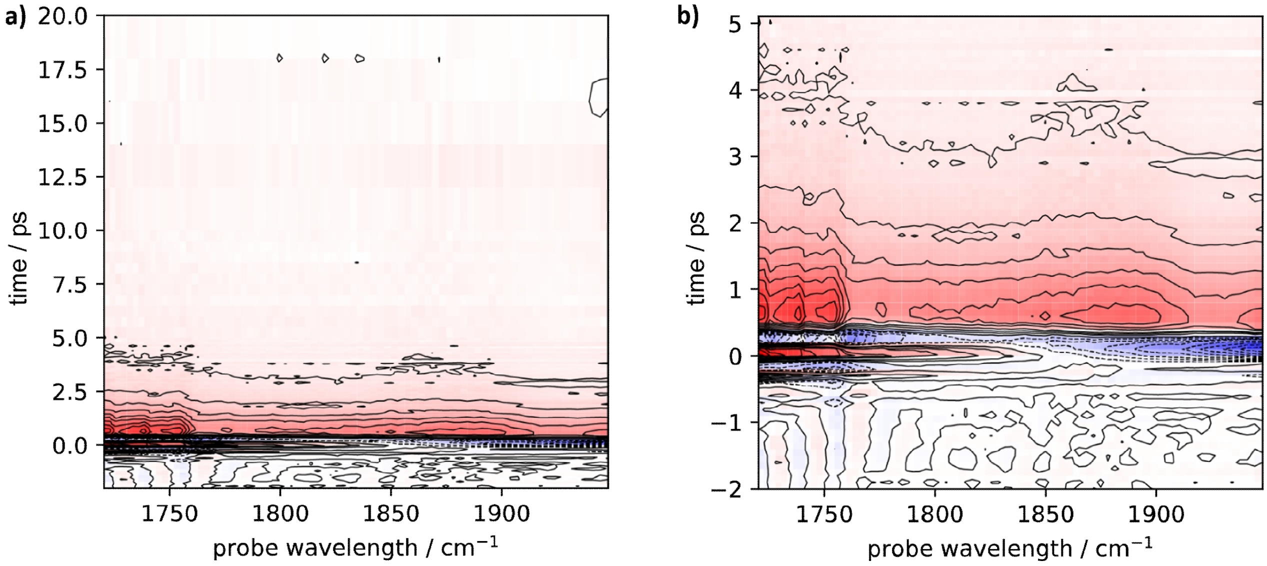

Fig. S17. A slice (ω_{pump} = 2061 cm^{-1}) of the 2D-IR LIFEtime data recorded for ***l*-P2$^{+•}$** with **probe a** set to the region ~1700–1950 cm^{-1} showing (**a**) entire pump-probe delay series and (**b**) early time pump-probe delays. Red/blue correspond to positive(ESA)/negative(GSB) signals.

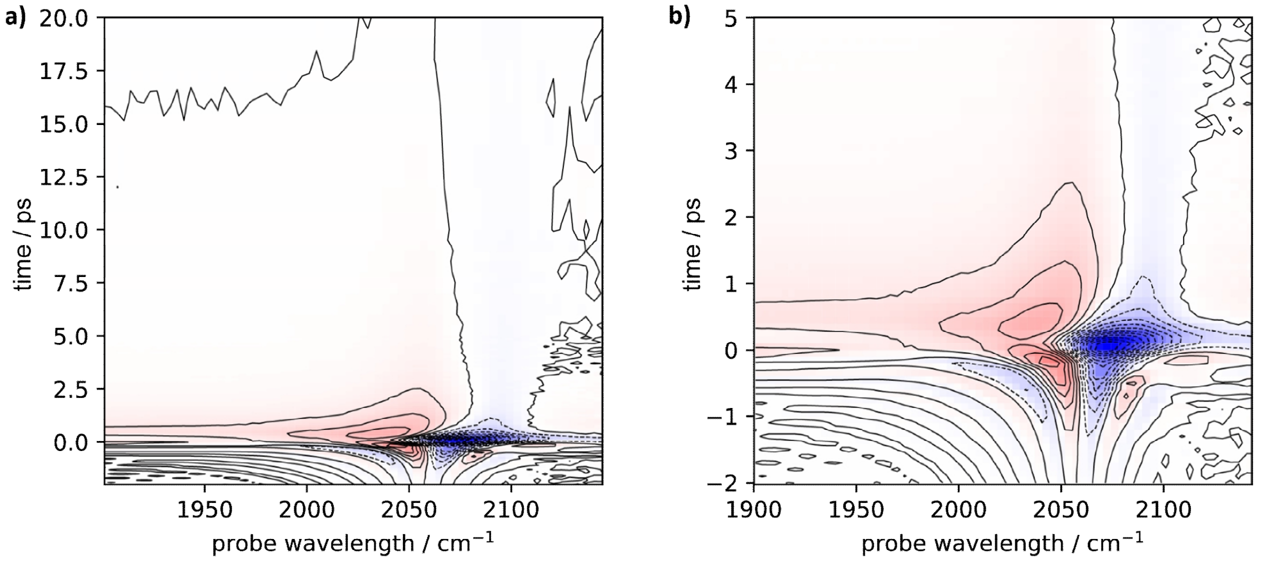

Fig. S18. A slice (ω_{pump} = 2061 cm^{-1}) of the 2D-IR LIFEtime data recorded for ***l*-P2$^{+•}$** with **probe b** set to the region ~1900–2150 cm^{-1} showing (**a**) entire pump-probe delay series and (**b**) early time pump-probe delays. Red/blue correspond to positive(ESA)/negative(GSB) signals.

2.5.2 *c*-P6·T6$^{+•}$

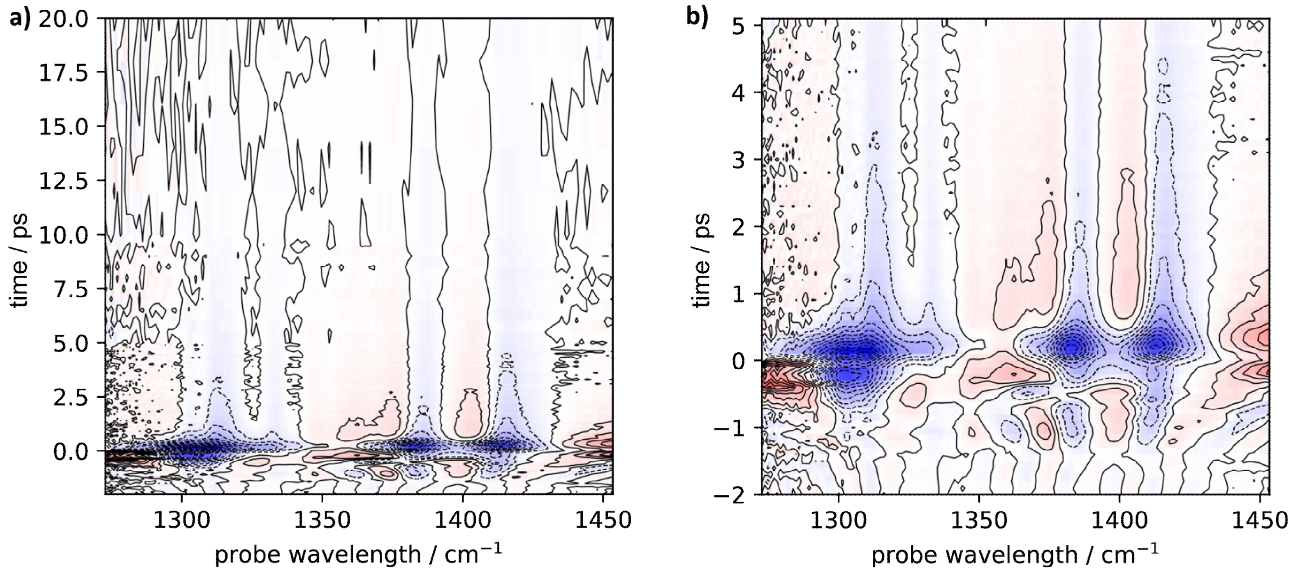

Fig. S19. A slice (ω_{pump} = 2061 cm^{-1}) of the 2D-IR LIFEtime data recorded for ***c*-P6·T6$^{+•}$** with **probe a** set to the region ~1250–1450 cm^{-1} showing (**a**) entire pump-probe delay series and (**b**) early time pump-probe delays. Red/blue correspond to positive(ESA)/negative(GSB) signals.

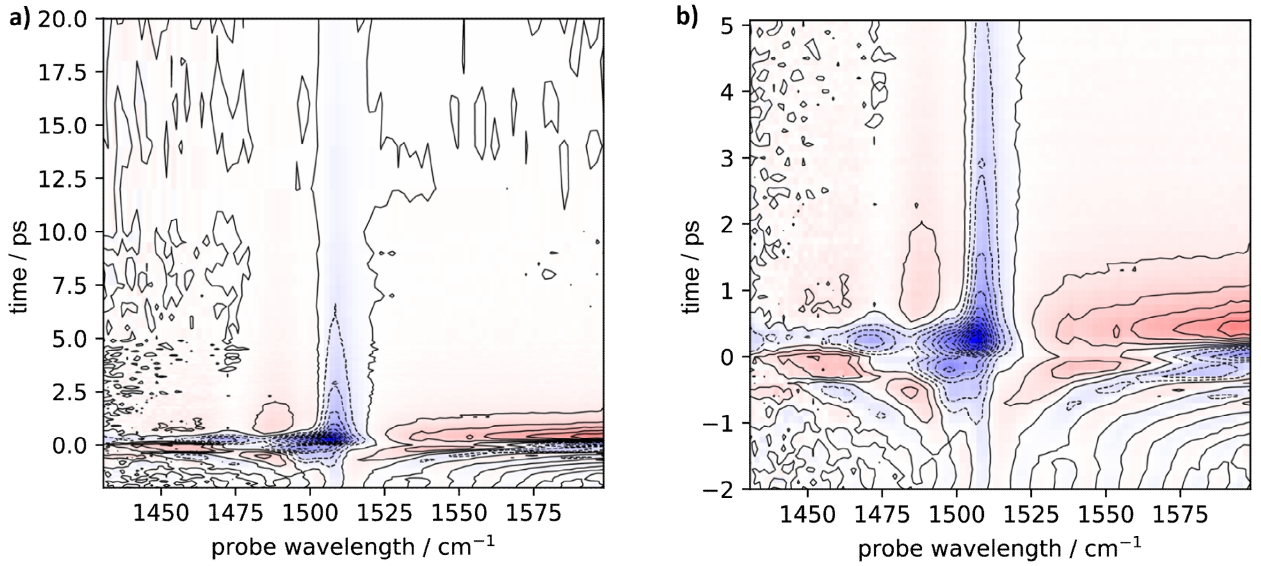

Fig. S20. A slice (ω_{pump} = 2061 cm^{-1}) of the 2D-IR LIFEtime data recorded for ***c*-P6•T6$^{+\bullet}$** with **probe a** set to the region ~1400–1600 cm^{-1} showing (**a**) entire pump-probe delay series and (**b**) early time pump-probe delays. Red/blue correspond to positive(ESA)/negative(GSB) signals.

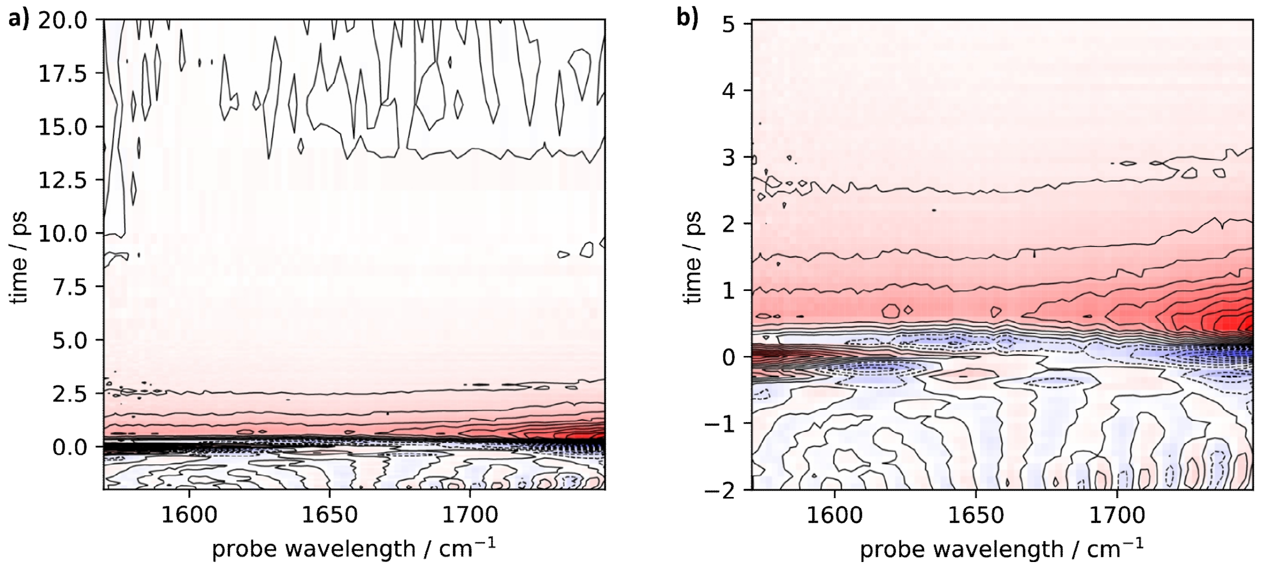

Fig. S21. A slice (ω_{pump} = 2061 cm^{-1}) of the 2D-IR LIFEtime data recorded for ***c*-P6·T6$^{+\bullet}$** with **probe a** set to the region ~1550–1750 cm^{-1} showing (**a**) entire pump-probe delay series and (**b**) early time pump-probe delays. Red/blue correspond to positive(ESA)/negative(GSB) signals.

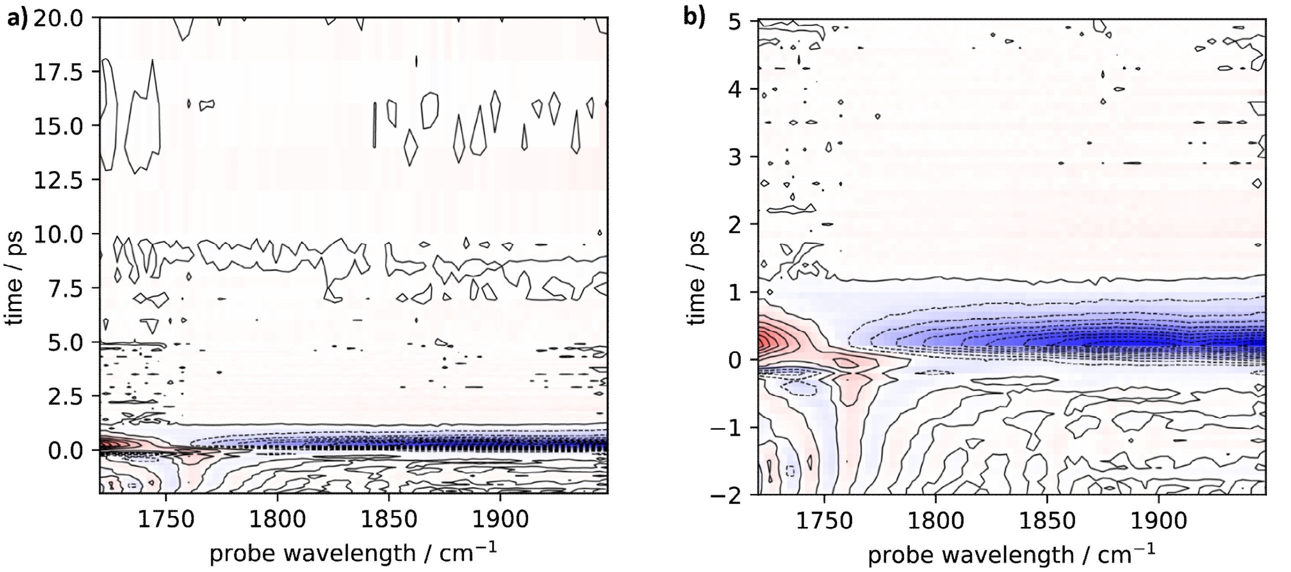

Fig. S22. A slice (ω_{pump} = 2061 cm^{-1}) of the 2D-IR LIFEtime data recorded for ***c*-P6·T6**$^{+\bullet}$ with **probe a** set to the region ~1700–1950 cm^{-1} showing (**a**) entire pump-probe delay series and (**b**) early time pump-probe delays. Red/blue correspond to positive(ESA)/negative(GSB) signals.

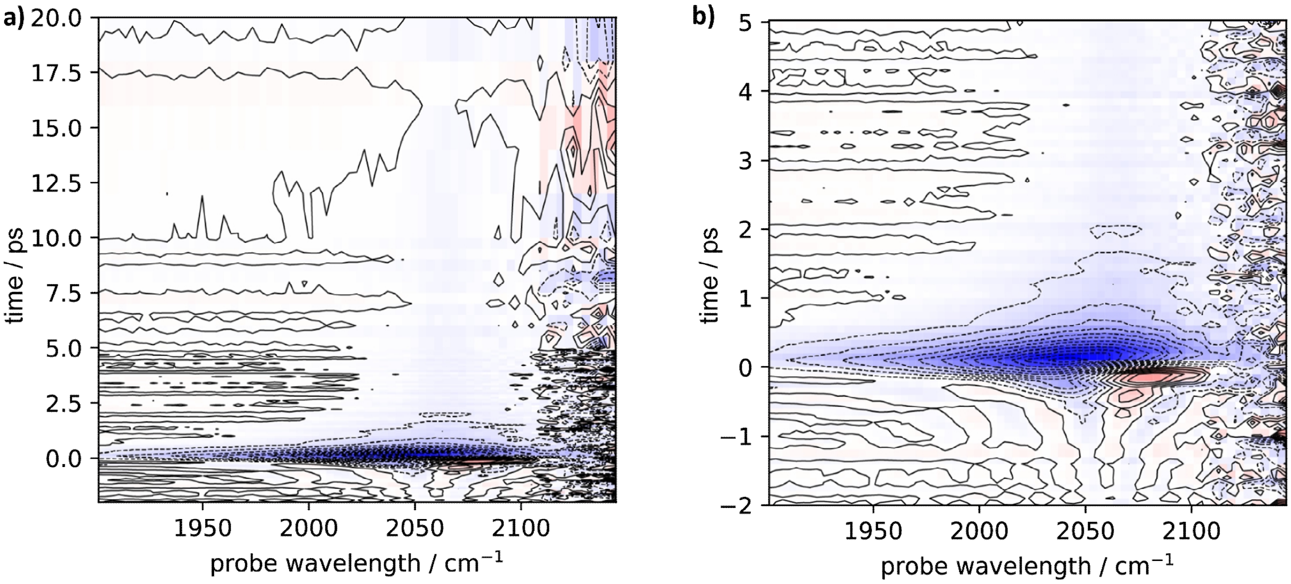

Fig. S23. A slice (ω_{pump} = 2061 cm^{-1}) of the 2D-IR LIFEtime data recorded for ***c*-P6·T6**$^{+\bullet}$ with **probe b** set to the region ~1900–2150 cm^{-1} showing (**a**) entire pump-probe delay series and (**b**) early time pump-probe delays. Red/blue correspond to positive(ESA)/negative(GSB) signals.

3 TRIR

3.1 Polarization-sensitive TRIR

Polarization-sensitive TRIR measurements were carried out as described in the main text. Time delays were randomized to minimize the effect of fluctuations in the sample concentration (see sample preparation section). The perpendicular, parallel and magic angle pump-probe orientations were recorded concurrently at each time delay by an automatic λ/2 plate. By keeping the averaging time low and performing more cycles, the effect of a change in the sample composition between measurements at different polarizations could be minimized.

Fig. S24 shows the control measurement of the anisotropy of a $W(CO)_6$ sample at ∼1980 cm^{-1}, finding good agreement with previously published data.[11]

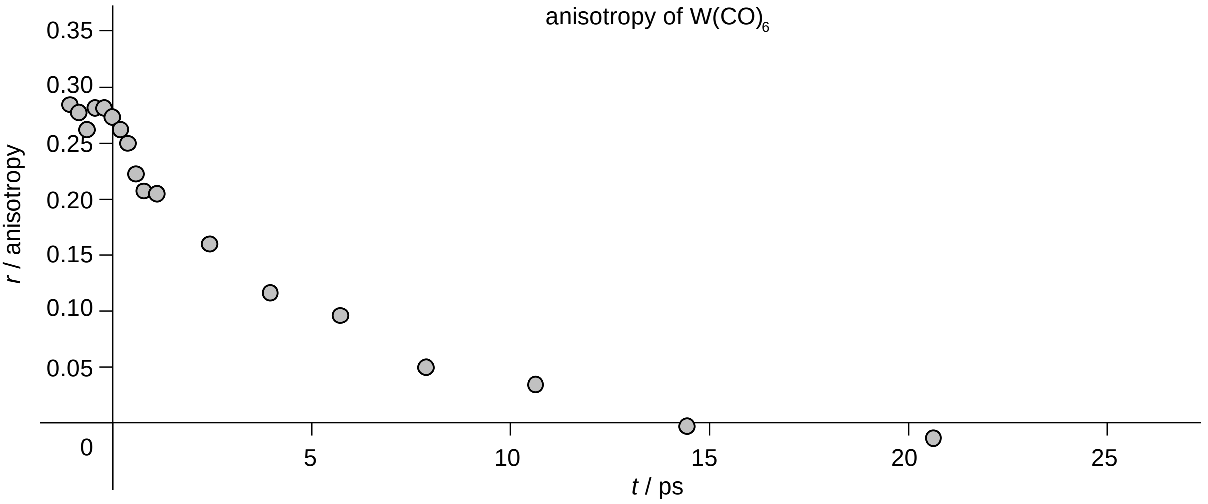

Fig. S24. Plot of anisotropy of $W(CO)_6$ (calculated using eq. 2, main text) measured in CD_2Cl_2, pumped and probed at ∼1980 cm^{-1} with variable probe delays.

The dependence of the pump-probe orientation was tested on $W(CO)_6$ at 0.1 ps probe delay by recording a spectrum at a range of pump-probe angles, in 10° increments, to confirm the correct behavior of the instrument setup. The relation between parallel, perpendicular and magic angle intensities is expected to follow equation S5.

$$\Delta A_{magic} = \frac{1}{3}(\Delta A_{\parallel} + 2 \cdot \Delta A_{\perp}) \qquad \text{(S5)}$$

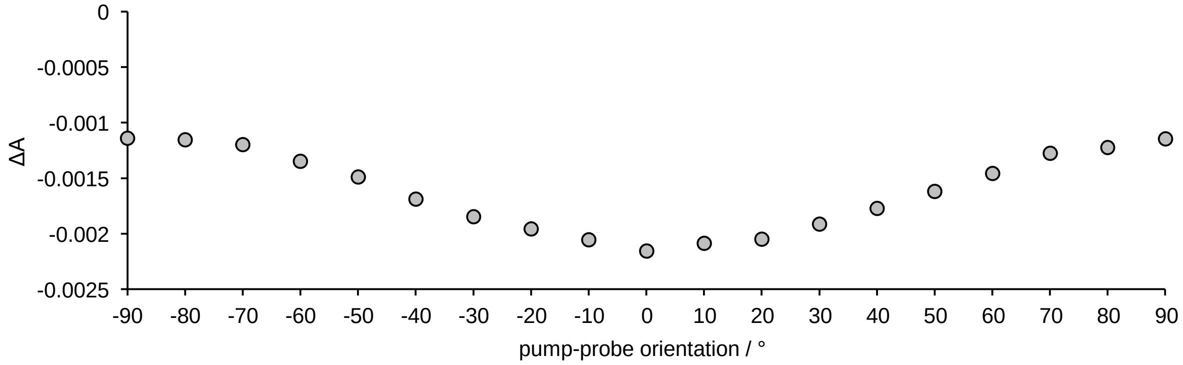

Fig. S25. GSB intensity of $W(CO)_6$ (delay time 0.1 ps) dependence on pump-probe orientation.

Values for parallel, magic angle and perpendicular orientation were −2.1555, −1.4186 and −1.139 mOD, respectively. Applying equation S5, the magic angle intensity is expected to be −1.478 mOD, which is close to the experimental value. Furthermore, TRIR was collected at the magic angle orientation for every anisotropy-analyzed TRIR spectra pumped at ~2050 and ~2500 cm^{-1} regions. As a representative example, the orientation-dependence of the GSB intensity of ***l*-P2$^{+\bullet}$**, pumped at ~2050 and probed at 1533 cm^{-1} is shown in **Fig. S26**.

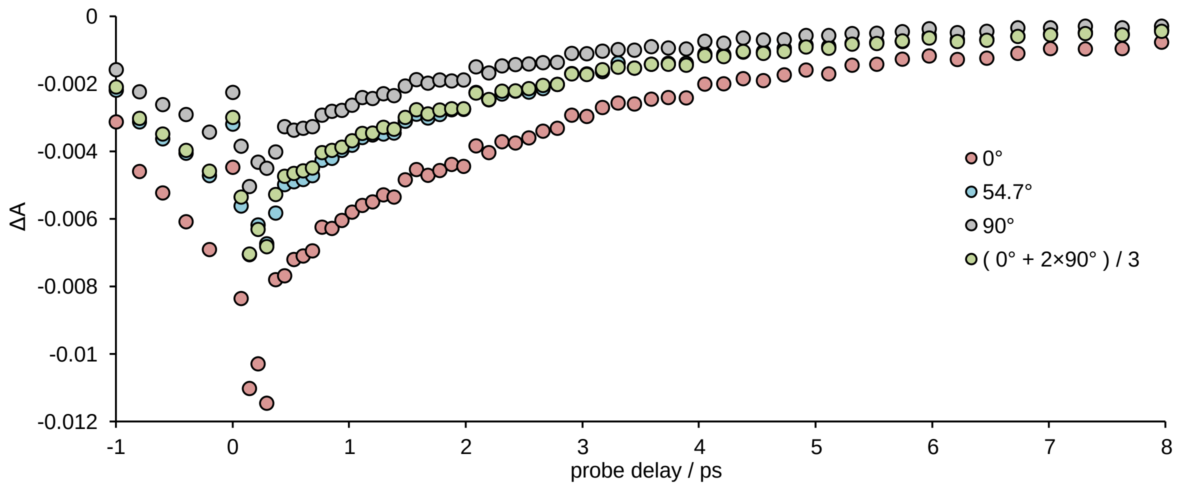

Fig. S26. GSB intensity of ***l*-P2$^{+\bullet}$** dependence on pump-probe orientation for ω_{pump} = 2050 cm^{-1} and ω_{probe} = 1533 cm^{-1}. Combination of parallel and perpendicular orientations (according to eq. S2) gives values congruent to experimentally observed magic angle data.

3.2 TRIR anisotropy analysis

Only certain TRIR regions were selected to plot anisotropy, due to the presence of discontinuities in regions where TRIR spectra cross or are close to the baseline (ΔA = 0). Practically, the anisotropy was analysed only in regions where real and distinguishable peaks arose. Within each region, only those signals at each time delay which had an intensity above an arbitrarilly chosen signal-to-noise level (typically signal intensities at 0.8 ps > 0.3–1 mOD) were used for the anisotropy analysis.

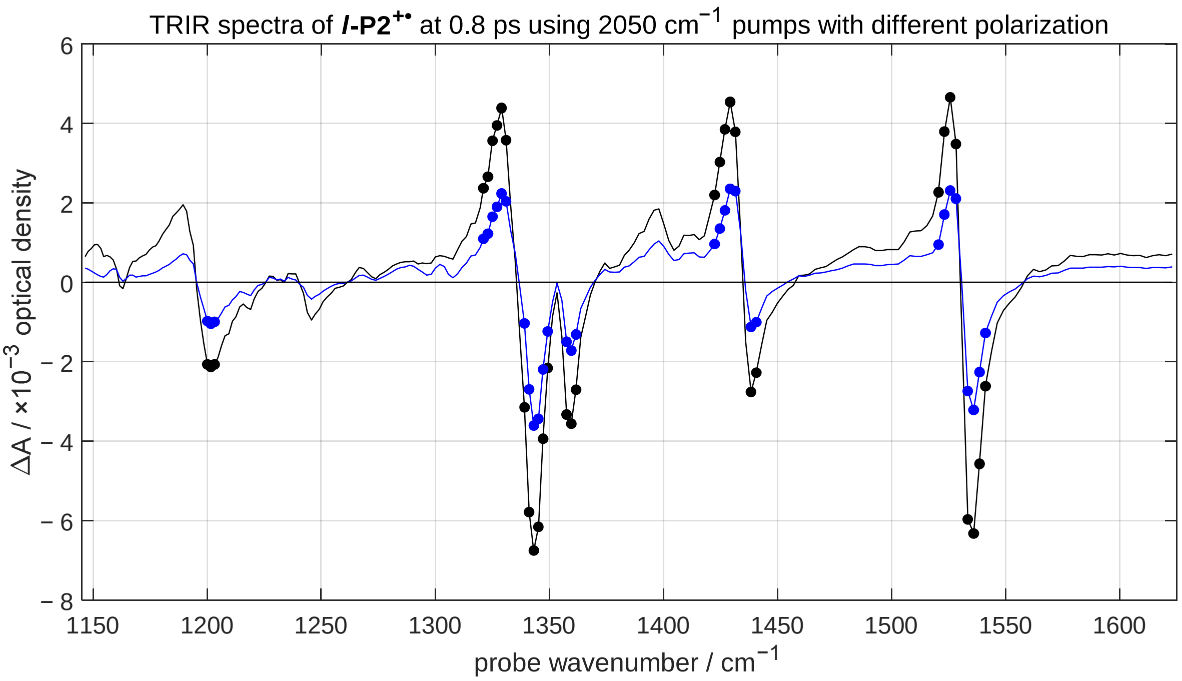

Fig. S27. TRIR spectra of ***l*-P2$^{+•}$** pumped at 2050 cm^{-1} with parallel (black) and perpendicular (blue) orientation to the probe delayed by 0.8 ps. The positions indicated by filled circles were used for the anisotropy analysis.

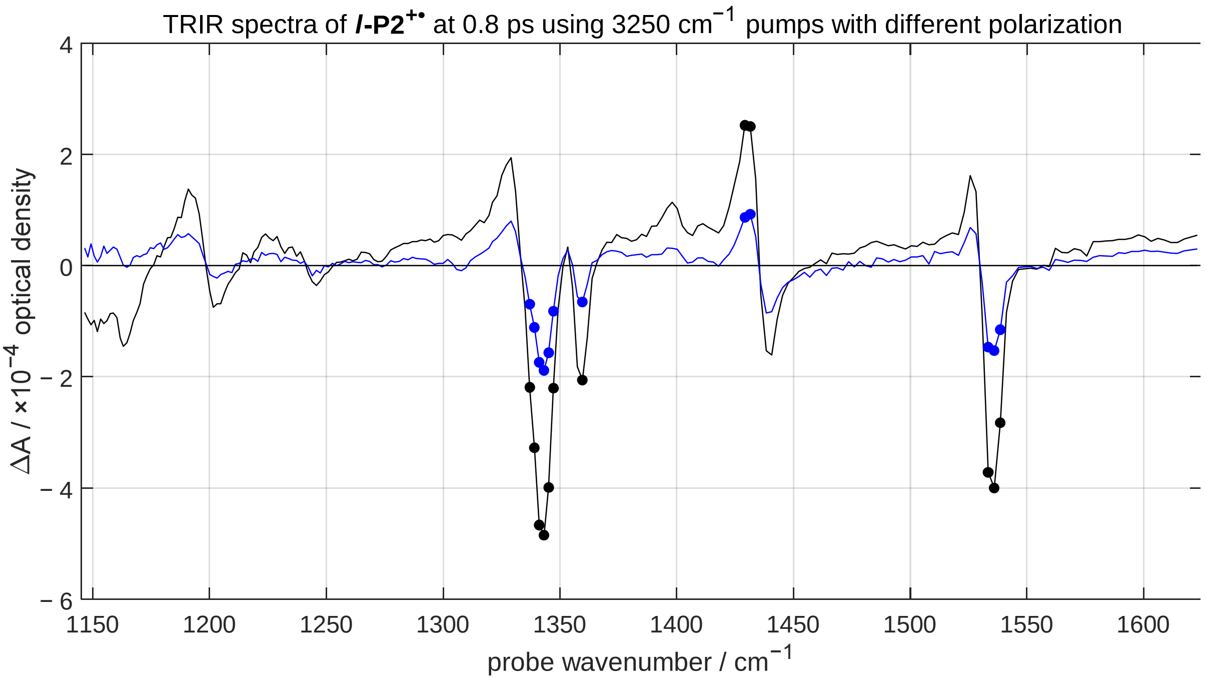

Fig. S28. TRIR spectra of ***l*-P2$^{+•}$** pumped at 3250 cm^{-1} with parallel (black) and perpendicular (blue) orientation to the probe delayed by 0.8 ps. The positions indicated by filled circles were used for the anisotropy analysis.

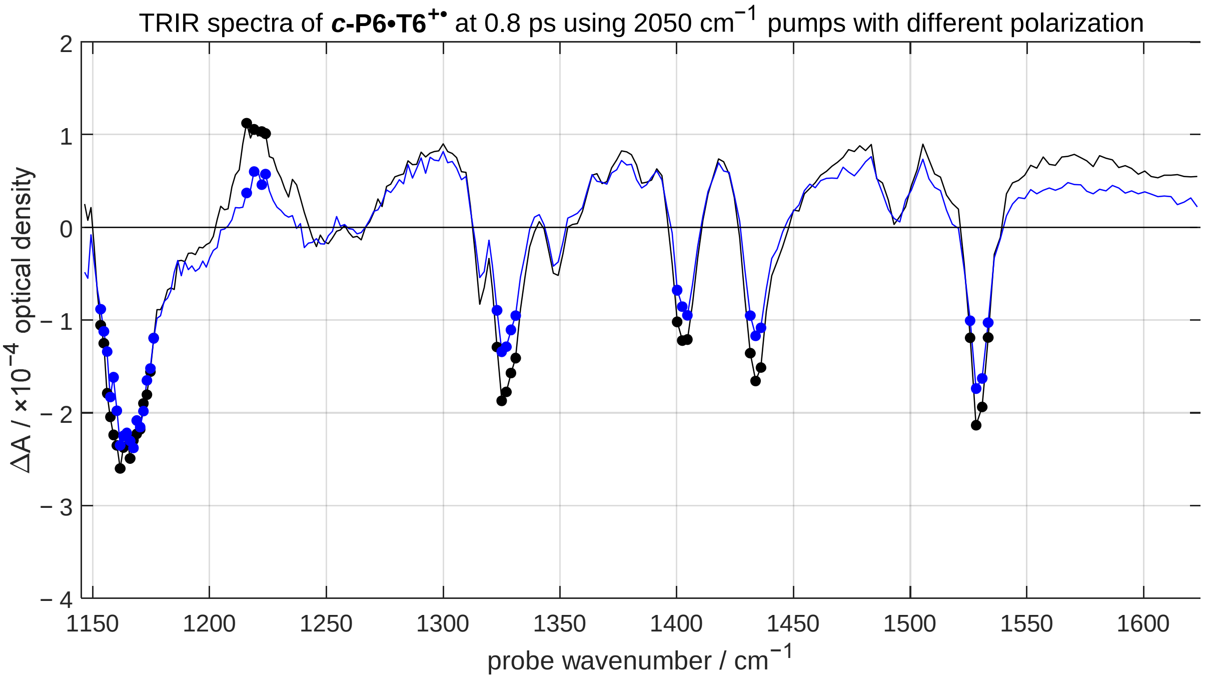

Fig. S29. TRIR spectra of ***c*-P6·T6$^{+\bullet}$** pumped at 2050 cm^{-1} with parallel (black) and perpendicular (blue) orientation to the probe delayed by 0.8 ps. The positions indicated by filled circles were used for the anisotropy analysis.

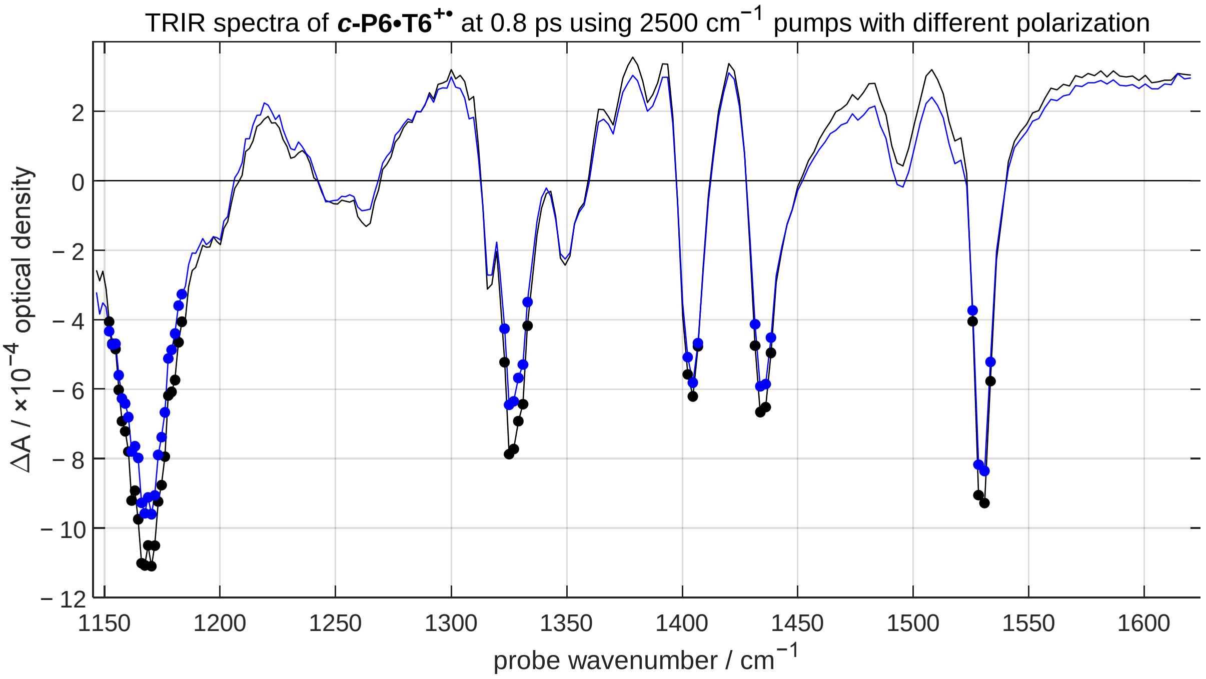

Fig. S30. TRIR spectra of ***c*-P6·T6$^{+\bullet}$** pumped at 2500 cm^{-1} with parallel (black) and perpendicular (blue) orientation to the probe delayed by 0.8 ps. The positions indicated by filled circles were used for the anisotropy analysis.

The plots of anisotropy in **Figs. S31, 32** demonstrate the necessity of enforcing a signal-noise threshold.

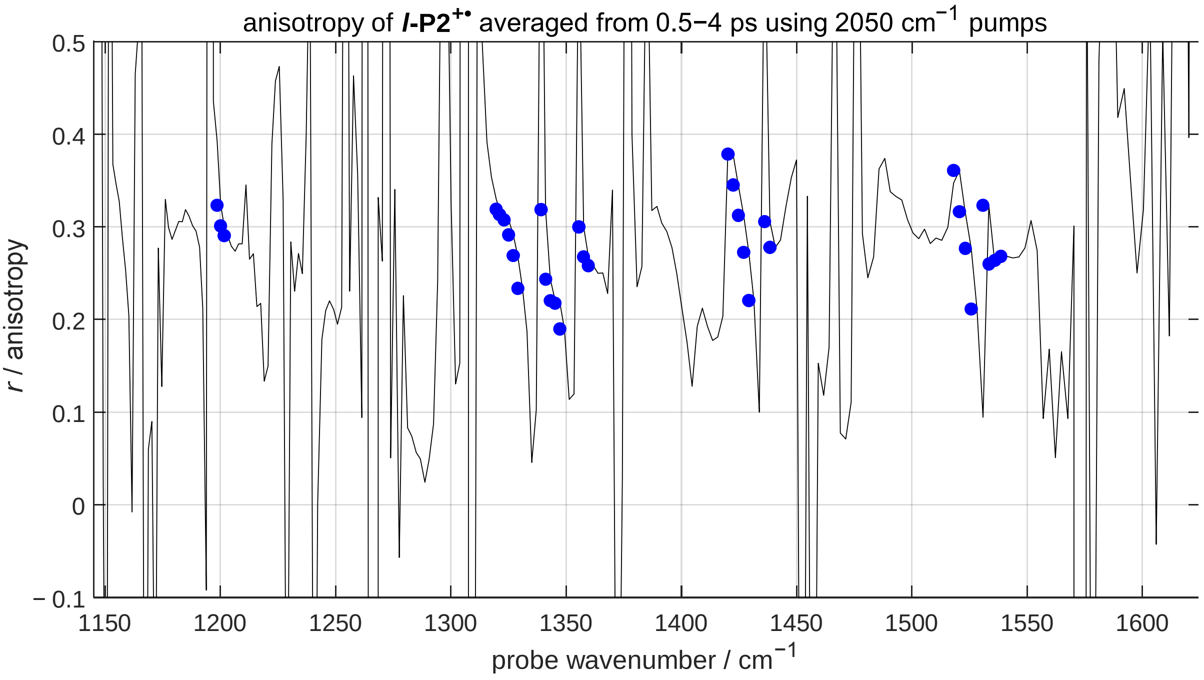

Fig. S31. Plot of anisotropy of ***l*-P2**$^{+\bullet}$ pumped at 2050 cm^{-1}. Values were averaged from 0.5–4 ps. Circles indicate points with signal-to-noise above the chosen threshold (c.f. **Fig. S27**)

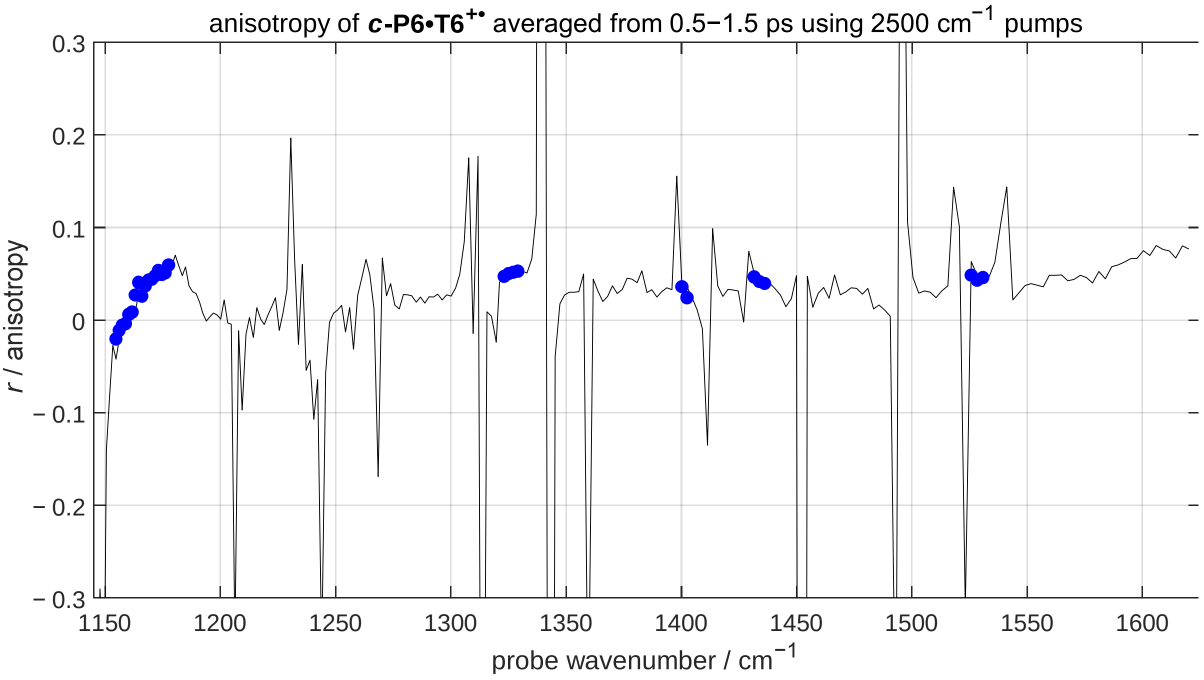

Fig. S32. Plot of anisotropy of ***c*-P6·T6**$^{+\bullet}$ pumped at 2050 cm^{-1}. Values were averaged from 0.5–1.5 ps. Circles indicate points with signal-to-noise above the chosen threshold (c.f. **Fig. S28**).

3.3 Kinetic analyses of TRIR spectra

TRIR data with the best time resolution were acquired using **ULTRA** system. Probe windows typically cover ~300 cm^{-1} and multiple windows were thus needed to cover the whole region of interest (~1100–2200 cm^{-1}) for both ***l*-P2$^{+\cdot}$** and ***c*-P6·T6$^{+\bullet}$**. Analysis of the kinetic profiles shows general features. Often, the whole set of ESA and GSB decay/recover with similar rates. Principal component analysis *via* Singular Value Decomposition (SVD) method implemented in software SurfaceXplorer (verison 4.3.0) was used to assess number of 'real' components and to obtain the population profiles of the main components. One component often represented well the general features of the whole set of ESA/GSB. The second component often described well the high intense and fast GSB recoveries. Fit of exponentional functions to the component population was performed using OriginPro 2017 software, providing kinetic profiles with the noise reduced by the SVD procedure. Nevertheless, individual peaks from ESA and GSB were also analyzed individually.

Such kinteic analysis did not strictly follow the proposed model with three components, based on detailed analysis of 2D-IR data. The fits of principal components populations fail to well describe the three-component model, most often not recovering the slowest component (which presence is however evident from the 2D plots of TRIR data) and often only a two-component system was assumed. TRIR kinetic analysis thus serve mainly to show the general similarity with 2D-IR-based observations of the presence of both fast and slow components in the GSB recovery and an apparently slower, monoexponentially decaying ESA.

3.3.1 *l*-P2$^{+\bullet}$ TRIR kinetic analysis

Kinetic analyses of TRIR spectra (principal component population or individual pixels) were performed by fit of the mono-exponential function, eqn. S6:

$$y = A \cdot e^{-\frac{t}{\tau}} + y_0 \qquad \text{(S6)}$$

or where needed bi-exponential function, eqn. S7:

$$y = A_1 \cdot e^{-\frac{t}{\tau_1}} + A_2 \cdot e^{-\frac{t}{\tau_2}} + y_0 \qquad \text{(S7)}$$

where A is pre-exponential factor weighing the component, y_0 is offset and τ is a lifetime.

3.3.1.1 *l*-P2$^{+\bullet}$ pump 3000 cm^{-1}, probe 1100–1400 cm^{-1}

In the probe region 1100–1400 cm^{-1} (**Fig. S33** and **Fig. S34**) two GSB and one ESA are observed. The second ESA is probably overwhelmed by more intense GSB. At early times, only strong GSB is observed. At delay ~0.1–0.2 ps ESA appear.

Spectrum obtained from principal component analysis assuming one component is very similar to the TRIR at late delay times. Fit of the principal component population assuming bi-exponential decay gives lifetimes τ_1 = 4.1 ± 1.5 ps and τ_2 = 0.31 ± 0.04 ps and pre-exponential factors A_1 = 0.28 and A_2 = 0.07.

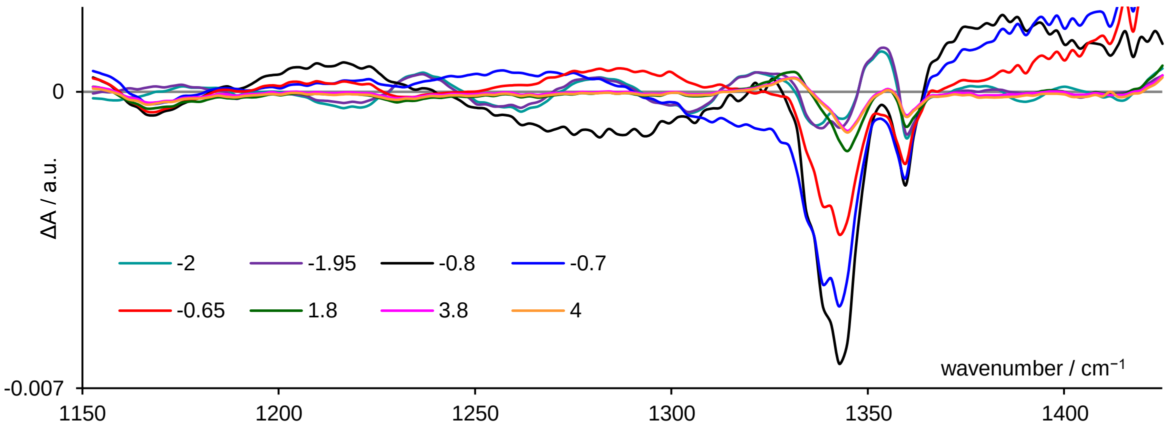

Fig. S33. TRIR of ***l*-P2$^{+•}$** pumped at 3000 cm^{-1} and probed in the fingerprint region at various probe time delays (in ps).

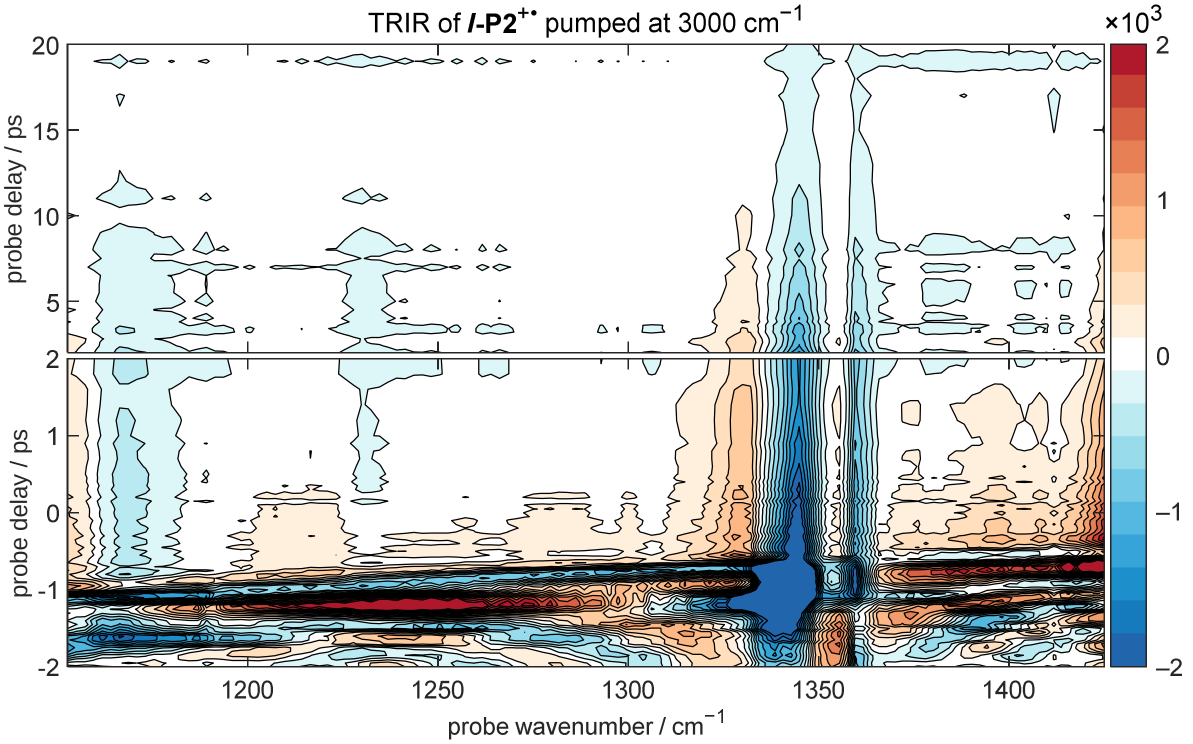

Fig. S34. Contour plot of TRIR spectra of ***l*-P2$^{+•}$** pumped at ~3000 cm^{-1} and probed at 1100–1400 cm^{-1}.

3.3.1.2 *l*-P2$^{+\cdot}$ pump 3000 cm^{-1}, probe 1400–1600 cm^{-1}

In the probe region 1400–1600 cm^{-1} (**Fig. S35**) at early times, strong GSB and broad positive transient features are observed. These vanish within 3 ps, followed with standard (~1:1 intensity) ESA/GSB corresponding to the two probed IRAV modes decaying relatively slowly.

Spectrum obtained from principal component analysis assuming one component is very similar to the TRIR at late delay times. Fit of the principal component population assuming bi-exponential decay gives lifetimes τ_1 = 4.4 ± 3.2 ps and τ_2 = 0.5 ± 0.1 ps and pre-exponential factors A_1 = 0.18 and A_2 = 0.09.

It is notable that for the ESA decays, a mono-exponential function provides a good fit, whereas for GSB recoveries, it is necessary to use a bi-exponential function.

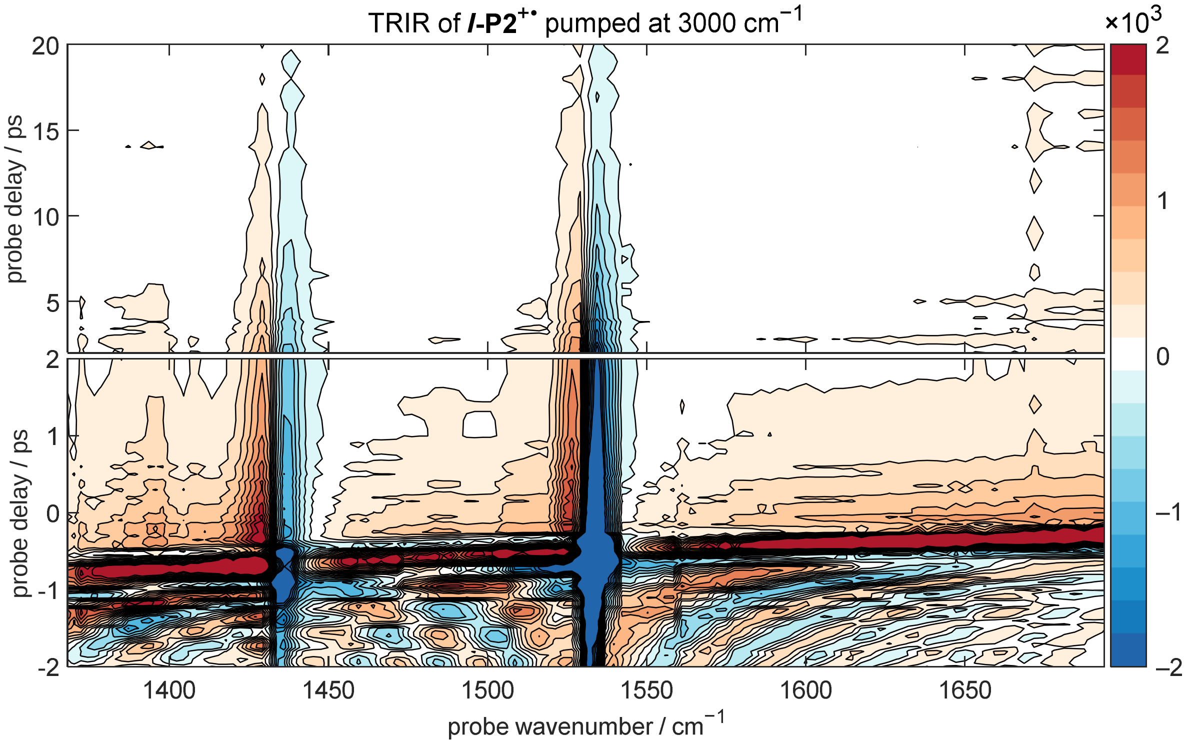

Fig. S35. Contour plot of TRIR spectra of ***l*-P2$^{+•}$** pumped at ~3000 cm^{-1} and probed in the fingerprint region.

	IRF / ps	τ_1 / ps	A_1	τ_2 / ps	A_2
ESA 1328 cm^{-1}	0.1	3 ± 5	0.001	---	---
GSB 1345 cm^{-1}	0.5	6 ± 3	0.0023	0.2 ± 0.3	0.097
GSB 1360 cm^{-1}	0.27	8 ± 5	0.0012	0.2 ± 0.3	0.006
ESA 1429 cm^{-1}	0.1	2 ± 2	0.00242	---	---
GSB 1438 cm^{-1}	0.1 fixed	9 ± 21	0.001	1 ± 1	0.0016
ESA 1526 cm^{-1}	0.1	5 ± 4	0.00181	---	---
GSB 1534 cm^{-1}	0.1 fixed	9 ± 32	0.002	1.3 ± 1.8	0.0065

Table S7: Summary of fitted parameters (particularly lifetimes) obtained from fits for each ESA, GSB and of TRIR of ***l*-P2$^{+•}$** pumped at ~3000 cm^{-1} assuming either mono- or bi-exponential decay. Parameters are defined according to equations S6 or S7.

3.3.1.3 *l*-P2$^{+\cdot}$ pump 3000 cm^{-1}, probe 2050 cm^{-1}

In the probed region ~2050 cm^{-1} one set of GSB/ESA is observed (**Fig. S36** and **Fig. S37**). Two unusual distinct features were also observed: Very intense fast decaying (~0.5 ps) GSB and positive offset disappearing in ~2 ps. The latter is assignable as a broad electronic transient absorption. The data were not possible to fit with a global model.

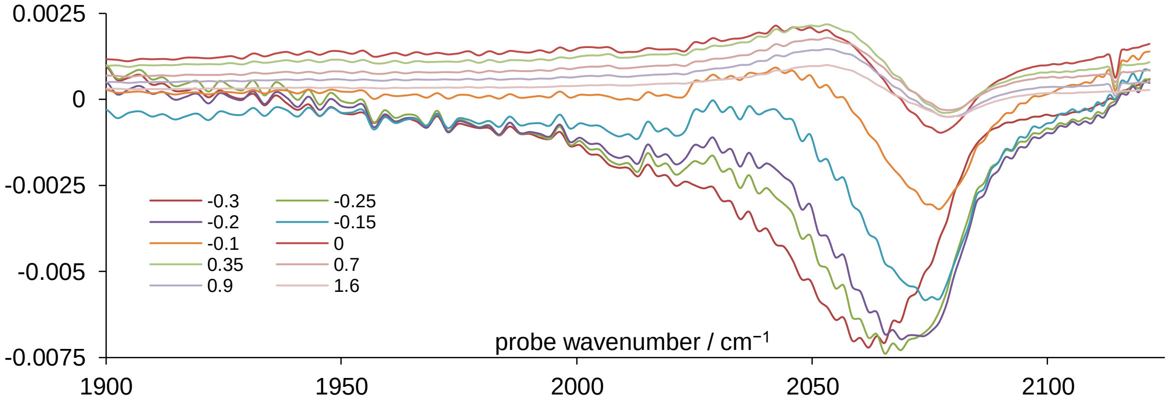

Fig. S36. TRIR of ***l*-P2$^{+\bullet}$**, pumped at 3000 cm^{-1} and probed in ~2050 cm^{-1} region at various delays.

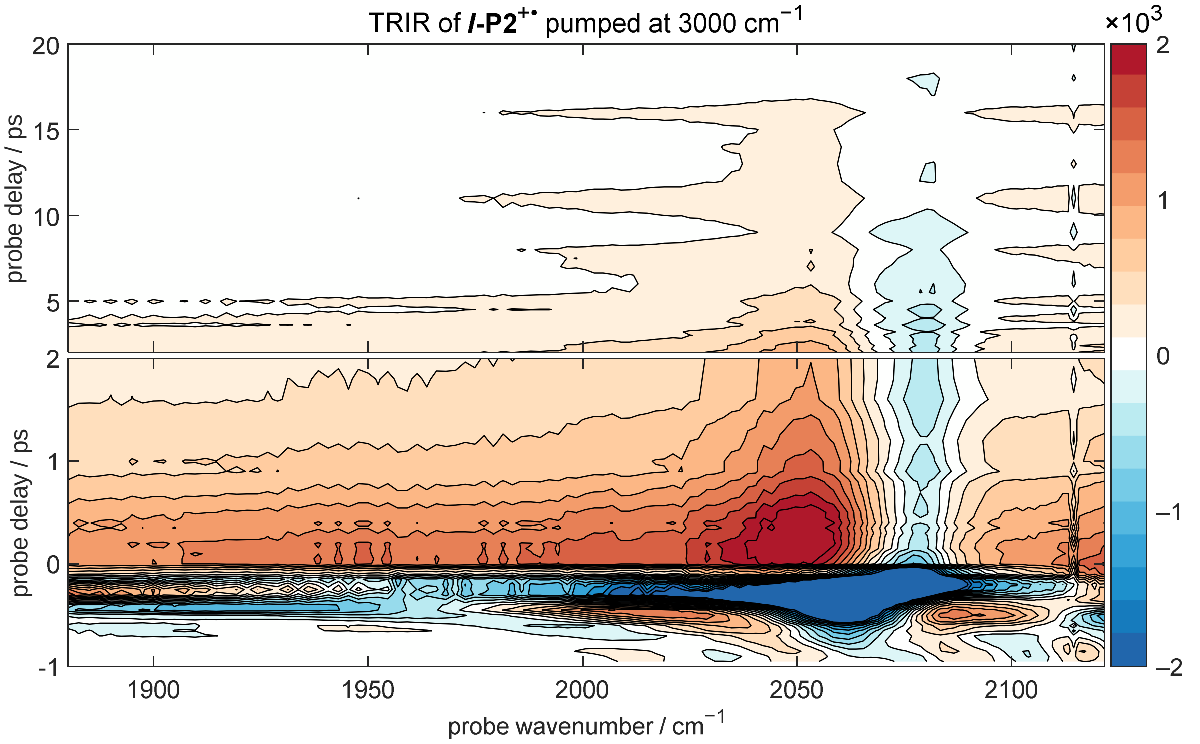

Fig. S37. Contour plot of TRIR spectra of ***l*-P2$^{+\bullet}$** pumped at ~3000 cm^{-1} and probed in the ~2050 cm^{-1} region.

The broad feature decays at the same rate across its entire energy range and can be fitted with monoexponential decay function Eq. S6. E.g. when probed at 1902 cm^{-1}, fitted lifetime is τ = 1.03± 0.04 ps. When probed at 1950 cm^{-1}, fitted lifetime is τ = 1.02± 0.04 ps;

3.3.1.4 *l*-P2$^{+•}$ pump 2050 cm^{-1}, probe 1280–1575 cm^{-1}

***l*-P2$^{+•}$** pumped at ~2050 cm^{-1} and probed in 1280–1575 cm^{-1} affords ESA/GSB peaks at the same positions as with the ~3000 cm^{-1} pump. With the pump at 2050 cm^{-1}, broad transient features were observed at early times, however, no very intense early time GSB was observed. The early broad positive signal vanishes within ~3 ps and classical ESA/GSB pairs (of ~1:1 ratio) decay slowly. Decay of the broad signal can be fitted (taking the intensity in-between sharp peaks) to mono-exponential decay with lifetime $\tau = 0.9 \pm 0.5$ ps. The whole probe window was not possible to fit globally. Individual fits are summarized in **Table S8**.

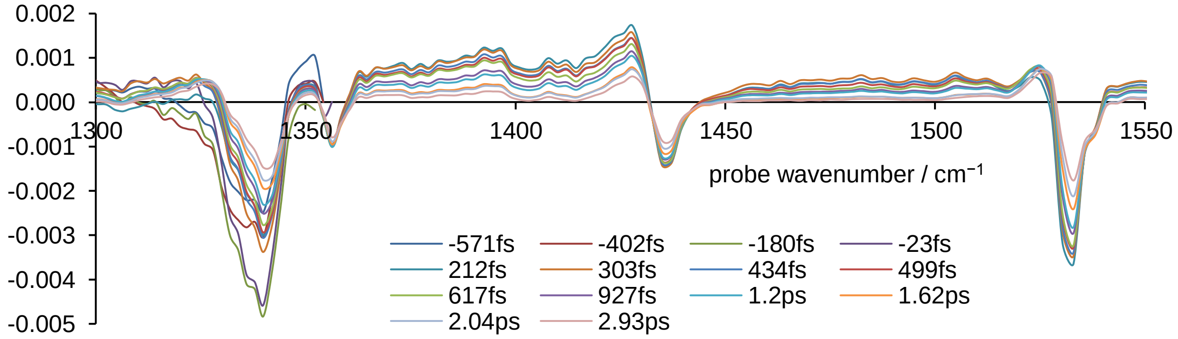

Fig. S38. TRIR spectra at selected probe delays of ***l*-P2$^{+•}$** pumped at ~2050 cm^{-1} and probed at 1280–1575 cm^{-1}.

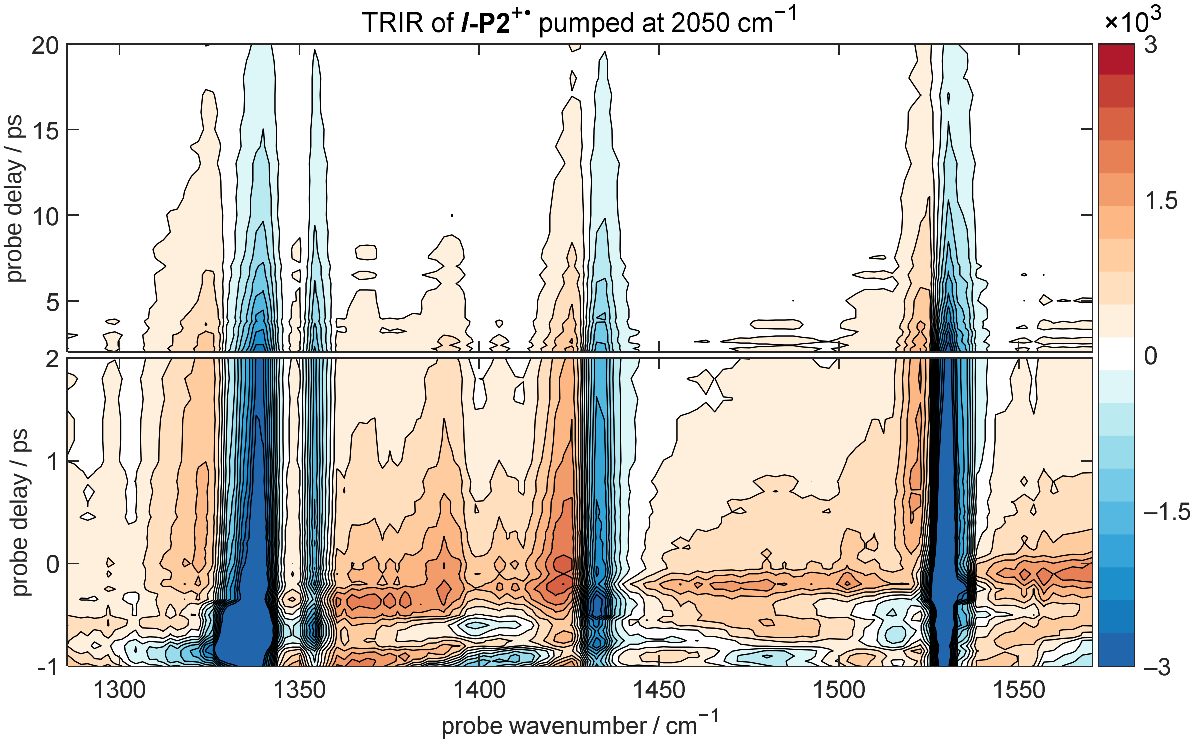

Fig. S39. Contour plot of TRIR spectra of ***l*-P2$^{+•}$** pumped at ~2050 cm^{-1} and probed at 1280–1575 cm^{-1}.

	IRF / ps	τ_1 / ps	A_1	τ_2 / ps	A_2
ESA 1322 cm^{-1}	0.7	5 ± 1	0.0005	---	---
GSB 1340 cm^{-1}	0.4	4 ± 1	0.005	0.2	2.2
GSB 1356 cm^{-1}	2.0	7 ± 4	0.001	---	---
ESA 1403 cm^{-1}	0.9	1 ± 1	0.002	---	---
ESA 1428 cm^{-1}	0.4	2 ± 1	0.004	---	---
GSB 1437 cm^{-1}	0.1	6 ± 1	0.002	---	---
ESA 1522 cm^{-1}	0.4	5.6 ± 0.2	0.001	---	---
GSB 1533 cm^{-1}	0.4	3.3 ± 0.3	0.006	---	---

Table S8. Summary of fitted parameters obtained from fits for each ESA, GSB of the ***l*-P2$^{+\bullet}$**, pumped at ~2050 cm^{-1}. Fit were assumining either mono- or bi-exponentional function with IRF function, as implemented in SurfaceXplorer.

3.3.1.5 *l*-P2$^{+\bullet}$ pump ~2050 cm^{-1}, probe ~2050 cm^{-1}

Pumping and probing region at ~2050 cm^{-1} provided expected pair of GSB/ESA (**Fig. S40**) similar to experiment with ~3000 cm^{-1} pump (**Fig. S40**). At early delay times, intense GSB and broad transient appears. Within 2 ps spectrum is transferred into classical GSB/ESA pair. Principal component analysis assuming one components (**Fig. S41b**) was fitted to bi-exponential function S7. Fitted halflifes were τ_1 = 0.81 ± 0.05 ps and τ_2 = 9.4 ± 11.4 ps, with preexponentional factors A_1 = 0.23 and A_2 = 0.04.

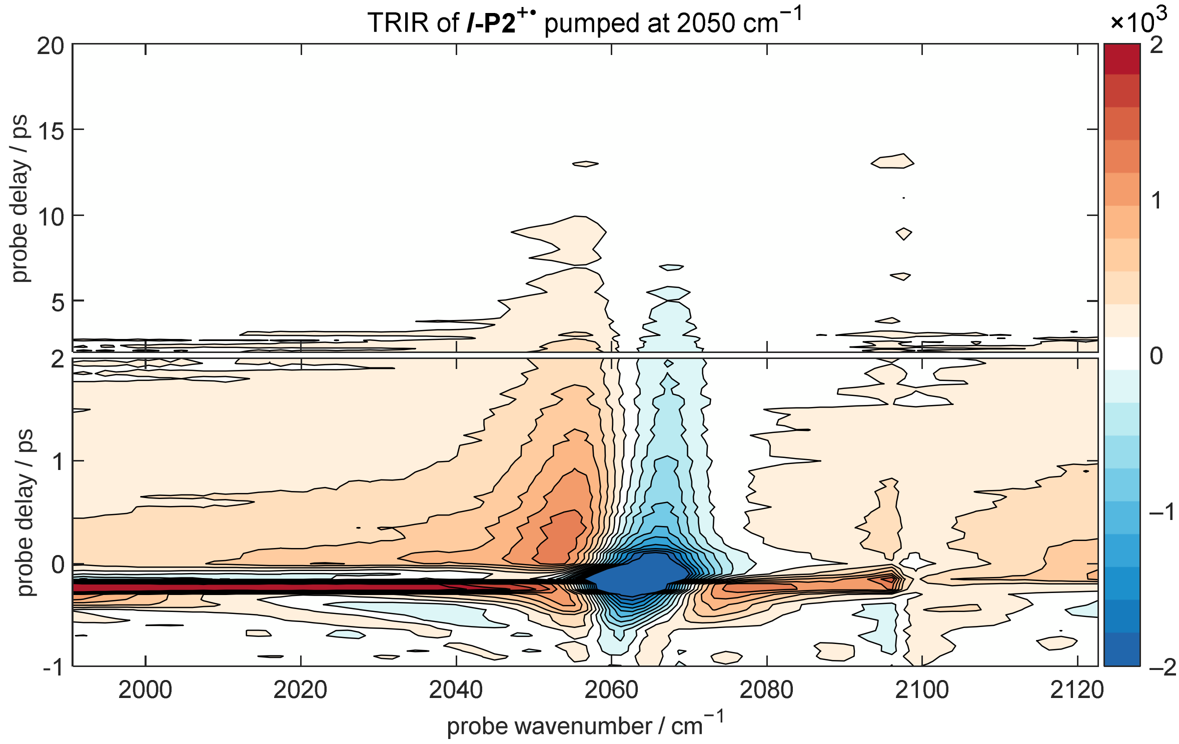

Fig. S40. Contour plot of TRIR spectra of ***l*-P2$^{+\bullet}$** pumped at ~2050 cm^{-1} and probed in the ~2050 cm^{-1} region.

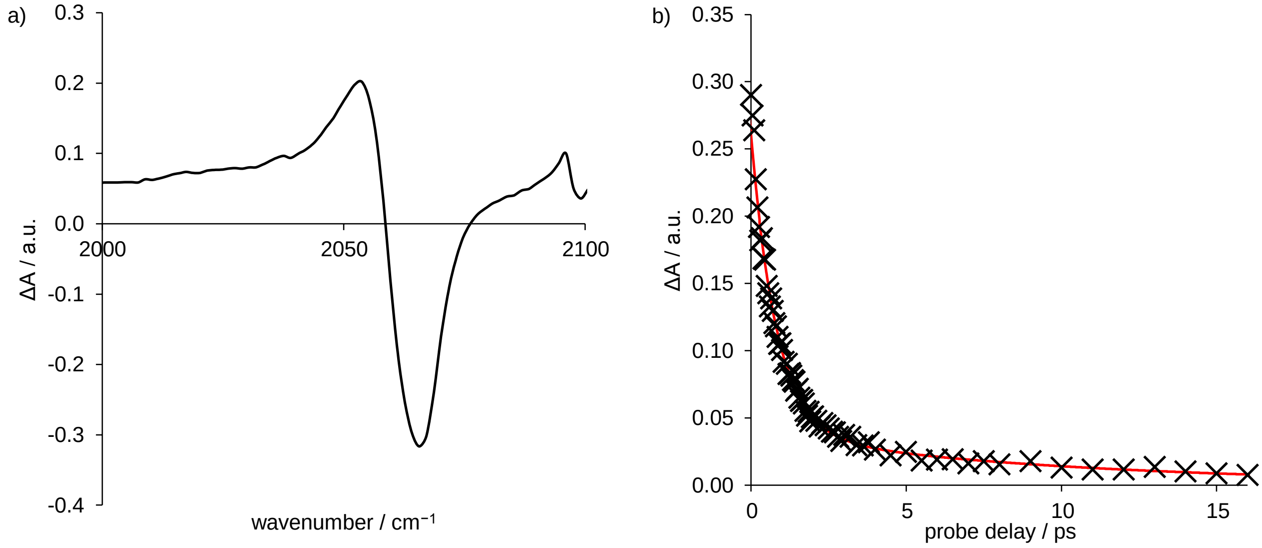

Fig. S41. a) TRIR spectrum of ***l*****-P2**$^{+\bullet}$, pumped and probed at ~2050 cm^{-1} region, as exctracted from the principal component analysis assuming one component. b) Population of the component over over time, fitted with bi-exponential function (S7). Fitted values were y_0 = 0.001 ± 0.02, A_1 = 0.23, τ_1 = 0.81 ± 0.05 ps, A_2 = 0.04, τ_2 = 9.4 ± 11.4 ps.

3.3.2 *c*-P6·T6$^{+•}$ TRIR kinetic analysis

3.3.2.1 *c*-P6·T6$^{+•}$ pump 3000 cm^{-1}, probe 1150–1450 cm^{-1} and 1350–1700 cm^{-1}

Pumping at ~3000 cm^{-1} and probing in the fingerprint region of ***c*-P6·T6$^{+•}$** yields a positively offset series of GSB/ESA signals for every IRAV (**Fig. S42** and **Fig. S43**).

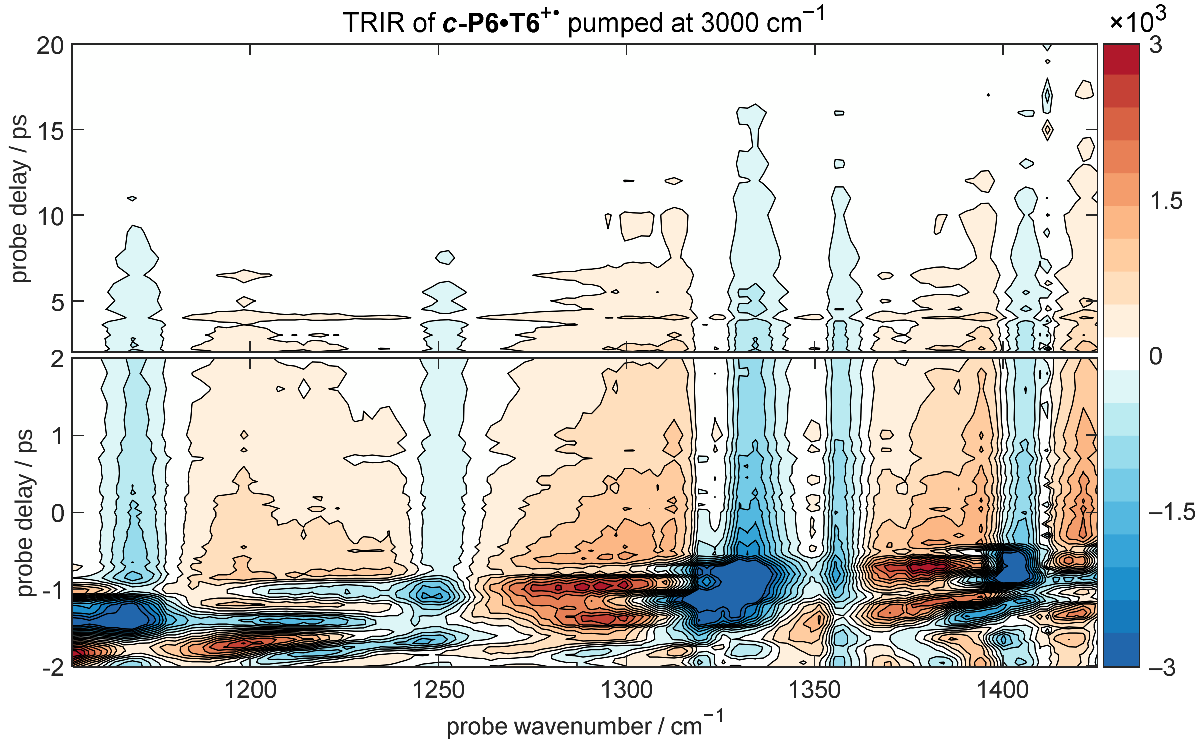

Fig. S42. Contour plot of TRIR spectra of ***c*-P6·T6$^{+•}$** pumped at ~3000 cm^{-1} and probed in the 1150–1450 cm^{-1} region.

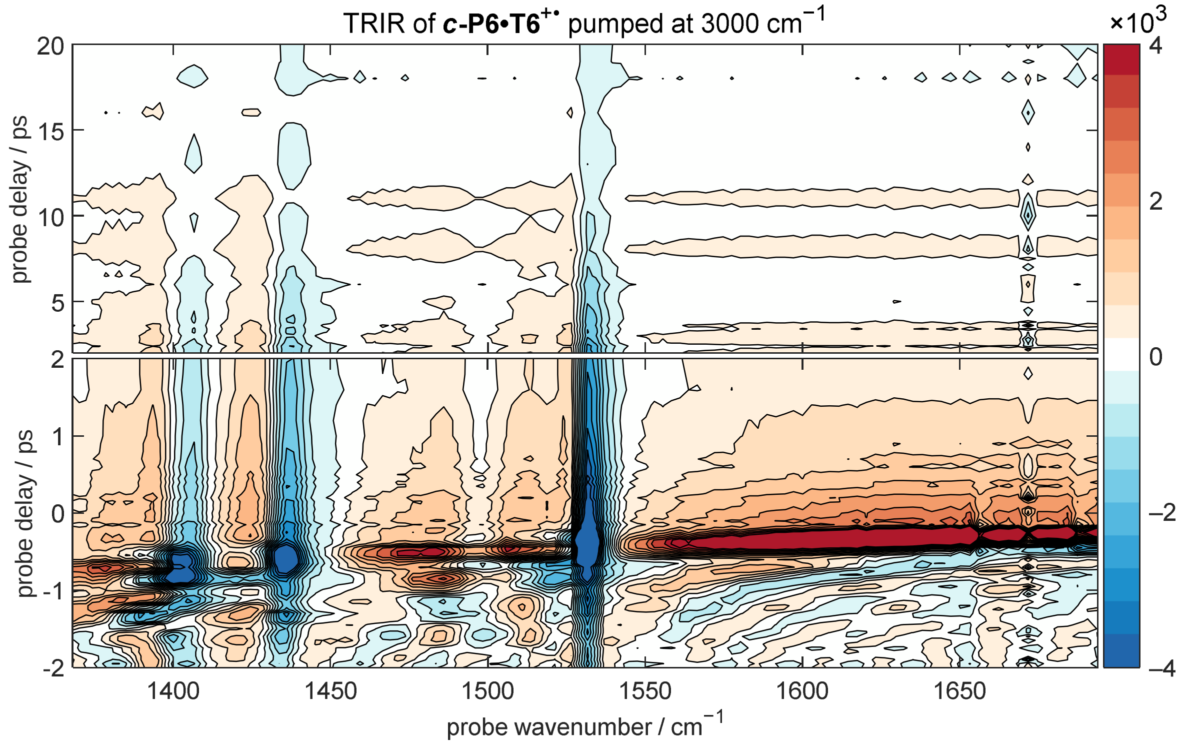

Fig. S43. Contour plot of TRIR spectra of ***c*-P6·T6$^{+•}$** pumped at ~3000 cm^{-1} and probed in the 1350–1700 cm^{-1} region.

Global fit of the probe window 1150–1450 cm^{-1} assuming one component provides spectrum similar to TRIR at later probe delay times (**Fig. S44**, black). The component population decay was fitted well with bi-exponential function with life-times τ_1 = 3.2 ± 2.2 ps and τ_2 = 0.04 ± 0.01 ps (**Fig. S45**, left). The second part of the fingerprint region (1350–1700 cm^{-1}) was treated in same way and provided principal component spectrum (**Fig. S44**, red) and comparable kinetic profile: τ_1 = 3.5 ± 2.4 ps and τ_2 = 0.04 ± 0.01 ps (**Fig. S45**, right).

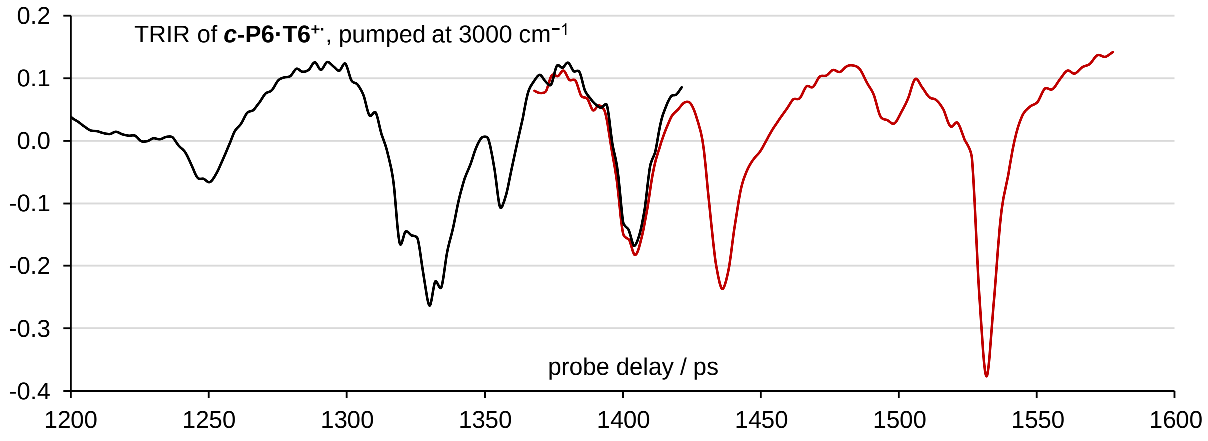

Fig. S44. Global fit of the TRIR spectra of ***c*-P6·T6$^{+\bullet}$** pumped at ~3000 cm^{-1} and probed in the fingerprint region composed of two windows 1150–1450 and 1350–1700 cm^{-1}.

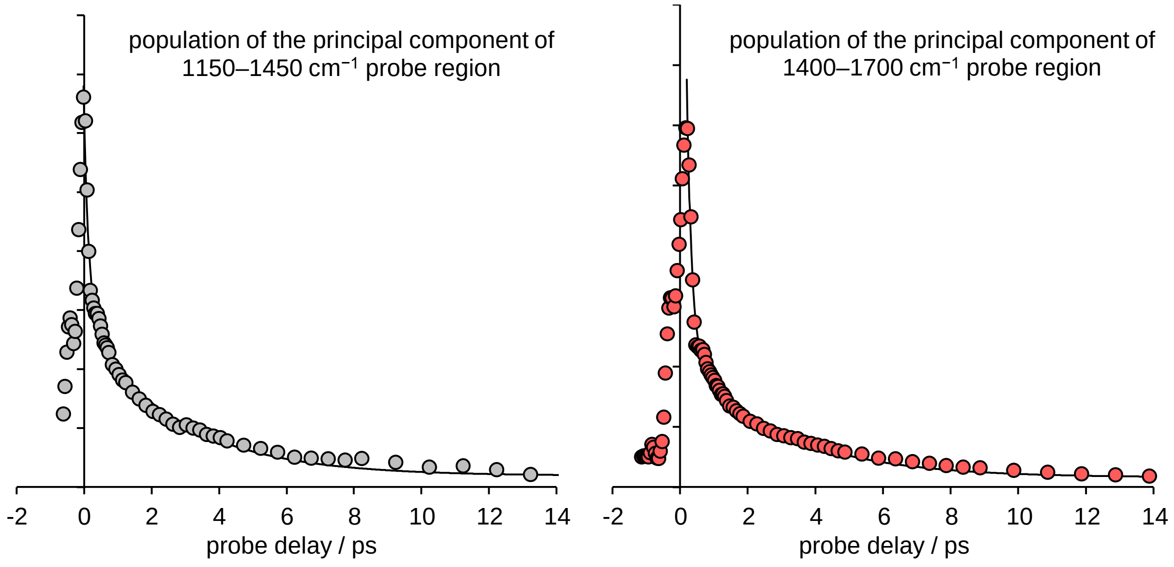

Fig. S45. Population of deconvoluted components over time, fitted withed with bi-exponential function. Fitted lifetimes were τ_1 = 0.16 ± 0.01 ps and $\boldsymbol{\tau_2}$ **= 2.7** ± 0.2 ps, with preexponentional factors A_1 = 0.176 ± 0.001 and A_2 = 0.129 ± 0.004 for the 1150–1450 cm^{-1} region. For the 1350–1700 cm^{-1} region, fitted lifetimes were $\boldsymbol{\tau_1}$ **= 0.15** ps and $\boldsymbol{\tau_2}$ **= 2.8** ps with preexponentional factors A_1 = 0.895 and A_2 = 0.11.

3.3.2.2 *c*-P6·T6$^{+•}$ pump ~3000 cm^{-1}, probe ~2050 cm^{-1}

Pumping at ~3000 cm^{-1} and probing in the ~2050 cm^{-1} region of ***c*-P6·T6**$^{+•}$ yields early time intense GSB, disappearing within 1 ps. At later time, classical GSB/ESA pair appears (**Fig. S46**).

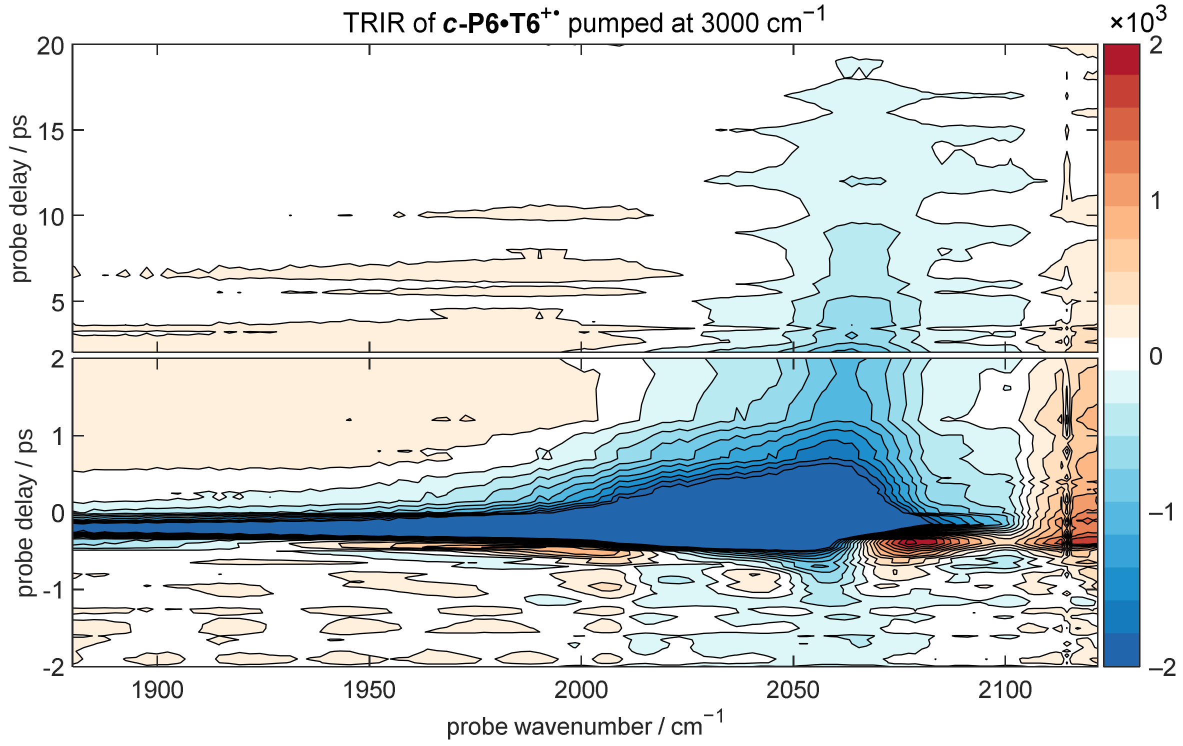

Fig. S46. Contour plot of TRIR spectra of ***c*-P6·T6**$^{+•}$ pumped at ~3000 cm^{-1} and probed in the ~2050 cm^{-1} region.

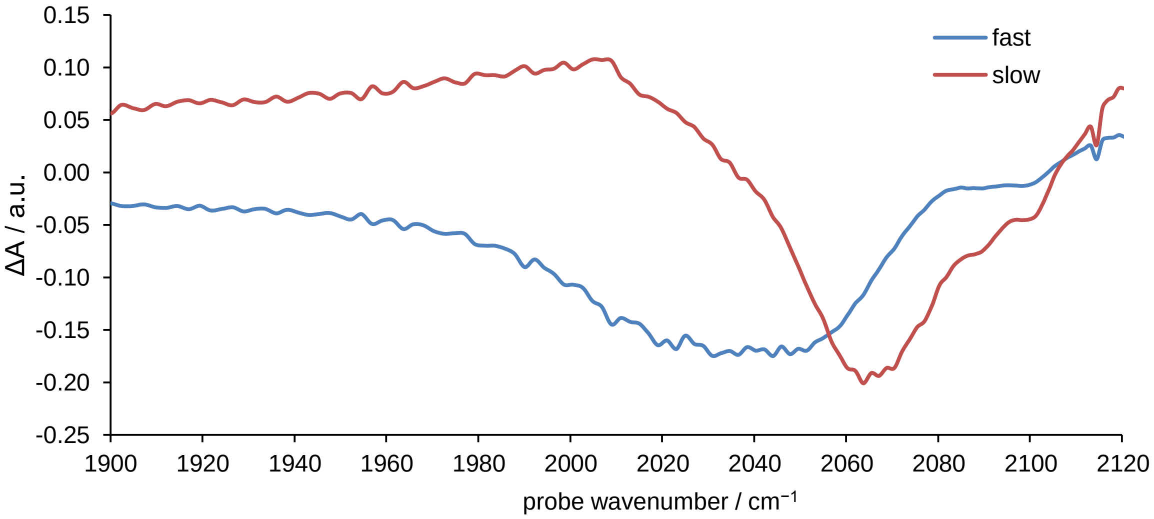

Fig. S47. Spectrum obtained prom principal component analysis assuming two components from the TRIR spectra of ***c*-P6·T6**$^{+•}$ pumped at ~3000 cm^{-1} and probed in the ~2050 cm^{-1} region.

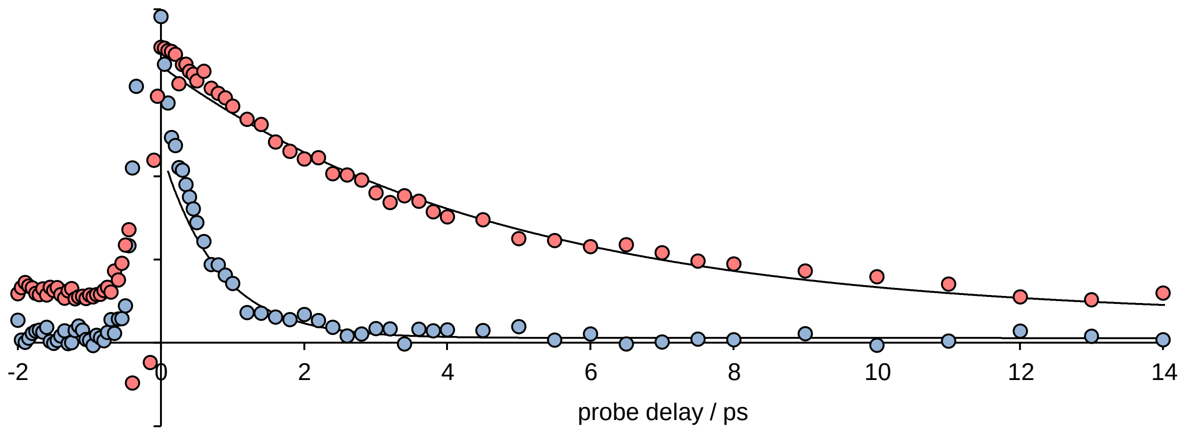

Fig. S48. Population of deconvoluted components from **Fig. S47** over time. Fitting with mono-exponentional decay function S6 provided lifetimes τ = 0.8 ± 0.1 ps and τ = 4.8 ± 0.2 ps for fast (blue) and slow (red) component, respectively.

3.3.2.3 *c*-P6·T6$^{+•}$ pump ~2050 cm^{-1}, probe 1150–1450 cm^{-1} and 1400–1700 cm^{-1}

Pumping at ~2050 cm^{-1} and probing in the fingerprint region of ***c*-P6·T6$^{+•}$** positively offset series of GSB/ESA signals for every IRAV, as expected (**Fig. S49** and **Fig. S50**).

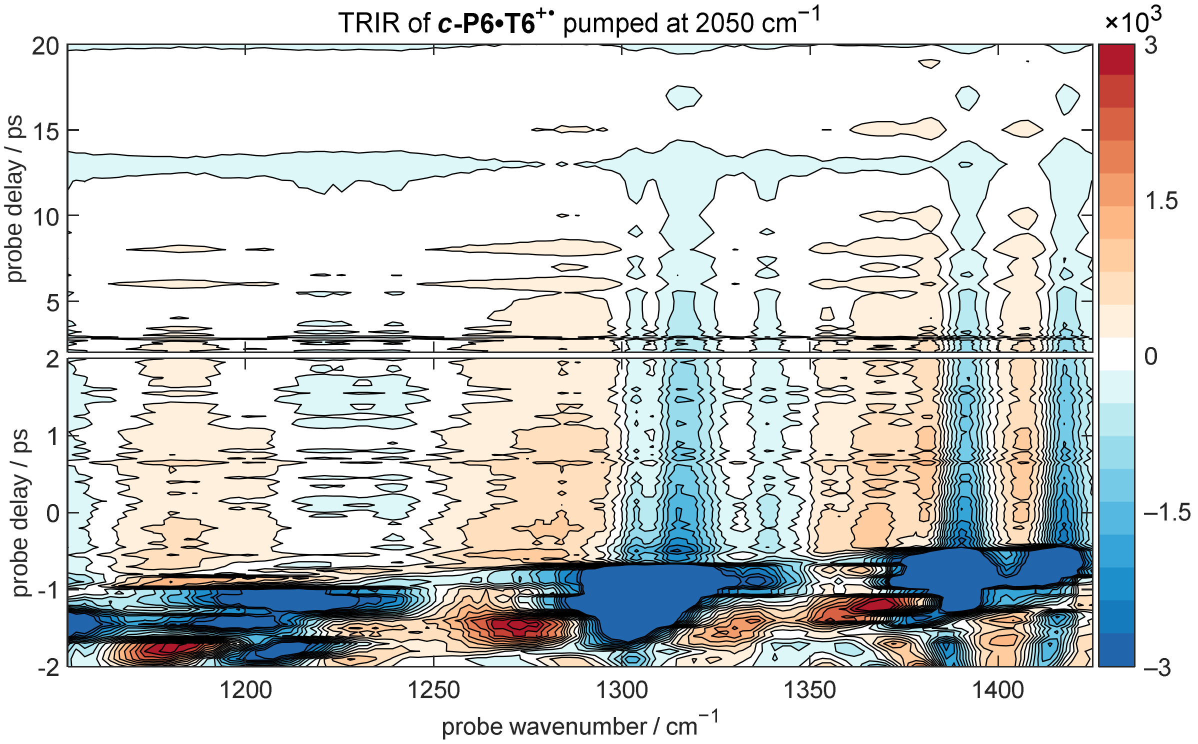

Fig. S49. Contour plot of TRIR spectra of ***c*-P6·T6$^{+•}$** pumped at ~2050 cm^{-1} and probed at 1150–1450 cm^{-1} region.

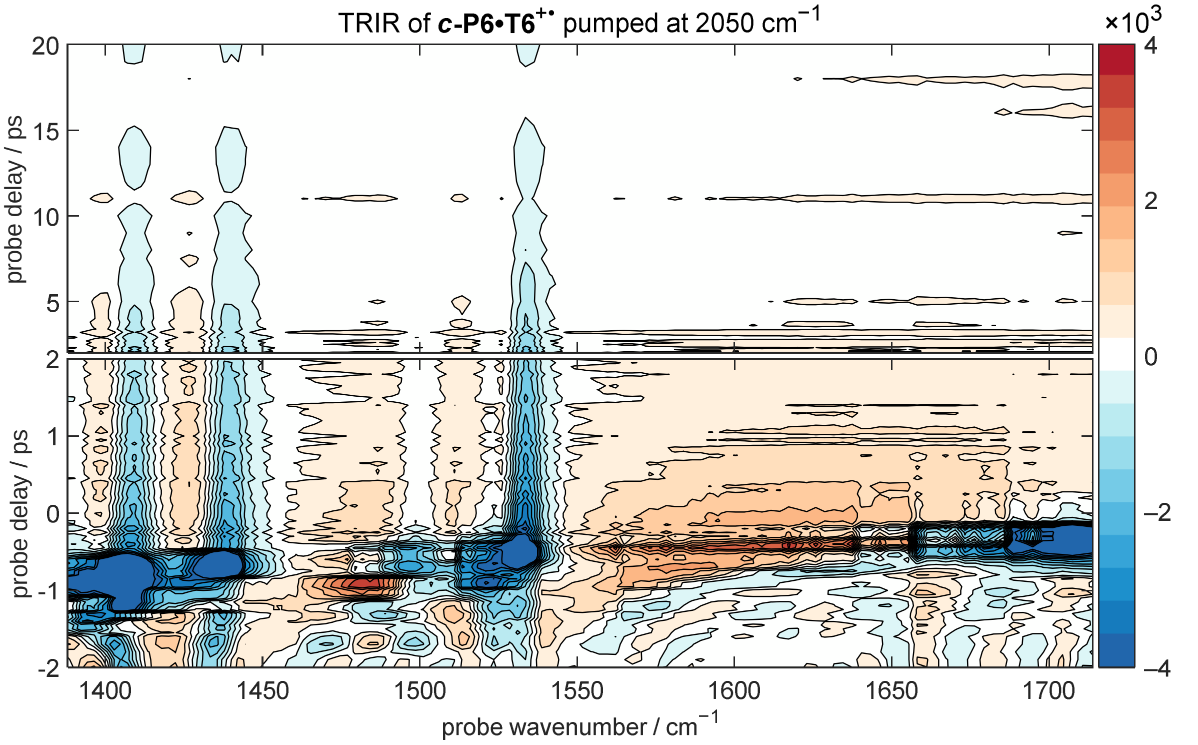

Fig. S50. Contour plot of TRIR spectra of ***c*-P6·T6$^{+•}$** pumped at ~2050 cm^{-1} and probed at 1400–1700 cm^{-1} region.

Pumping at ~2050 cm^{-1} and probing in the same region of ***c*-P6·T6$^{+•}$** gives large unresolved GSB with dip at the position of Fano anti-resonance (**Fig. S51**).

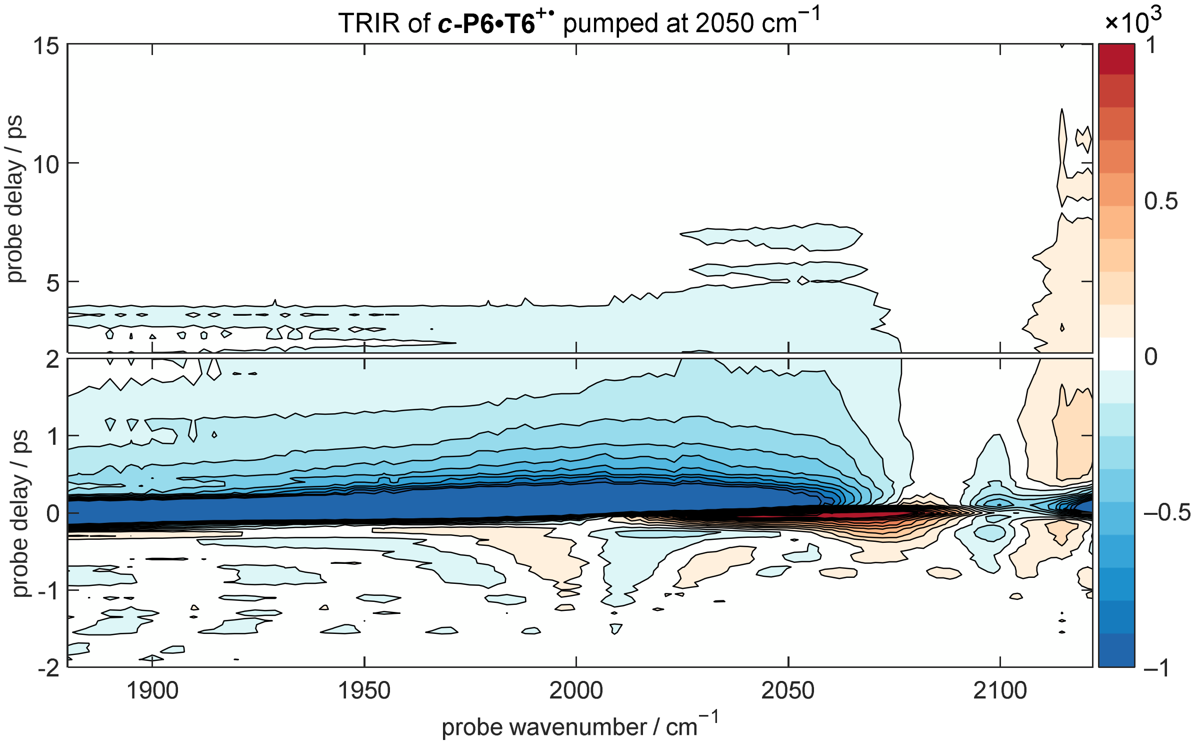

Fig. S51. Contour plot of TRIR spectra of ***c*-P6·T6$^{+•}$** pumped at ~2050 cm^{-1} and probed in the ~2050 cm^{-1} region.

4 Neutral molecules excited state absorption

TRIR spectra of neutral molecules ***l*-P2** and ***c*-P6·T6** were recorded at 3 ps delay, exciting the molecules to the S1 excited state with visible light (714 nm and 772 nm, respectively). Spectra were stitched from up to 6 probe windows (depicted in **Figs. S53, 54** in different colors) to cover area of 1300–2400 cm^{-1} showing similar features as IRAVs in mono-cations.

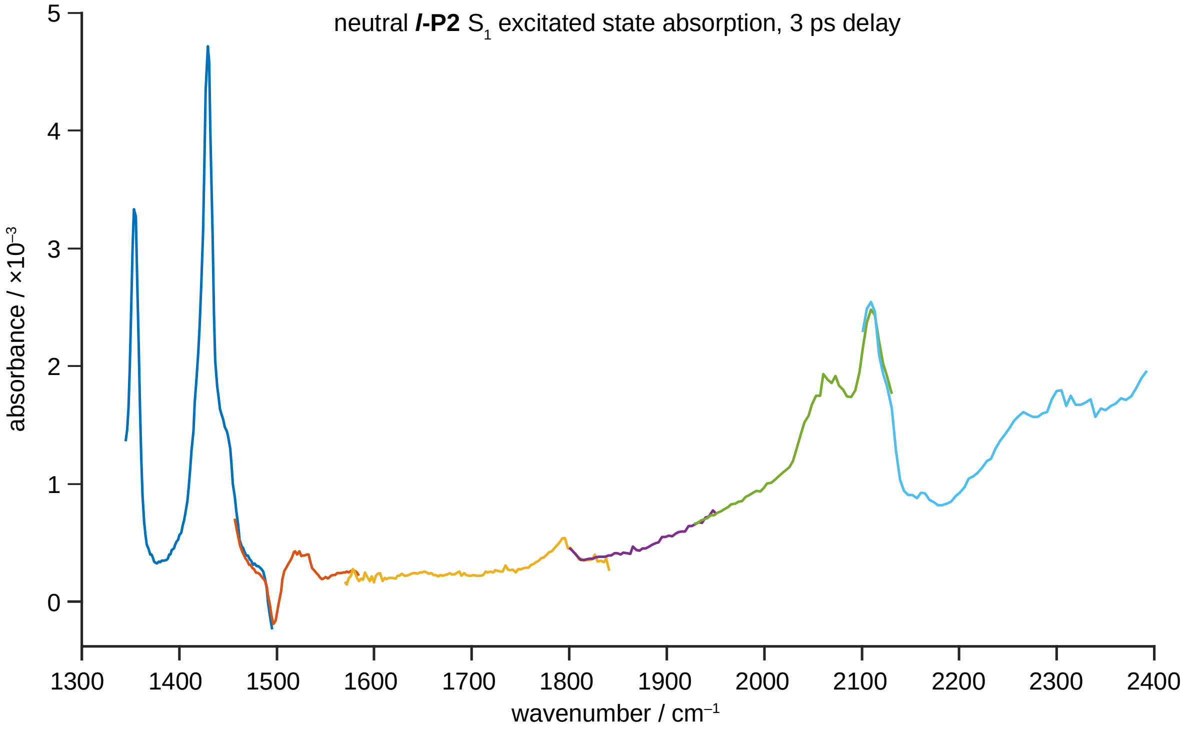

Fig. S52. Excited state absorption of neutral ***l*-P2**, pumped at 714 nm.

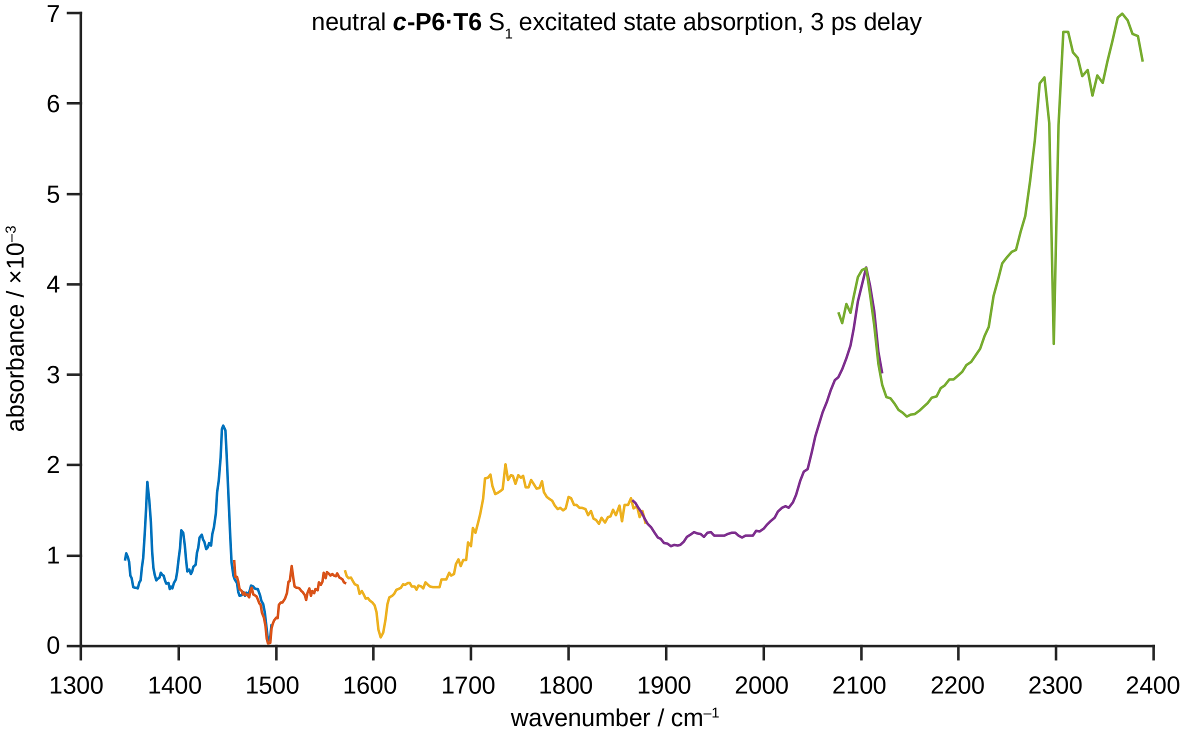

Fig. S53. Excited state absorption of neutral **c-P6·T6**, pumped at 772 nm.

5 DFT Calculations

Gaussian09/D.01 and Gaussian16/A.03,[12] Pople's basis set 6-31G*[13–21] and LC-ωPBE functional[22–24] (with ω = 0.2) were used for all calculations. Aryl side-groups and acetylene protecting groups were all truncated to —H and geometry optimizations were initiated in the maximum possible symmetry. Frequency calculation showed no imaginary frequency.

Molecular orbitals were calculated and visualized using an isosurface value of 0.01 and Gaussview software. Calculations were performed on a geometry-optimized structure of ***l*-P2**$^{+•}$ and on structures distorted along the vibrational modes at 2151, 1570 and 1373 cm^{-1}. Distorted geometries had higher electronic energy by approximately 10×($\nu_1 \rightarrow \nu_0$) cm^{-1} relative to the ground state.

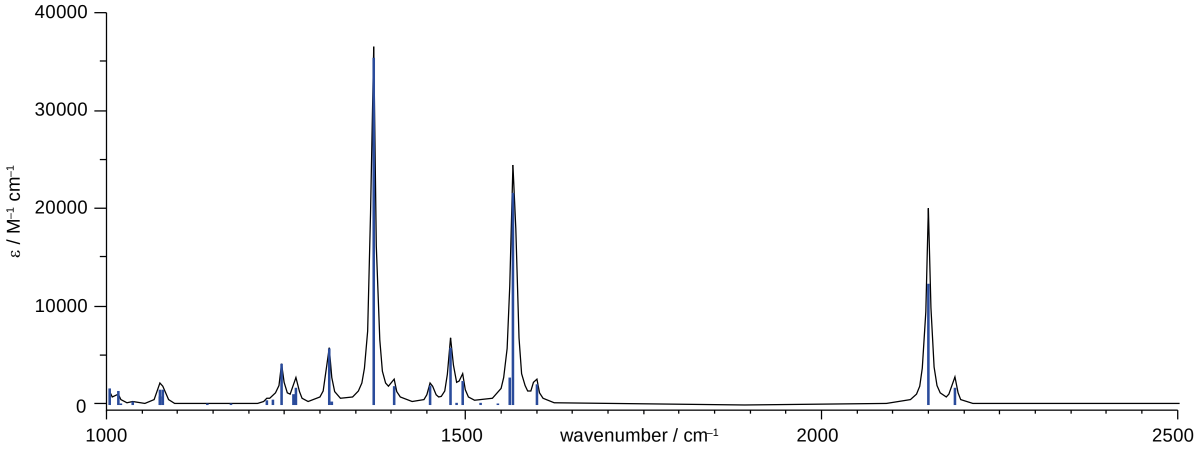

Fig. S54. IR spectrum predicted for ***l*-P2**$^{+•}$ using LC-ωPBE/6-31G* (ω = 0.2)

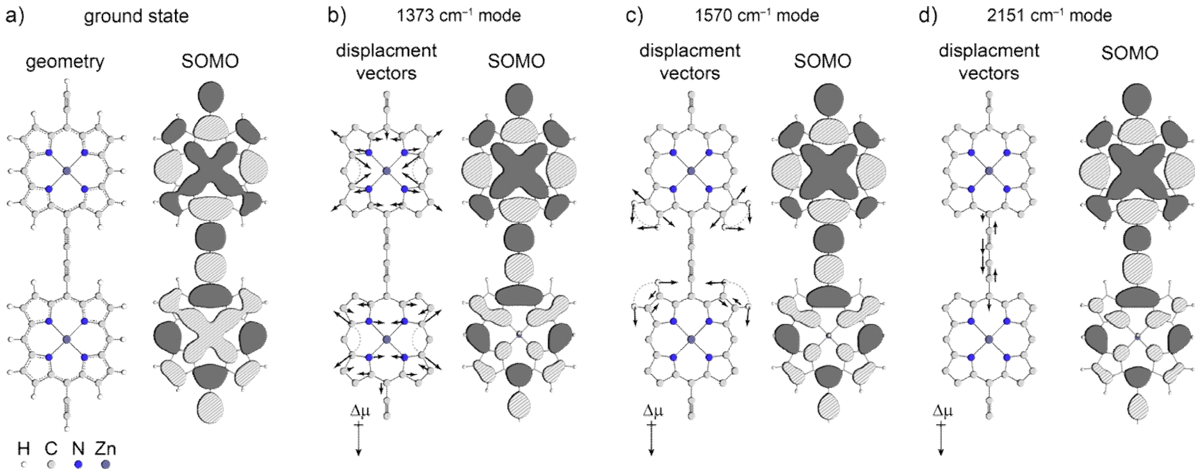

Fig. S55. a) Ground state geometry and highest singly occupied molecular orbital (SOMO) of ***l*-P2**$^{+•}$ calculated using LC-ωPBE/6-31G* (ω = 0.2). Three most intense vibrational modes: 1373 cm^{-1} b), 1570 cm^{-1} c) and 2151 cm^{-1} d) with their displacement vectors, dipole moment changes related with the vibrational mode and SOMO calculated for the distorted geometries for each vibrational mode.

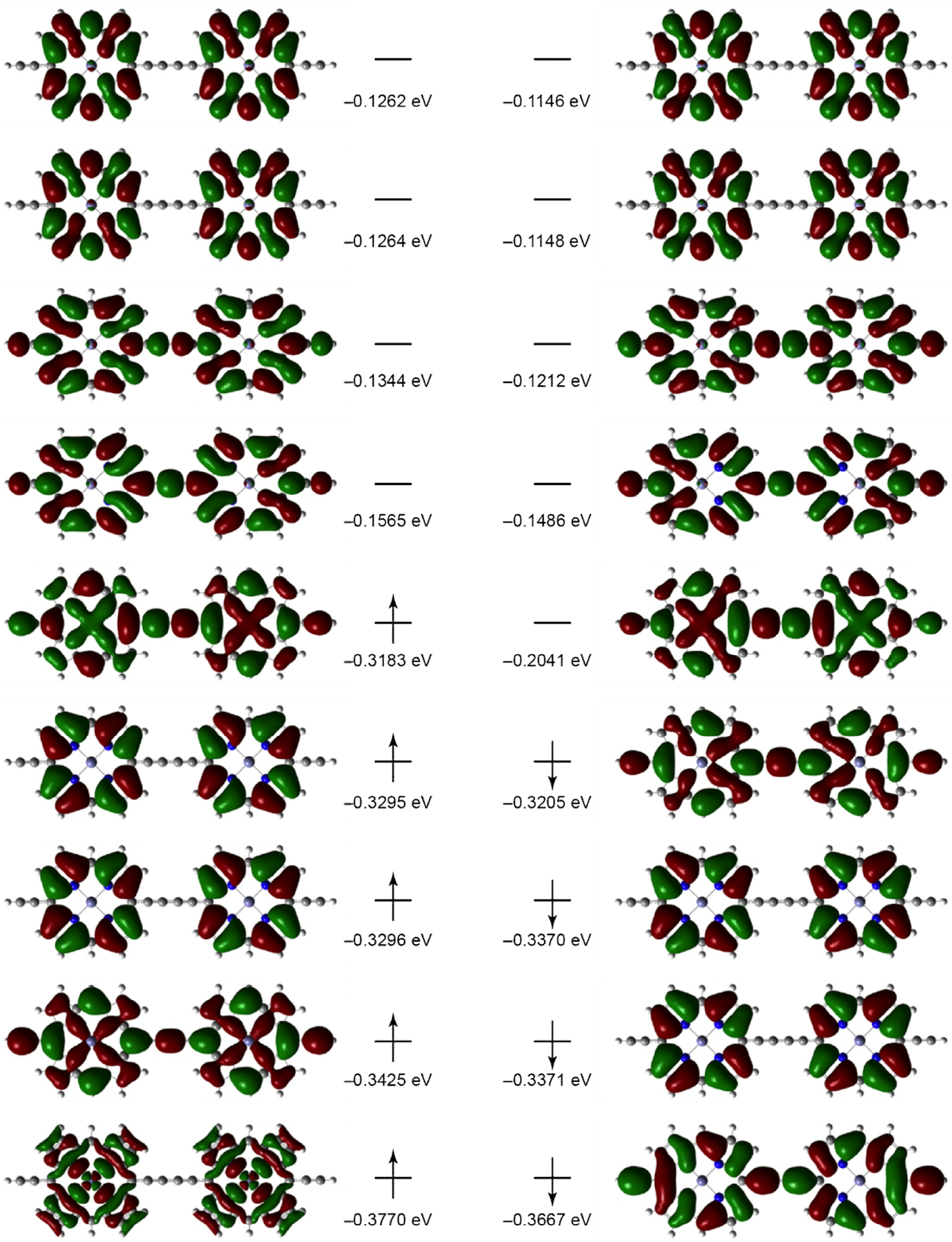

Fig. S56. Calculated MOs of ***l*-P2**$^{+•}$ in its optimal geometry (LC-ωPBE/6-31G*, ω = 0.2, MO isovalue = 0.01).

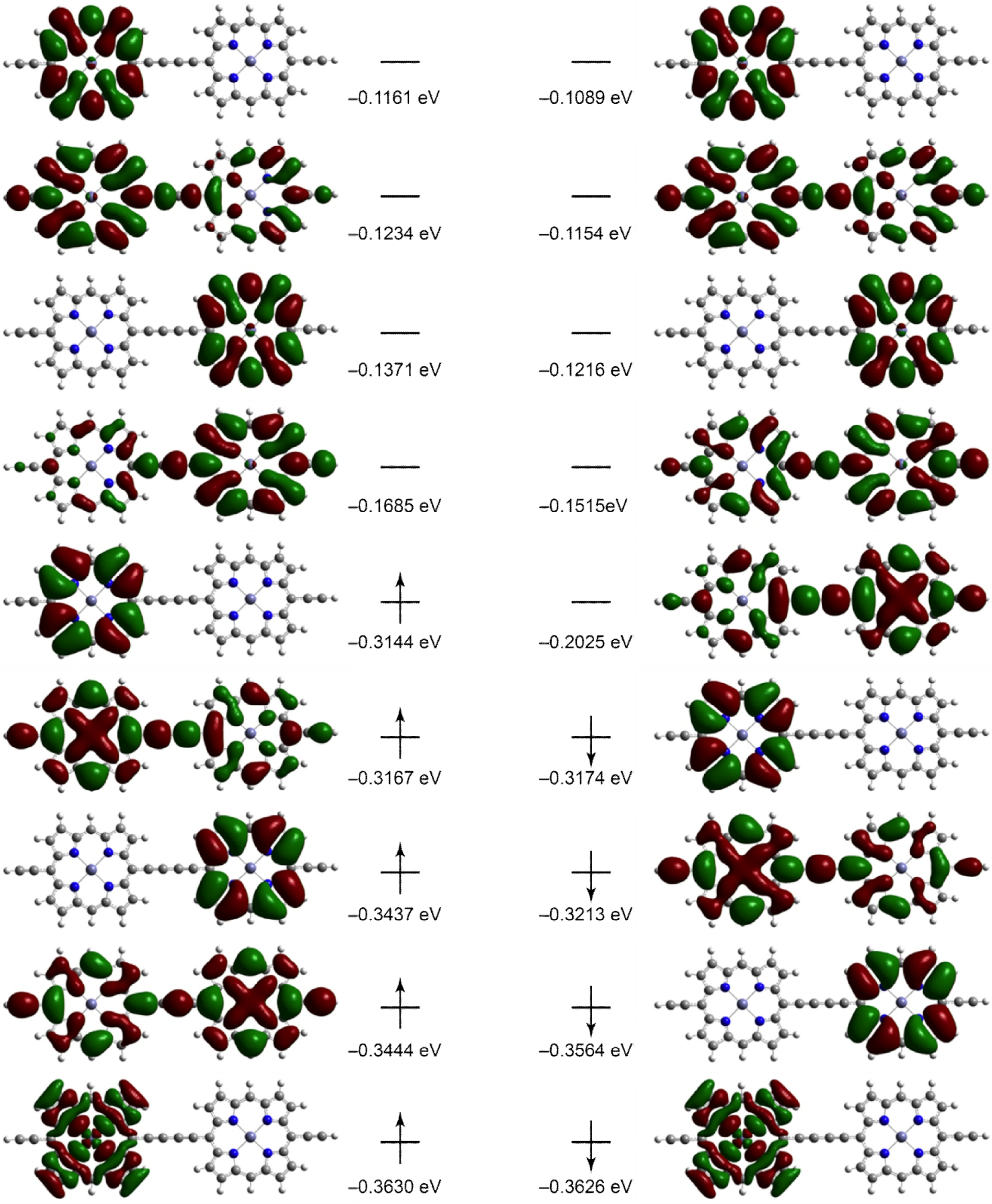

Fig. S57. Calculated MOs of ***l*-P2**$^{+\bullet}$ distorted along 1373 cm^{-1} vibration optimal geometry (LC-ωPBE/6-31G*, ω = 0.2, MO isovalue = 0.01).

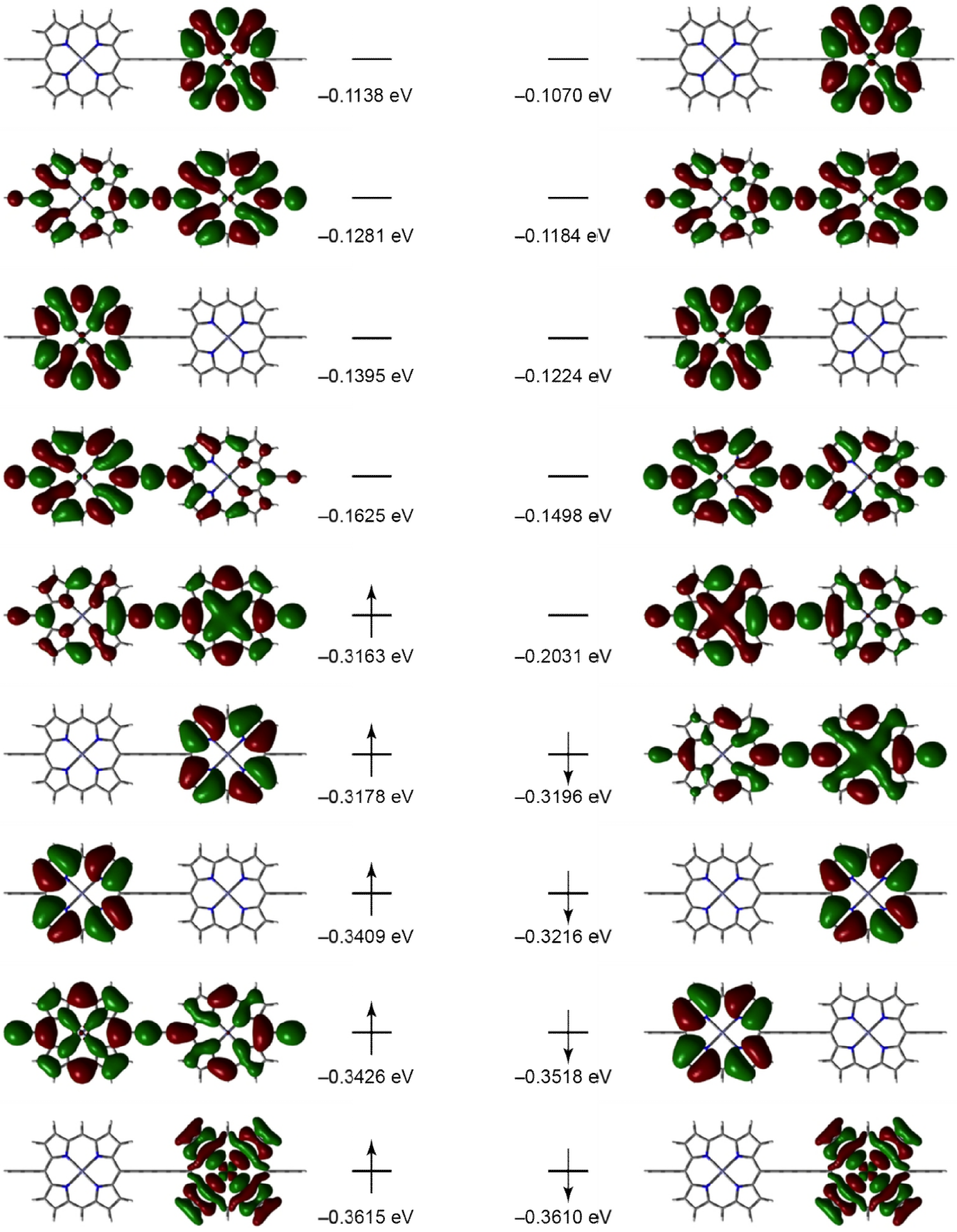

Fig. S58.Calculated MOs of ***l*****-P2**$^{+\bullet}$ distorted along 1570 cm^{-1} vibration optimal geometry (LC-ωPBE/6-31G*, ω = 0.2, MO isovalue = 0.01).

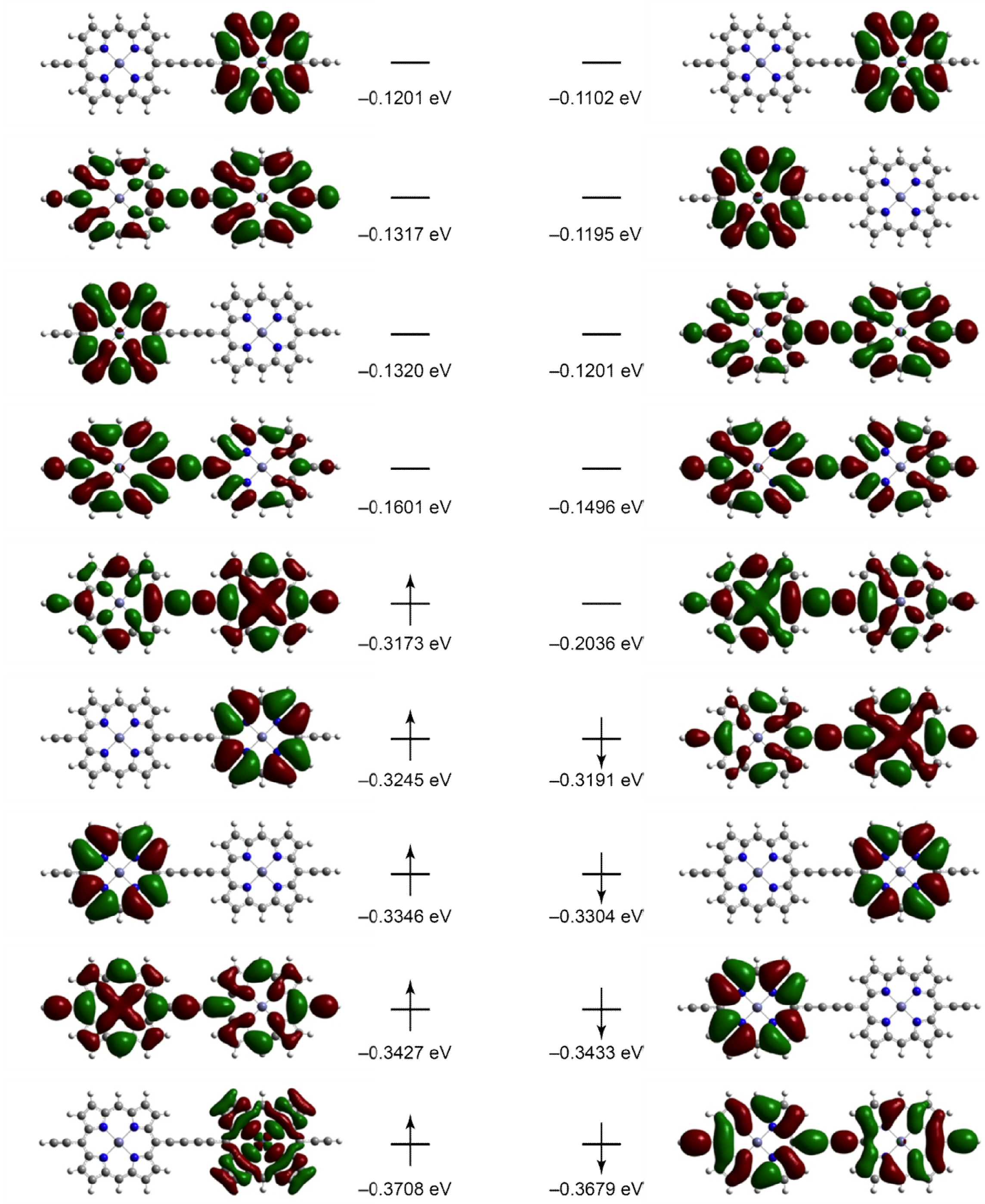

Fig. S59.Calculated MOs of ***l*-P2$^{+\bullet}$** distorted along 2151 cm^{-1} vibration optimal geometry (LC-ωPBE/6-31G*, ω = 0.2, MO isovalue = 0.01).

6 References

1 P. N. Taylor, J. Huuskonen, C. Rumbles, R. T. Aplin, E. Williams and H. L. Anderson, *Chem. Commun.*, 1998, 909–910.

2 J. K. Sprafke, D. V. Kondratuk, M. Wykes, A. L. Thompson, M. Hoffmann, R. Drevinskas, W. H. Chen, C. K. Yong, J. Kärnbratt, J. E. Bullock, M. Malfois, M. R. Wasielewski, B. Albinsson, L. M. Herz, D. Zigmantas, D. Beljonne and H. L. Anderson, *J. Am. Chem. Soc.*, 2011, **133**, 17262–17273.

3 C. E. Tait, P. Neuhaus, H. L. Anderson and C. R. Timmel, *J. Am. Chem. Soc.*, 2015, **137**, 6670–6679.

4 M. Krejčik, M. Daněk and F. Hartl, *J. Electroanal. Chem.*, 1991, **317**, 179–187.

5 M. D. Peeks, C. E. Tait, P. Neuhaus, G. M. Fischer, M. Hoffmann, R. Haver, A. Cnossen, J. R. Harmer, C. R. Timmel and H. L. Anderson, *J. Am. Chem. Soc.*, 2017, **139**, 10461–10471.

6 W. Wang, N. Zhao, Y. Geng, S. Bin Cui, J. Hauser, S. Decurtins and S. X. Liu, *RSC Adv.*, 2014, **4**, 32639–32642.

7 M. D. Peeks, C. E. Tait, P. Neuhaus, G. M. Fischer, M. Hoffmann, R. Haver, A. Cnossen, J. R. Harmer, C. R. Timmel and H. L. Anderson, *J. Am. Chem. Soc.*, 2017, **139**, 10461–10471.

8 P. Hamm and M. Zanni, *Concepts and Methods of 2D Infrared Spectroscopy*, Cambridge University Press, Cambridge, 2011.

9 N. Mouton, M. Sliwa, G. Buntinx and C. Ruckebusch, *J. Chemom.*, 2010, **24**, 424–433.

10 M. Roelfs and P. C. Kroon, *Zenodo*, 2019, June 21.

11 M. Banno, K. Iwata and H. O. Hamaguchi, *J. Chem. Phys.*, 2007, **126**, 204501.

12 M. J. Frisch, G. W. Trucks, H. B. Schlegel, G. E. Scuseria, M. A. Robb, J. R. Cheeseman, G. Scalmani, V. Barone, B. Mennucci, G. A. Petersson, H. Nakatsuji, M. Caricato, X. Li, H. P. Hratchian, A. F. Izmaylov, J. Bloino, G. Zheng, J. L. Sonnenberg, M. Hada, M. Ehara, K. Toyota, R. Fukuda, J. Hasegawa, M. Ishida, T. Nakajima, Y. Honda, O. Kitao, H. Nakai, T. Vreven, J. A. Montgomery, J. E. Peralta, F. Ogliaro, M. Bearpark, J. J. Heyd, E. Brothers, K. N. Kudin, V. N. Staroverov, R. Kobayashi, J. Normand, K. Raghavachari, A. Rendell, J. C. Burant, S. S. Iyengar, J. Tomasi, M. Cossi, N. Rega, J. M. Millam, M. Klene, J. E. Knox, J. B. Cross, V. Bakken, C. Adamo, J. Jaramillo, R. Gomperts, R. E. Stratmann, O. Yazyev, A. J. Austin, R. Cammi, C. Pomelli, J. W. Ochterski, R. L. Martin, K. Morokuma, V. G. Zakrzewski, G. A. Voth, P. Salvador, J. J. Dannenberg, S. Dapprich, A. D. Daniels, O. Farkas, J. B. Foresman, J. V Ortiz, J. Cioslowski and D. J. Fox, 2016.

13 R. Ditchfield, W. J. Hehre and J. A. Pople, *J. Chem. Phys.*, 2004, **54**, 724–728.

14 W. J. Hehre, R. Ditchfield and J. A. Pople, , DOI:10.1063/1.1677527.

15 P. C. Hariharan and J. A. Pople, *Theor. Chim. Acta*, 1973, **28**, 213–222.

16 P. C. Hariharan and J. A. Pople, *Mol. Phys.*, 1974, **27**, 209–214.

17 M. S. Gordon, *Chem. Phys. Lett.*, 1980, **76**, 163–168.

18 M. M. Francl, W. J. Pietro, W. J. Hehre, J. S. Binkley, D. J. DeFrees, J. A. Pople and M. S. Gordon, *J. Chem. Phys.*, 1982, **77**, 3654–3665.

19 J. Blaudeau, M. P. Mcgrath, L. A. Curtiss, L. Radom and I. Introduction, *October*, 1997, **2**, 5016–5021.

20 V. A. Rassolov, J. A. Pople, M. A. Ratner, T. L. Windus, V. A. Rassolov, J. A. Pople, M. A. Ratner and T. L. Windus, *J. Chem. Phys.*, 2009, **1223**, 0–7.

21 V. A. Rassolov, M. A. Ratner, J. A. Pople, P. C. Redfern and L. A. Curtiss, *J. Comput. Chem.*, 2007, **22**, 976–984.

22 O. A. Vydrov, J. Heyd, A. V. Krukau and G. E. Scuseria, *J. Chem. Phys.*, 2006, **125**, 2006.

23 O. A. Vydrov and G. E. Scuseria, *J. Chem. Phys.*, 2006, **125**, 2006.

24 O. A. Vydrov, G. E. Scuseria and J. P. Perdew, *J. Chem. Phys.*, 2007, **126**, 2007.
