# Supplementary material for: Mechanisms of IR amplification in radical cation polarons
Source: Chem Sci. 2020 Jan 22;11(8):2112–20. doi: 10.1039/c9sc05717j (PMC8150116; doi:10.1039/c9sc05717j)
Supplement: SC-011-C9SC05717J-s002 [file SC-011-C9SC05717J-s002.pdf]

Electronic Supplementary Material (ESI) for Chemical Science.
This journal is © The Royal Society of Chemistry 2020

Fundamental Mechanisms of Charge Transport in Organic Semiconductors

Organic semiconductors are important materials for a variety of applications including display devices and solar cells

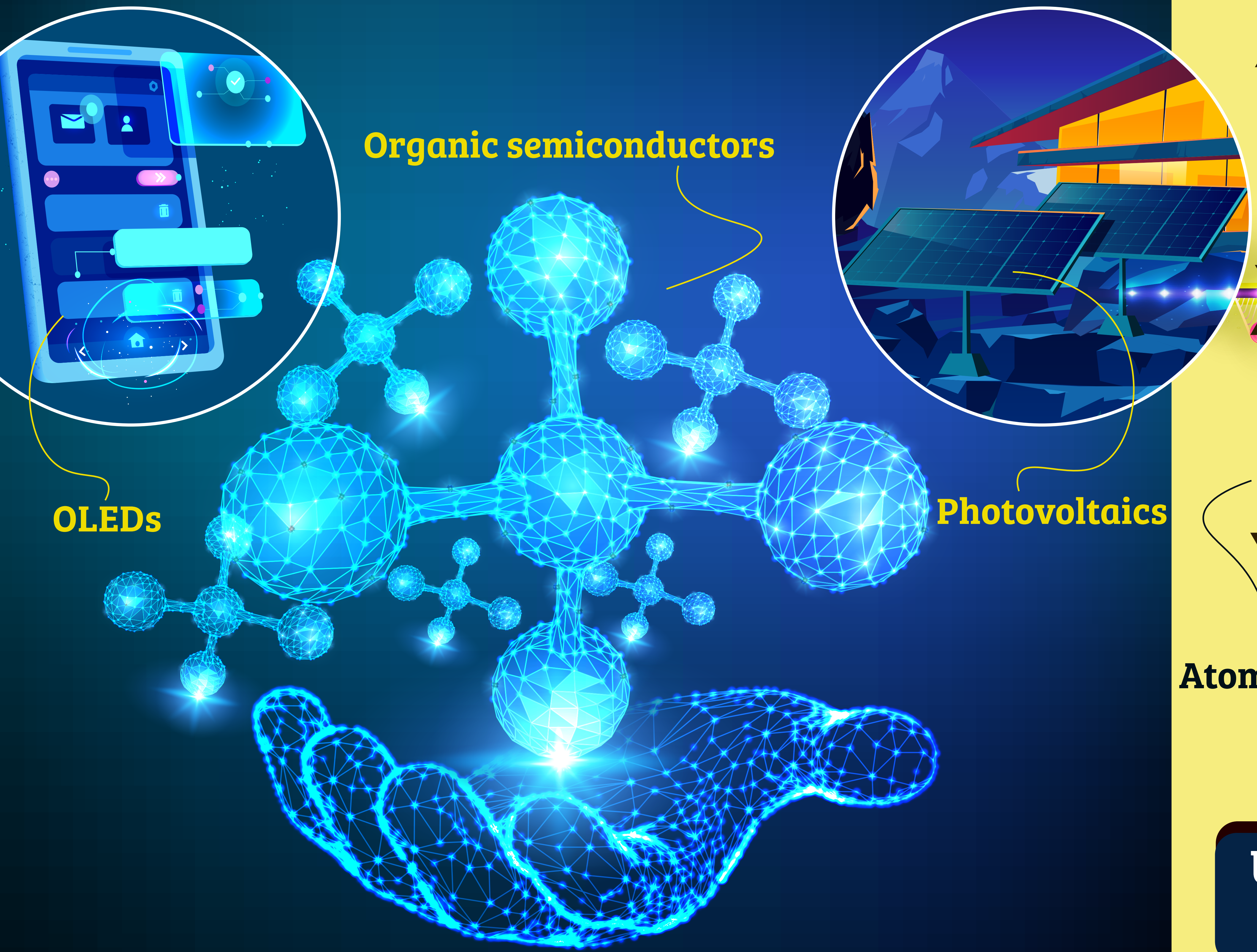

Organic semiconductor structures can be endlessly fine-tuned to change their properties

A better understanding of structure-property relationships is required to exploit the versatility of organic semiconductors

Infrared active vibrations (IRAVs) of organic semiconductors are studied using advanced ultrafast time-resolved infrared spectroscopies

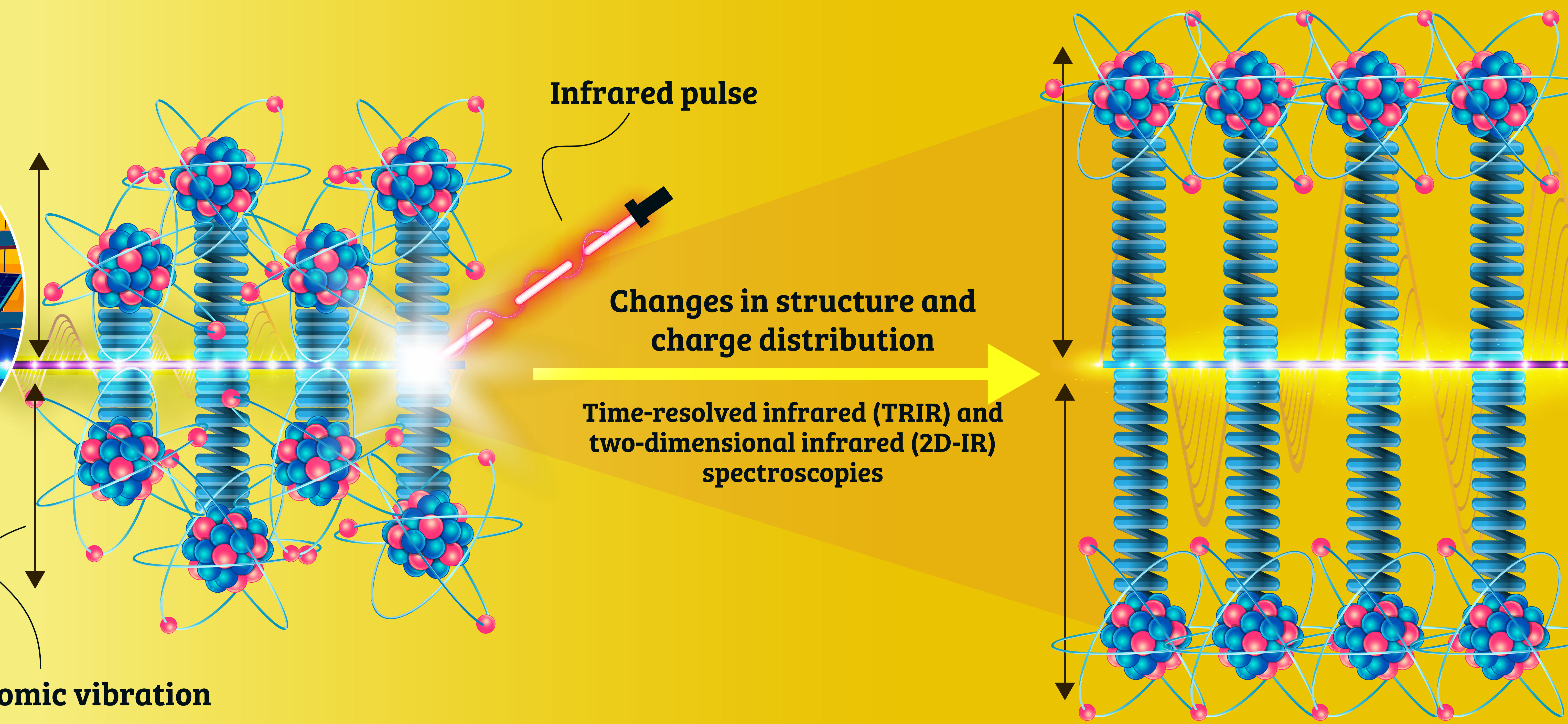

IRAVs originate from the strong coupling of charge redistribution to nuclear motion

Ultrafast time-resolved infrared spectroscopy can be used to study structure - property relationships in organic semiconductors and related molecules

Chemical Science

PICK OF THE WEEK

Mechanisms of IR Amplification in Radical Cation Polarons

Parker and Anderson *et al.* (2020) | DOI: 10.1039/C9SC05717J

ROYAL SOCIETY OF CHEMISTRY
